# Supplementary figures and images for: Unlocking Elementary Conversion Modes: ecmtool Unveils All Capabilities of Metabolic Networks
Source: Patterns (N Y). 2020 Dec 29;2(1):100177. doi: 10.1016/j.patter.2020.100177 (PMC7815953; doi:10.1016/j.patter.2020.100177)

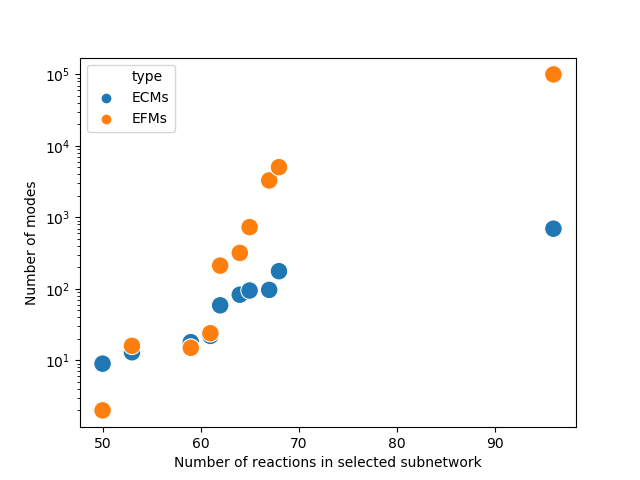

Supplement: Data S3. Zip Folder with Some Python Scripts Used for Comparison of Number of ECMs and EFMs in Various Subnetworks of the e_coli_core-Network [file mmc4.zip › result_files/comparison_n_ecms_efms.png]
