# Supplementary material for: Unlocking Elementary Conversion Modes: ecmtool Unveils All Capabilities of Metabolic Networks
Source: Patterns (N Y). 2020 Dec 29;2(1):100177. doi: 10.1016/j.patter.2020.100177 (PMC7815953; doi:10.1016/j.patter.2020.100177)
Supplement: Document S2. Article plus Supplemental Information [file mmc9.pdf]

Patterns

Article

Unlocking Elementary Conversion Modes: ecmtool Unveils All Capabilities of Metabolic Networks

Graphical Abstract

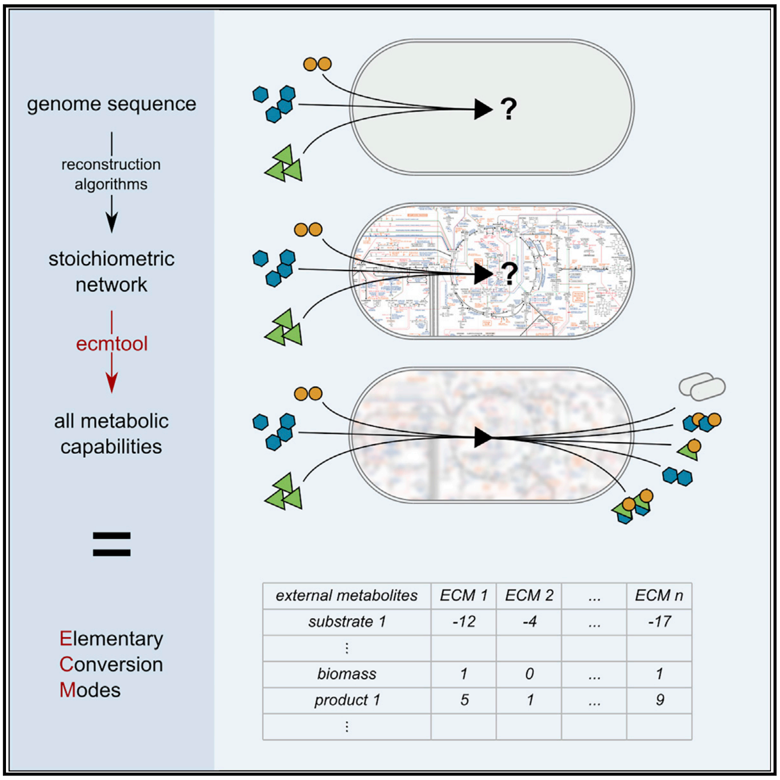

Highlights

- Elementary conversion modes (ECMs) specify all metabolic capabilities of any organism
- `Ecmtool` computes all ECMs from a reconstructed metabolic network
- ECM enumeration enables metabolic characterization of larger networks than ever
- Focusing on ECMs between relevant metabolites even enables genome-scale enumeration

Authors

Tom J. Clement, Erik B. Baalhuis, Bas Teusink, Frank J. Bruggeman, Robert Planqué, Daan H. de Groot

Correspondence

science@tomclement.nl (T.J.C.), daanhugodegroot@gmail.com (D.H.d.G.)

In Brief

Clement et al. present a ready-to-use tool that unveils metabolic blueprints of organisms from their reconstructed metabolic networks, through the computation of elementary conversion modes (ECMs). For any cell, the ECMs span all overall conversions from nutrients to new cells and the secretion of products. Therefore, the tool describes all possible effects that an organism may exert on its environment.

Check for updates

Clement et al., 2021, Patterns *2*, 100177
January 8, 2021 © 2020 The Author(s).
https://doi.org/10.1016/j.patter.2020.100177

CellPress

Patterns

CellPress
OPEN ACCESS

Article

Unlocking Elementary Conversion Modes: ecmtool Unveils All Capabilities of Metabolic Networks

Tom J. Clement,[1,3,*] Erik B. Baalhuis,[2] Bas Teusink,[1] Frank J. Bruggeman,[1] Robert Planqué,[1,2] and Daan H. de Groot[1,3,4,*]
[1]Systems Biology Lab, Amsterdam Institute of Molecular and Life Sciences, Vrije Universiteit Amsterdam, De Boelelaan 1087, 1081 HV Amsterdam, the Netherlands
[2]Department of Mathematics, Vrije Universiteit Amsterdam, De Boelelaan 1081a, 1081 HV Amsterdam, the Netherlands
[3]These authors contributed equally
[4]Lead Contact
*Correspondence: science@tomclement.nl (T.J.C.), daanhugodegroot@gmail.com (D.H.d.G.)
https://doi.org/10.1016/j.patter.2020.100177

THE BIGGER PICTURE Understanding the metabolic capabilities of cells is of profound importance. Microbial metabolism shapes global cycles of elements and cleans polluted soils. Human and pathogen metabolism affects our health. Recent advances allow for automatic reconstruction of reaction networks for any organism, which is already used in synthetic biology, (food) microbiology, and agriculture to compute optimal yields from resources to products. However, computational tools are limited to optimal states or subnetworks, leaving many capabilities of organisms hidden. Our program, `ecmtool`, creates a blueprint of any organism's metabolic functionalities, drastically improving insights obtained from genome sequences. `Ecmtool` may become essential in exploratory research, especially for studying cells that are not culturable in laboratory conditions. Ideally, elementary conversion mode enumeration will someday be a standard step after metabolic network reconstruction, achieving the metabolic characterization of all known organisms.

1 **2** 3 4 5 Proof-of-**Concept:** Data science output has been formulated, implemented, and tested for one domain/problem

SUMMARY

The metabolic capabilities of cells determine their biotechnological potential, fitness in ecosystems, pathogenic threat levels, and function in multicellular organisms. Their comprehensive experimental characterization is generally not feasible, particularly for unculturable organisms. In principle, the full range of metabolic capabilities can be computed from an organism's annotated genome using metabolic network reconstruction. However, current computational methods cannot deal with genome-scale metabolic networks. Part of the problem is that these methods aim to enumerate all metabolic pathways, while computation of all (elementally balanced) conversions between nutrients and products would suffice. Indeed, the elementary conversion modes (ECMs, defined by Urbanczik and Wagner) capture the full metabolic capabilities of a network, but the use of ECMs has not been accessible until now. We explain and extend the theory of ECMs, implement their enumeration in `ecmtool`, and illustrate their applicability. This work contributes to the elucidation of the full metabolic footprint of any cell.

INTRODUCTION

Metabolism underlies most cellular behaviors. Which chemical compounds a microbe can exploit for growth, which products it can make and at which yields is essential information for understanding the microbe's roles in ecosystems, its responses to varying conditions, and its potentials for biotechnology and bioremediation. In the case of pathogens, metabolic capabilities are informative about the niches in which they can thrive. The functioning of multicellular organisms relies on how the capabilities of different cell types complement each other. A computational method that can enumerate all metabolic capabilities of any cell, from its annotated genome sequence, is therefore of key importance.

This is an open access article under the CC BY license (http://creativecommons.org/licenses/by/4.0/).

In the pre-genomic era, a cell's metabolic capabilities were investigated using experimental physiological information and elemental balancing of nutrients and products. Cellular metabolism was seen as a black box: without having knowledge of the metabolic details, so-called macrochemical equations were calculated which specify the stoichiometry of the conversion of nutrients into biomass (cells) and by-products.[1–6] Precise measurements of heat exchange, nutrient uptake, and product formation were used to develop thermodynamic theories of cellular growth,[7] which led to the improvement of biotechnological processes.[5,8] These methods could not always be applied: they were not exhaustive, and required experimental data and basal knowledge of metabolic pathways. This information is often lacking, in particular for unculturable and extremophile microorganisms, or for cells that survive only in multi-species communities or as part of a multicellular organism. In addition, when several substrates can be consumed or multiple by-products can be produced, a unique macrochemical equation cannot be derived and the methods need to be augmented with experimental data.[3] Nowadays, in the post-genomic era, in which the genome of any organism can be sequenced, the potential exists for comprehensive and unsupervised enumeration of all macrochemical equations of any cell. Yet despite its great benefits, no such method is currently used, partially because most efforts focus on computation of a highly redundant capability set.

All metabolic reactions that can be catalyzed by a cell can be determined from the metabolic-gene annotations of its genome. This allows for the reconstruction of the metabolic network, which can nowadays be done almost purely computationally[9] (see Mendoza et al.[10] for a recent review). The resulting genome-scale metabolic networks, or genome-scale stoichiometric models, have been determined for thousands of species. Since such a model specifies all metabolic reactions, it determines all possible pathways from substrates to products, which are conveniently described by the set of all elementary flux modes (EFMs).[11–16] The enumeration of all EFMs of large metabolic networks is not possible due to a severe combinatorial explosion in their number,[17] so that most research has focused on calculating only subsets of EFMs.[18–30] However, since many EFMs share the same overall substrates-to-products conversion and, therefore, indicate the same metabolic capability, their enumeration is not always required. Instead, for many applications it suffices to focus on all possible overall conversions that a cell can catalyze.

The complete metabolic capabilities of a cell can thus be studied by focusing on all conversions from substrates to products. An exhaustive list of these is obtained by enumeration of the elementary conversion modes (ECMs), defined in 2005 by Urbanczik and Wagner.[31] ECMs are not defined in terms of the metabolic routes through the network; rather, they are defined in terms of the end results only: the feasible stoichiometries between substrates and products—the net conversion (see Box 1 for explanation). Thus, ECMs focus on the connection of an organism with its environment rather than on the metabolic pathways through which this is achieved.

ECMs can be seen as objects analogous to EFMs: the ECMs form a minimal set that generates all steady-state substrate-to-product conversions, i.e., all macrochemical equations, while the EFMs form the minimal set that generates all steady-state flux distributions. However, the set of ECMs is much smaller than the set of EFMs: first, because many different EFMs map to the same overall conversion and second, because ECMs are objects in the lower-dimensional space of external metabolite changes rather than in the space of reaction rates. For these reasons, the combinatorial explosion that prohibited the enumeration of all EFMs on a genome-scale network might disappear when enumerating ECMs.

Although ECMs were already defined in 2005,[31] and despite their potential for broad applicability, we could find only one study in which they were used.[35] This might be because the concept was never made accessible for a broad audience, even though it was rigorously defined mathematically. Mostly it might be due to the absence of a readily usable computational tool that computes ECMs for general metabolic networks.

In this work, we unlock the potential of ECMs by making the theory accessible and enumeration possible for any systems biologist. We reformulate and extend the ECM theory of Urbanczik and Wagner,[31] provide additional explanations in Boxes 1, 2, and 3, and supply extensive Supplemental Information wherein all enumeration steps are explained and mathematically supported. Most importantly, we present a Python-based enumeration program called `ecmtool`. Our software accepts metabolic models in the SBML format as input[36] and gives an exhaustive and exact list of ECMs as output. `Ecmtool` provides both an indirect and a direct method. The indirect method is based on the algorithm proposed by Urbanczik and Wagner[31] and is fast for small- to medium-scale networks; the direct method uses a novel algorithm that lends itself to massive parallelization and is therefore scalable to much larger networks. We validate the correctness of the computed ECMs on the medium-scale e_coli_core network,[37] and test the scope of `ecmtool` by enumerating the ECMs of networks of various sizes and complexity. In addition, we provide a hide method that allows focusing on the conversions between a user-defined subset of the external metabolites. This method enables the enumeration of ECMs on genome-scale models. Finally, in a collaborative, parallel study on rhizobial bacteroids, we show that ECMs can now truly be applied to gain biological insight (Schulte et al., unpublished data currently under revision).

This work contributes to closing the gap between any cell's genotype and phenotype. It offers a computational toolkit for the exhaustive determination of metabolic capabilities, and should be particularly valuable when experimental characterization is impossible because cells cannot be cultured in isolation.

RESULTS

Cells Have Orders of Magnitude Fewer Metabolic Capabilities (ECMs) Than Flux Routes (EFMs)

The number of ECMs increases much slower with metabolic network size than with the number of EFMs (Figure 2). For example, the number of elementary modes in the e_coli_core model[37] reduces from 100,274 EFMs to 689 ECMs. The number difference is likely even greater for larger genome-scale metabolic networks. This makes ECM visualization possible, which facilitates their exploration and analysis (Figure 3). This illustrates that it is more direct and efficient to enumerate ECMs, which are the metabolic capabilities of a cell, instead

CellPress
OPEN ACCESS

Box 1. Definition of ECMs

ECMs are the minimal building blocks of all net conversions by metabolic networks, and were defined by Urbanczik and Wagner.[31] To explain their definition, we start with the stoichiometry matrix N of a metabolic network. Each column of N captures for one reaction which metabolites are consumed, which are produced, and in what ratios. To facilitate the exposition, we here assume that all reversible reactions are split into a forward and backward reaction, so that all reactions in N are irreversible. Some metabolites are internal to the cell and some metabolites are external; metabolites that occur both inside and outside the cell are considered as two metabolites: one internal and one external. We denote the index set of internal metabolites by Int. The product of the stoichiometric matrix with the vector of reaction rates $\boldsymbol{v}$ gives the rates of change of all metabolite concentrations, i.e., the conversion $\dot{\boldsymbol{c}} = N\boldsymbol{v}$. Metabolism is assumed to be in steady state, so that all internal metabolite concentrations are constant: $\dot{\boldsymbol{c}}_i = 0$ for all $i \in Int$. The space of all steady-state conversions, and thus of all metabolic capabilities, is given by

$$\mathcal{C} = \left\{ \dot{\boldsymbol{c}} = N\boldsymbol{v} \middle| \dot{c}_i = 0 \text{ if } i \in Int,\ v_j \geq 0 \text{ for all } j \right\}. \quad \text{(Equation 1)}$$

This space is called the conversion cone and should not be confused with the flux cone which comprises all steady-state fluxes. In fact, the conversion cone is the result of multiplying all points in the flux cone with the stoichiometric matrix (see Supplemental Information Section 2 for further explanation).

Definition 1. *The set of elementary conversion modes (ECMs) is the minimal set of conversions* $\{ecm_1, \ldots, ecm_K\}$ *such that each steady-state conversion can be written as a positive sum of ECMs, without the production of any external metabolite being canceled in that sum.*

Some readers might note that this definition of ECMs is similar to the definition of EFMs. This is because both can be defined as elementary vectors (more precisely as conformally non-decomposable vectors[31–34]): ECMs are the elementary vectors of the conversion cone, while EFMs are the elementary vectors of the flux cone. The values in an ECM indicate the changes of metabolite concentrations, while the values in an EFM indicate reaction rates.

We will explain the two parts of Definition 1 using the toy network example of Figure 1, in which the external metabolites A, B, and BM are interconverted via internal metabolites C, D, and E.

All steady-state conversions together form the conversion cone, which is a "convex polyhedral cone" (shaded area in Figure 1B). As a consequence, the steady-state conversions can be fully described by the extreme rays of this cone (blue and green in the figure). Indeed, any steady-state conversion can be written as a positive sum of the extreme rays. By the first part of Definition 1, this means that these extreme rays are ECMs. The example, therefore, has at least two ECMs: A → B (blue) and 2B → BM (green).

It is important to note that any *positive* sum of steady-state conversions is again a steady-state conversion. This makes sense in biological terms: a conversion lies in the cone if there exists a set of reactions that gives rise to the conversion, and satisfies the irreversibility and steady-state constraints from Equation 1. So, if we have several sets of reactions that correspond to conversions, their sum will correspond to the summed conversion. However, a sum in which some extreme conversions are added *negatively* does not necessarily result in a feasible conversion, because the resulting conversion might not be feasible without using an irreversible reaction in the negative direction.

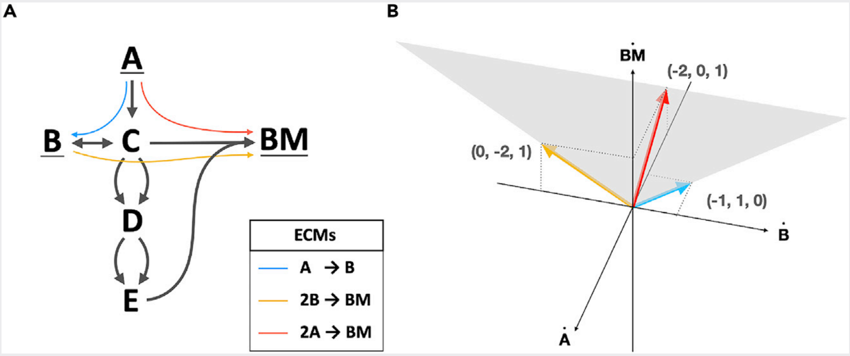

Figure 1. ECMs Are the Minimal Building Blocks of All Net Conversions by Metabolic Networks

For a Figure360 author presentation of this figure, see https://doi.org/10.1016/j.patter.2020.100177.

(A) The Elementary conversion modes for a small network are shown in blue, green, and red. Note that the red ECM can be written as a positive combination of the blue and green ECM, but that this cancels the production of B.

(B) The cone of steady-state conversions is shown in gray and is spanned by the blue and green ECM. The red ECM lies in the interior of the cone on the intersection with the $\dot{B} = 0$ plane.

Figure360▷

(Continued on next page)

Box 1. *Continued*

Now consider the conversion 2 A → BM (red). This conversion can be written as a positive sum of the two previously found ECMs: 2(A→ B) + (2 B → BM) = (2 A → BM). However, in summing these ECMs, the metabolite *B* is cancelled, since it is produced *and* consumed. Since, the ECMs are intended to capture a complete set of minimal building blocks of biologically realistic conversions, taking only the extreme rays does not suffice: we also want to describe the possibility of producing *BM* from *A* without simultaneously excreting and consuming *B*. The second part of the ECM-definition therefore ensures that these conversions are added to the set of ECMs as well: the ECMs should generate all conversions *without cancellation of the production of any metabolite*. If we would take a combination of the blue and green extreme conversions, this would always (partly) cancel the production of *B*, since *B* is produced in the blue conversion and consumed in the green conversion. Therefore, the red conversion is also an ECM: since this conversion does not produce or consume *B*, a positive combination with the other ECMs does not induce a cancellation. In total, we thus have three conversions, as listed in Figure 1A.

In mathematical terms, one could obtain the full set of ECMs by calculating the extreme conversions per orthant, and then taking the union of all these extreme conversions. The requirement that no metabolite production is cancelled, implies that all steady-state conversions can be written as a positive sum of ECMs in which each metabolite is either produced by all ECMs in the sum, or consumed by all ECMs in the sum. In this manner, cancellations no longer occur (see [33]).

of EFMs, which are flux routes that often have an identical metabolic capability, i.e., net conversion of cellular nutrients into products (Figure 2A).

A major advantage of ECMs is that they can be computed for metabolic networks for which EFM enumeration is not possible. For example, we found 874,236 possible ECMs for the pathogen *Helicobacter pylori* in a minimal medium (iIT_341;[38] 485 metabolites and 554 reactions), while EFM enumeration ran into memory errors, most likely due to the enormous number of EFMs in this model (the full set of ECMs is available upon request). We note that a set of hundreds of thousands of ECMs might appear difficult to analyze, but the user can easily filter out a relevant subset once such a set is obtained (see Figure S1 for an example).

Summarizing, the enumeration of ECMs by `ecmtool` allows for the determination of all the metabolic capabilities of metabolic networks for which EFM enumeration is no longer feasible. We did find that the number of ECMs in the genome-scale *Escherichia coli* network iJR904[39] (761 metabolites, 1,075 reactions) is still too large to be computed by `ecmtool`. However, even for models of this size `ecmtool` still provides useful information. In Box 2 and Figures 5 and 6, we show how focusing on essential information allows networks of this size to be analyzed.

Validation of ecmtool for ECM Enumeration

We validated the results of `ecmtool` in several ways. First, we have computed the ECMs on many small models for which we could still check the correctness and completeness of the results by hand. Second, we used the e_coli_core-model, for which we could still use the set of EFMs enumerated by efmtool, to validate our results. The MATLAB code that we used for this validation is provided as a supplemental file.

The correct set of ECMs should satisfy three properties: (1) each ECM must be a steady-state conversion, (2) each ECM must be an elementary vector, and (3) each steady-state conversion must be a positive combination of ECMs without metabolites being canceled in the sum.

We confirmed that all computed ECMs are steady-state conversions by checking that the net production of internal metabolites equals zero, and that there exists a combination of metabolic reactions that gives rise to the ECM. Then, according to the definition of ECMs given in Box 1, we proved that each ECM is elementary by showing that it cannot be written as a positive sum of the other ECMs without the production of any external metabolite being canceled.

The third property was more difficult to validate, because how can we prove for *all* steady-state conversions that they can be written as a combination of ECMs? We chose to use the set of EFMs calculated by efmtool. This set spans all possible steady-state flux combinations the metabolic network allows. For each EFM, we then calculated its overall conversion and tried to write this conversion as a combination of ECMs. If we allowed for an error of 10^{-7}, each conversion could be decomposed into ECMs. This error margin was necessary because the results from efmtool are affected by round-off errors. The computed ECMs do not suffer from round-off errors because the computation by `ecmtool` uses fractions only. Although this slows down many of the calculations, this is necessary to maintain the accuracy of the computed ECMs. For example, for the Double Description (DD) method it is known that round-off errors can grow to a non-negligible size.[40]

Above, we explained and validated that `ecmtool` finds all ECMs, given an annotated genome. The annotation is necessary for the reconstruction of the metabolic network. Strictly speaking, this minimally requires the annotation of the metabolic genes. Since the annotation of a genome is not always complete, we cannot guarantee that all metabolic capabilities encoded on the genome are found. We can guarantee that all conversions are found of the genome-derived metabolic network.

Focusing on Subsets of Metabolites Enables Genome-Scale Calculation of Metabolic Capabilities

Focusing on the stoichiometric relations between metabolites of major importance by hiding external metabolites of minor importance is a powerful way to scale up the size of metabolic networks that can be dealt with in `ecmtool`. The ECMs that are obtained now span all possible relations between the non-hidden metabolites but no longer give information about what happens to the hidden metabolites (see Box 2 for a more elaborate explanation). Importantly, the steady-state assumption remains satisfied and all hidden metabolites can be produced or consumed, even though this production or consumption is not reported.

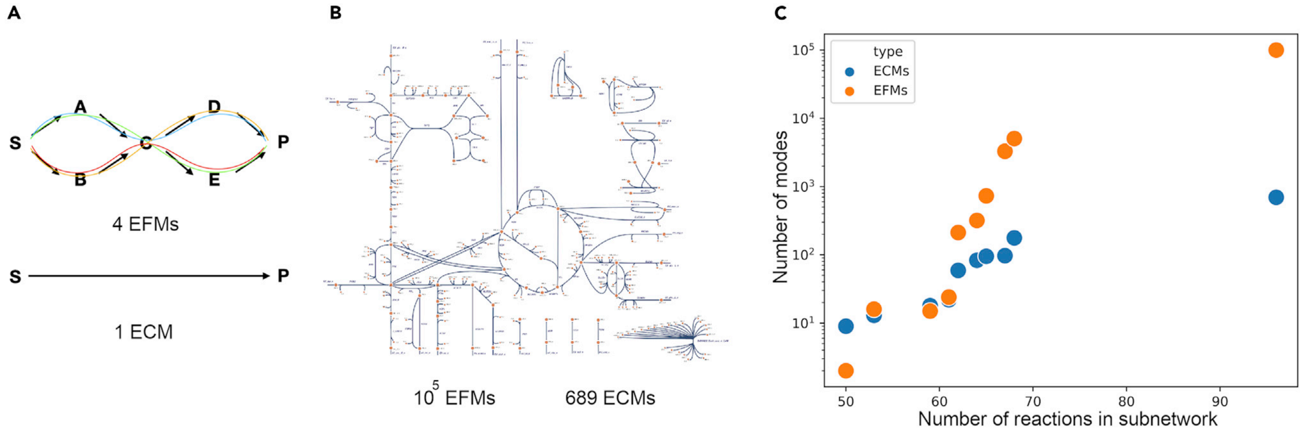

Figure 2. The Number of ECMs Remains Orders of Magnitude Lower than the Number of EFMs

(A) Because many different EFMs refer to the same overall metabolic capability, the number of ECMs is much lower than the number of EFMs.

(B) EFM-versus-ECM numbers in the e_coli_core network.

(C) Subnetworks of the e_coli_core network were selected (see Supplemental Information 10.1) to illustrate how the number of ECMs and EFMs scale with network size.

To illustrate how the hide method can help one to focus on the most important metabolic capabilities of a network, we focused on the minimal growth strategies that the pathogen *H. pylori* can employ. We took the iIT341 model for which we already calculated the full set of ECMs (see Figure S1) and hid all information about product secretion (Figure 5). The 3,652 ECMs that were obtained thus span all possible proportions in which the different nutrients can be consumed. The results show that the only mutual dependency between the uptake of different nutrients is between D-alanine and L-alanine, one of which should always be consumed. This independence indicates a modular design of the nutrient uptake system of *H. pylori*, which might benefit its flexibility when living in the human stomach.

If we focus only on the conversion of glucose and oxygen into biomass, we could even compute the ECMs for a genome-scale model of *E. coli*: iJR904,[39] containing 761 metabolites and 1,075 reactions (Figure 6). According to the authors' definition, the resulting ECMs form a minimal spanning set of all feasible conversions from glucose and oxygen to biomass. This implies that the set of ECMs contains the most "extreme" conversions. Therefore, we can use them to draw the full Pareto front between the biomass yield on glucose and on oxygen, extending a method used by Carlson and Srienc to genome-scale models.[41] It turns out that this Pareto front is completely determined by 12 ECMs. For each of these ECMs, we can find a combination of reaction rates that gives rise to this conversion. In doing so, we obtain 12 states of metabolism that fully determine *E. coli*'s flexibility to optimize its growth rate in glucose- and oxygen-limited conditions. A flux balance analysis whereby glucose and oxygen uptake is constrained and biomass production is maximized will always result in a combination of these metabolic states.

Case Study: A Metabolic Capability Study of an Unculturable *Rhizobium* Strain with ecmtool

Rhizobia are soil bacteria that can induce formation of nodule structures on plant roots, in which they differentiate into non-dividing bacteroids. Bacteroids fix atmospheric nitrogen into ammonia and make this available to the plant in exchange for carbon in the form of dicarboxylates.[42] Although a metabolic network was reconstructed, physiological information about rhizobial bacteroids is lacking because they are difficult to isolate and extremely fragile.[43] In addition, analyzing the metabolic network with an optimization approach such as flux balance analysis[44] is unfavorable because it is unclear what the optimization objective would be. After personal correspondence, `ecmtool` was used by Schulte et al. to enumerate the metabolic capabilities of *Rhizobium leguminosarum* (Schulte et al., unpublished data currently under revision). This aided in exposing the role of oxygen supply in the observed amino acid secretion and carbon polymer synthesis by bacteroids, and in quantitatively reproducing the carbon cost of biological nitrogen fixation.

DISCUSSION

Relevance of ECMs

Our method enumerates and quantifies, for any organism for which a metabolic reconstruction has been made, all possible stoichiometric relations between substrates, products, and biomass. This method does not rely on any optimality assumption, nor does it require experimentally obtained physiological information. It uncovers the full metabolic capability of an organism, and with that the metabolic footprint that an organism may leave in its environment.

ECM enumeration stands in a long tradition of methods that pursue this goal.[45] Some of these methods attempt to find an exhaustive list of reaction pathways that a cell is capable of, for example calculating extreme currents,[46] EFMs,[11] or elementary pathways.[47] These methods all have in common that scaling to genome-scale metabolic networks is impossible because of the rapid growth of the number of pathways with network size.[17] Other methods try to view the cell as a black box and focus on what is consumed and what is produced, leading to the concepts of macrochemical equations,[3,5] direct overall reactions,[2] and eventually to ECMs.[31] ECM enumeration is the only

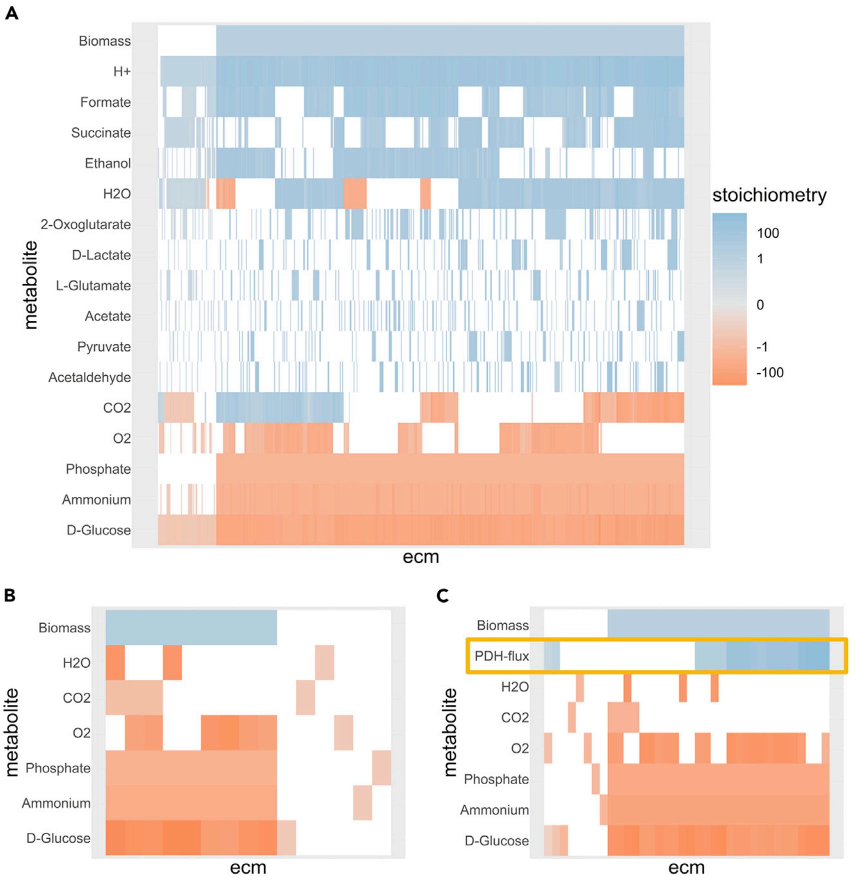

Figure 3. The Full Metabolic Potential of the e_coli_core-Model

(A) The ECMs of the full model are shown as the different columns; each row corresponds to a different external metabolite. The color scale indicates the stoichiometric coefficient of the metabolite in the conversion: blue for production and red for consumption. The coefficients were log-transformed to allow for visualization of differences in both large and small coefficients (details and R-code can be found in Supplemental Information 10.2); small values are shown in gray while zero values are white. Of the 689 elementary conversions, 613 lead to the production of biomass. These ECMs were normalized to fix the biomass production at 1, while the other ECMs were normalized such that the sum of absolute coefficients is 1.

(B) If we use the hide method, explained in Box 2, to hide the production of metabolites, we get 15 ECMs that span all possible ratios in which substrates can be converted into biomass. This smaller set of ECMs is easier to compute and easier to explore, while the steady-state assumption is still satisfied in the whole network. So even though the secretion of products is not reported, it has been implicitly taken into account, so that all relations between substrates shown in (A) are captured in (B).

(C) If we use the tag method, also explained in Box 2, to report the activity of the pyruvate dehydrogenase (PDH) reaction, we find 36 ECMs that summarize all possibilities. It can be seen that the PDH reaction is not essential for growth but seems to be necessary for efficient growth on glucose, since the uptake of glucose is generally lower when PDH is active.

method that provides a complete set of metabolic capabilities, takes reaction irreversibility into account, and scales to genome-scale networks.

Applications of ECM Enumeration

The enumeration of ECMs facilitates the exploratory study of metabolic networks: investigation of the ECMs could spark new hypotheses and show unexpected connections. It therefore complements optimization approaches like such as flux balance analysis (FBA)[44] that are efficient at answering questions already known beforehand. Even in the case that optimization approaches are more efficient, elementary mode analysis provides additional insight. For example, EFM analysis was used to understand an adaptive growth strategy of *Lactobacillus plantarum* that was observed experimentally and predicted by FBA.[48] In this specific case, the analysis could be restricted to primary metabolism which facilitated the EFM computation, but this restriction is often biologically unreasonable. In the future ECMs could replace EFMs, such that this approach can be more generally applied. Carlson and Srienc[41] used the set of EFMs in a relatively small *E. coli* model to investigate optimized *E. coli* growth in carbon- and oxygen-limited conditions. Using this approach, they could simplify their analysis by selecting four EFMs that together determined all optimal growth strategies in different glucose- and oxygen-limited conditions. In Figure 6 we showed that with `ecmtool` this approach can be generalized to genome-scale models.

Most analyses of metabolic networks require a priori physiological information that is often not available. For example, it is often required to impose constraints on exchange fluxes to choose a reaction rate that needs to be optimized, or at least to know which metabolites can be produced.[3] This hinders the investigation of species that are insufficiently characterized and difficult to culture. Moreover, for many organisms it is doubtful whether reaction rates are optimized at all, for example for pathogens or the composing cells of higher eukaryotes. ECMs do not require extensive information, solely a reconstructed metabolic network. The decisive role that ECM enumeration can play in the study of unculturable and non-optimized organisms is exemplified by the recent application of `ecmtool` to investigate the symbiotic relationship of unculturable bacteroids with plants (Schulte et al., unpublished data currently under revision).

An overview of all feasible overall reactions might furthermore be useful when studying interacting species, such as crossfeeding species, host-pathogen interactions, or multi-species communities. The possible interactions are determined by what is consumed and produced by the individual species, which is exactly the information offered by the ECMs. Indeed, knowing the capabilities of one and the incapabilities of another might lay bare dependencies on which a stable community is built.

Methods to Scale ECM Computation Even Further

Although ECM computation increases the size of models for which metabolic capabilities can be charted, all ECMs of

Box 2. Hiding and Tagging Enables Focusing on the Most Important Metabolic Conversions

In `ecmtool`, the user can choose to compute only the stoichiometric relations between a subset of the external metabolites by "hiding" the other external metabolites. The resulting set of ECMs still gives a full summary of these relations and complies with the steady-state assumption on the full metabolic network. The consumption and production of the hidden metabolites still occurs but is not reported. As a result, the reported ECMs are not necessarily mass-balanced, which is emphasized by the question marks in Figure 4A. An ECM computed with the hide method thus gives a ratio in which the non-hidden metabolites can appear in a conversion, but it does not give any information about which hidden metabolites are consumed or produced in such a conversion. In return, the hide method facilitates ECM enumeration on much larger networks because fewer ECMs are needed to describe all conversion relations between the smaller set of non-hidden metabolites. Therefore, ECM enumeration with hidden metabolites can take an organism's full metabolic complexity and summarize its metabolic capabilities regarding a few variables that are of interest.

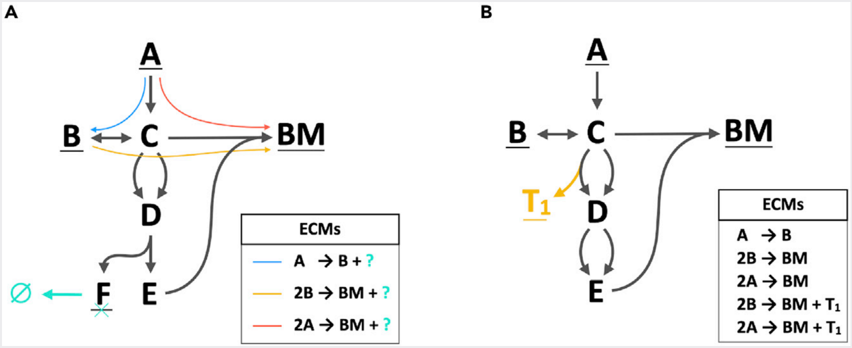

Figure 4. The Hide- and Tag-Methods Enable the Study of the Relations between the Most Relevant Metabolites and Reactions

For a Figure360 author presentation of this figure, see https://doi.org/10.1016/j.patter.2020.100177.

(A) Information about the production of F can be ignored if F is marked as internal and a virtual reaction (cyan) is added that converts metabolite F into nothing. This strategy aids in fast computation, because the resulting set of ECMs is generally smaller (see also the worked-out example in Methods).

(B) Information about the usage of a reaction can be uncovered by coupling the production of a virtual metabolite (T_1, shown in green). The coefficient of T_1 in the resulting ECMs denotes the rate of the reaction of interest.

Figure360 ▷

In Figure 4A we show how metabolite F can be hidden in the ECM computation by adding a reaction that converts it to nothing (sometimes called a demand reaction). In general, a metabolite is hidden by adding a reaction that creates it from "nothing," turns it into "nothing," or both, depending on whether the metabolite can only be consumed, only produced, or both, respectively. The metabolite is then marked as an internal metabolite, so that the steady-state assumption is imposed. The added reaction can always make sure that the net consumption or production of the metabolite is zero. As a result, the hidden metabolite will vanish from the computations at an early stage of the enumeration, thereby reducing computation time. We illustrate this in the worked-out enumeration example in Box 3.

In the example of Figure 4A, we obtain the conversions between non-hidden metabolites A, B, and BM, ignoring the information about whether or not F is produced during these conversions. If metabolites are hidden, the computed conversions should be interpreted with care, acknowledging that the reported conversions are possibly not elementally balanced (since the hidden metabolites are excluded from this report). In the example, we emphasize that we do not know whether F was produced in the conversion by adding question marks on the right side of the conversion notation. If metabolites that can be consumed by the cell are also hidden, question marks should be placed on the left side as well.

Besides hiding metabolites of minor importance, we can keep track of reaction rates of major importance. The tag method, suggested by Urbanczik and Wagner,[31] adds a virtual external metabolite that is produced whenever the reaction of interest is used. As a result, one unit of virtual metabolite is produced when the tagged reaction runs at a rate of 1. Since an ECM reports the stoichiometric coefficients of all metabolites in the conversion, the coefficient of the virtual metabolite in the ECM reflects the rate at which the tagged reaction must run to produce the conversion. This method will show to which conversions the reaction of interest contributes, possibly providing valuable information about the essentiality of that reaction. In Figure 4B, we show an example of such reaction tagging. One of two reactions from C to D is extended to produce virtual metabolite T_1, resulting in the reaction C → D + T1. Any conversion that uses the reaction of interest produces T_1, and its coefficient in the conversion is equal to the reaction rate.

In Figure 3 we illustrate the hide- and tag-methods in the e_coli_core model to respectively highlight the different possible combinations of growth substrates, and the necessity of the pyruvate dehydrogenase reaction in these conversions.

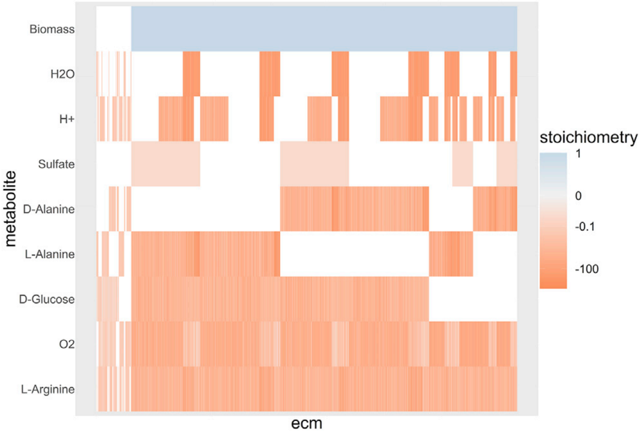

Figure 5. Focusing on Substrate Uptake Shows the Minimal Needs of *Helicobacter pylori*

We computed the ECMs, shown as different columns, for the iIT341 model by allowing for the uptake of all metabolites of a supposedly minimal medium proposed by the developers of the model (MinII from Thiele et al.[38]). All output metabolites were hidden, using the hide method outlined in Box 2. The uptake of nine substrates is not shown here because these were equal for all ECMs, indicating that these are directly coupled to biomass formation. The color scale indicates the log-transformed coefficients of the metabolites in the conversion, where metabolite production is shown in blue and consumption in red (details and R-code can be found in Supplemental Information, Section 10.2). The ECMs are normalized such that biomass production, if non-zero, is 1, otherwise the sum of the absolute coefficients is fixed at 1. The ECMs were clustered using hierarchical clustering. The block-like ordering of the ECMs indicates that substrate usage of *H*. *pylori* is largely modular: the uptake of one substrate seems independent of the uptake of another.

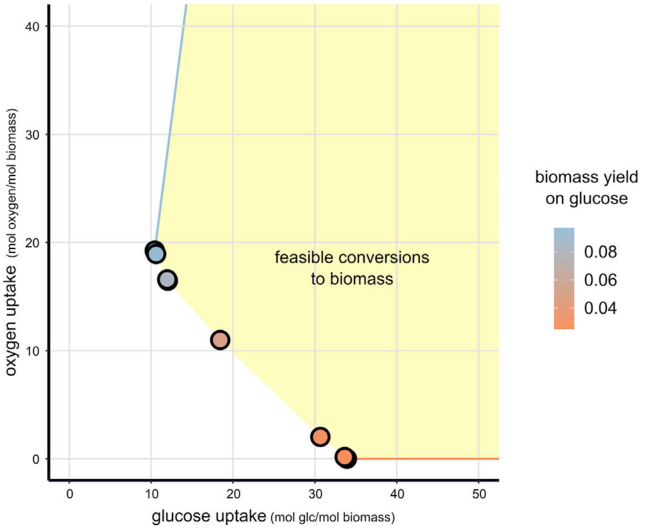

Figure 6. Few Conversions from Glucose and Oxygen to Biomass Cover *E*. *coli*'s Full Flexibility

We calculated the ECMs for the genome-scale *E*. *coli* model iJR904[39] by hiding all external metabolites except for glucose, oxygen, and biomass. This gives 12 ECMs that span all possible biomass yields on glucose and oxygen. The dots show, for the 10 ECMs that produce biomass, the necessary glucose and oxygen uptake to produce one unit biomass. The other two ECMs give the most extreme conversions from glucose and oxygen to non-biomass products, consuming only glucose (red arrow) or consuming the most oxygen per glucose (blue arrow). The convex combinations of biomass-producing ECMs combined with positive multiples of the non-biomass-producing ECMs give all feasible ways to produce one unit of biomass (yellow area).

genome-scale networks with thousands of reactions can still not be computed. We hope that this last scaling step can be made in the future. Even if this step cannot be made, the hide method described in Box 2 enables focusing on the most relevant set of external metabolites while the steady-state constraints are still satisfied in the whole network. In Figures 3 and 5 we illustrate with an *E*. *coli* core model and a genome-scale *H*. *pylori* model that this method can be used to obtain a much smaller set of conversions that spans all stoichiometric couplings between the user-defined external metabolites. This has not been possible with any other method. Moreover, when we focused only on the relations between glucose, oxygen, and biomass production, the hide method allowed us to scale ECM computation to the genome-scale *E*. *coli* model (Figure 6).

ECM enumeration ignores all information about the activities of reaction rates. If the hide method is used, even the consumption and production of the hidden metabolites is ignored. For example, if we hide everything but glucose, oxygen, and biomass, the ECMs show that the cell is capable of converting glucose and oxygen into biomass in the reported ratios. However, we obtain no information about which other metabolites can be consumed and produced during this conversion. Therefore, the ECMs obtained while hiding metabolites are generally not elementally balanced, illustrated by the question marks in Figure 4A. This might limit their use if one is, for example, interested in the thermodynamic properties of the conversion. However, for each ECM of interest, some flux distributions that lead to it can be reconstructed. These flux distributions can then be used to determine the overall conversion. The reconstruction could be done by imposing the conversion ratios from the ECM as equality constraints on the model. Solving an FBA problem would then give one candidate flux distribution, performing a flux variability analysis[49] would give the feasible ranges of all fluxes, and it might even be possible to find all elementary pathways that lead to this ECM by computing the elementary flux vectors.[34,50] In addition, if one is particularly interested in the activities of a certain set of reactions in the conversions, this can be reported by using the tag method, which is explained in detail in Box 2. In Figure 3C we used the tag method to highlight the use of the pyruvate dehydrogenase reaction in the e_coli_core network.

Conclusion

In this work we presented `ecmtool`, a computational tool that calculates all overall chemical conversions that a cell might catalyze—all of its metabolic capabilities—from its metabolic network alone. We hope that ECM enumeration will in the future become a standard step after metabolic network reconstruction so that the metabolism of all known organisms will be fully characterized.

EXPERIMENTAL PROCEDURES

Resource Availability

Lead Contact

Daan de Groot is the lead contact for this study and can be contacted by email at daanhugodegroot@gmail.com.

CellPress
OPEN ACCESS

Box 3. A Worked-Out Example of ECM Enumeration with the Direct Method

To show how ECM enumeration works in practice, we will here work out some steps of the computation of ECMs for the network given by the following stoichiometry matrix:

$$N = \begin{array}{c} \\ \underline{A} \\ \underline{E} \\ \underline{F} \\ \underline{BM} \\ B \\ C \\ D \\ G \end{array} \begin{array}{c} \begin{array}{ccccccc} v_1 & v_2 & v_3 & v_4 & v_5 & v_6 & v_7 \end{array} \\ \left[\begin{array}{ccccccc} -1 & -1 & 0 & 0 & 0 & 0 & 0 \\ 0 & 0 & 0 & 0 & -1 & 0 & 0 \\ 0 & 0 & 0 & 0 & 0 & -1 & 0 \\ 0 & 0 & 0 & 0 & 0 & 0 & 1 \\ 1 & 0 & -1 & 0 & -1 & -1 & 0 \\ 0 & 1 & 0 & -1 & 0 & 0 & 0 \\ 0 & 0 & 1 & 1 & 0 & 0 & -1 \\ 0 & 0 & 0 & 0 & 1 & 1 & -1 \end{array}\right] \end{array}, \qquad \text{(Equation 7)}$$

which is also shown as the first network of Figure 7A. All reactions are assumed irreversible, external metabolites A, E, and F can only be used as inputs, and BM can only be used as an output. For the enumeration we will use the direct intersection method, and we will not apply any of the network compression steps (examples of these steps can be found in Supplemental Information Section 5).

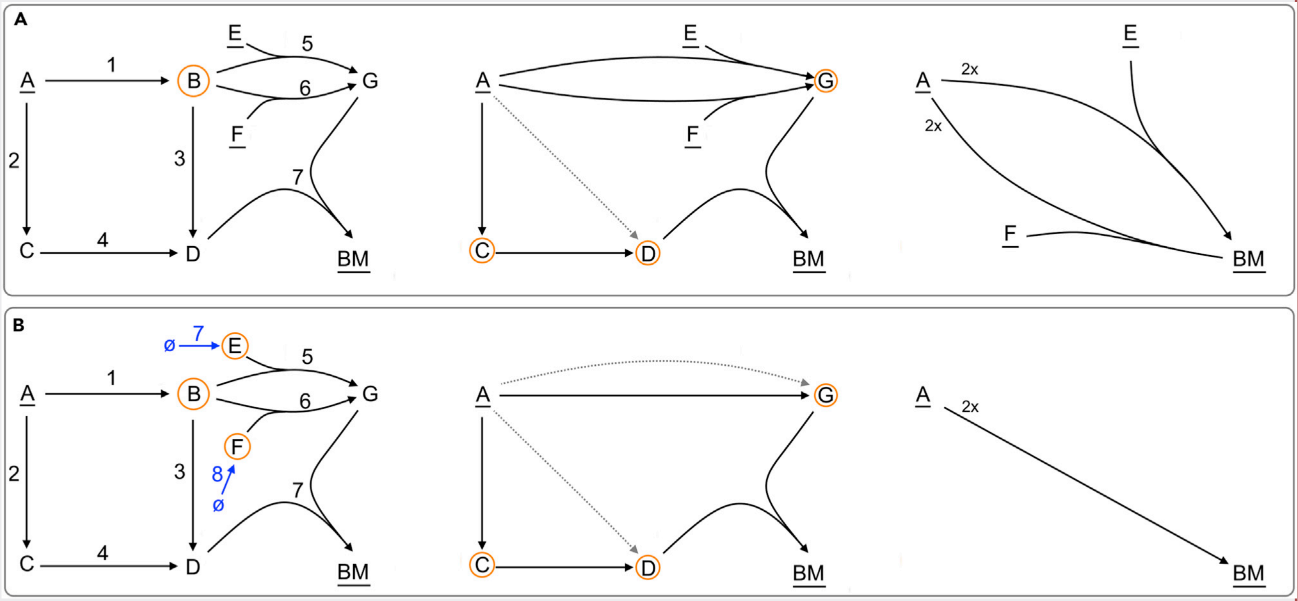

Figure 7. A Worked-Out Example of ECM Enumeration on a Small Network

Figure360▷ For a Figure360 author presentation of this figure, see https://doi.org/10.1016/j.patter.2020.100177.

All steps are described in the main text. Metabolites that are underlined are marked as external. The metabolites for which we impose the steady-state constraint in the next step are circled. Dotted arrows indicate conversions that were found to be redundant, and are thus deleted. (A) The network has two Elementary Conversions: using either E or F to convert A into BM. (B) When we ignore the uptake of E and F by using the hide-method, the metabolic capabilities is summarized by just one ECM, converting A into BM.

The stoichiometry matrix gives a list of generators that generates all conversions before we have imposed the steady-state constraints: $R^{(0)} = N$. On this collection of generators, we impose the steady-state constraint for metabolite B, i.e., $\dot{B} = 0$. In the stoichiometry matrix we can see that there are three reactions, v_2, v_4, v_7, that do not produce or consume B and therefore already satisfy this constraint. Of the other reactions, v_1 produces B and v_3, v_5, v_6 consume B. Each pair of a producing and a consuming reaction generates a candidate that satisfies the steady-state constraint, so this gives us $1 \times 3 = 3$ candidates:

- $v_1 + v_5$: $\underline{A} + \underline{E} \rightarrow G$;
- $v_1 + v_6$: $\underline{A} + \underline{F} \rightarrow G$;
- $v_1 + v_3$: $\underline{A} \rightarrow D$.

(Continued on next page)

Box 3. *Continued*

All candidates are tested for redundancy by the adjacency test described in Supplemental Information Section 8.1. This test indicates whether the candidate can be written as a positive combination of already existing reactions. The first two reactions are non-redundant and are thus added to the next list of generators, but the third reaction can be written as a sum of v_2 and v_4, and is therefore not added. We obtain

$$R^{(1)} = \begin{array}{c} \\ \underline{A} \\ \underline{E} \\ \underline{F} \\ \underline{BM} \\ C \\ D \\ G \end{array} \begin{bmatrix} v_2 & v_4 & v_1+v_5 & v_1+v_6 & v_7 \\ -1 & 0 & -1 & -1 & 0 \\ 0 & 0 & -1 & 0 & 0 \\ 0 & 0 & 0 & -1 & 0 \\ 0 & 0 & 0 & 0 & 1 \\ 1 & -1 & 0 & 0 & 0 \\ 0 & 1 & 0 & 0 & -1 \\ 0 & 0 & 1 & 1 & -1 \end{bmatrix}, \qquad \text{(Equation 8)}$$

which is depicted as the second network in Figure 7A.
This process is then repeated for internal metabolites C, D, and G, eventually giving

$$R^{(1)} = \begin{array}{c} \underline{A} \\ \underline{E} \\ \underline{F} \\ \underline{BM} \end{array} \begin{bmatrix} -2 & -2 \\ -1 & 0 \\ 0 & -1 \\ 1 & 1 \end{bmatrix}, \qquad \text{(Equation 9)}$$

containing all ECMs, namely $2\underline{A} + \underline{E} \rightarrow \underline{BM}$, and $2\underline{A} + \underline{F} \rightarrow \underline{BM}$.
In Figure 7B we illustrate the ECM enumeration when we use the hide method to ignore the consumption of E and F. Hiding these metabolites is done by extending the metabolic network with reactions that create E and F from nothing, and marking the metabolites as internal. We thus get

$$N = \begin{array}{c} \\ \underline{A} \\ \underline{E} \\ \underline{F} \\ \underline{BM} \\ B \\ C \\ D \\ G \end{array} \begin{bmatrix} v_1 & v_2 & v_3 & v_4 & v_5 & v_6 & v_7 & v_8 & v_9 \\ -1 & -1 & 0 & 0 & 0 & 0 & 0 & 0 & 0 \\ 0 & 0 & 0 & 0 & -1 & 0 & 0 & 1 & 0 \\ 0 & 0 & 0 & 0 & 0 & -1 & 0 & 0 & 1 \\ 0 & 0 & 0 & 0 & 0 & 0 & 1 & 0 & 0 \\ 1 & 0 & -1 & 0 & -1 & -1 & 0 & 0 & 0 \\ 0 & 1 & 0 & -1 & 0 & 0 & 0 & 0 & 0 \\ 0 & 0 & 1 & 1 & 0 & 0 & -1 & 0 & 0 \\ 0 & 0 & 0 & 0 & 1 & 1 & -1 & 0 & 0 \end{bmatrix}. \qquad \text{(Equation 10)}$$

When we now start by imposing the steady-state constraints for metabolite E, we see that only v_5 and v_8 do not satisfy this constraint. Combining these reactions gives the candidate $v_5 + v_8$: $B \rightarrow G$, which is added to the new list of generators. When we then impose the steady-state constraint for metabolite F, we get the same candidate $v_6 + v_9$: $B \rightarrow G$, but since this is not a new conversion it is not added to the list of generators. It can thus be seen that hiding metabolites E and F immediately reduces the computational complexity, because now only one reaction from B to G remains while without the hide method there were two such reactions. Moreover, after imposing the remaining steady-state constraints, we find only one ECM: $2\underline{A} + ?? \rightarrow \underline{G}$, where the question marks indicate that we do not know whether more metabolites are consumed because this information is hidden.
Although it would not give problems in this example, we can in general not hide a metabolite by simply removing it from the network. This is because information about whether the metabolite can be used as an input, as an output, or both would be lost from the computation. With the current method, this information is stored in the directionality of the added reaction.

Materials Availability

There are no physical materials associated with this study.

Data and Code Availability

The source code for `ecmtool` is freely available on GitHub at https://github.com/SystemsBioinformatics/ecmtool, and can additionally be installed through the Python package manager pip. A manual is available as Section 11 of Supplemental Information, which includes the commands for some worked-out examples for which the results are available in the GitHub repository.

This study did not generate any new datasets.

Methods

Here, we will describe only the most important conceptual steps of the ECMs computation. The method that was implemented in `ecmtool` is more elaborately described and explained in Supplemental Information. In developing this method we strongly benefited from the pioneering work by Urbanczik and Wagner, who not only defined ECMs but also described many of the enumeration steps. Unfortunately their enumeration tool, implemented in a mixture of Mathematica, MATLAB, and C, no longer functions, but many of the ideas can still be used. In the following, we will mention which conceptual steps were based on ideas from Urbanczik and Wagner and which were added by us.

The Minimal Ingredients for Computing ECMs

To start the computation of ECMs, we need the following ingredients:

1. A stoichiometry matrix
2. Reversibility information of all reactions
3. Information on which metabolites are external or internal
4. Information on whether external metabolites can be produced, consumed, or both

Our Python implementation can automatically extract these from an SBML (Systems Biology Markup Language[36]) file. In the case that it is not clear whether a reaction is reversible or not, the reaction can be assumed to be reversible. Incorrectly marking a reaction as reversible can only lead to some "false positives"—computed ECMs that are in fact not possible—but not to "false negatives." Since marking a metabolite as internal or external is sometimes ambiguous and context dependent, we here use our own definition: a metabolite is internal whenever the steady-state assumption should be met, so that its production and consumption should balance out.

Splitting External Metabolites into Inputs and Outputs Enables ECM Computation by Extreme Ray Enumeration

The ECMs, formally defined in Box 1, can be described as the elementary vectors[32] in the space of all steady-state conversions. This space is given by

$$\mathcal{C} = \left\{ \dot{\boldsymbol{c}} = N\boldsymbol{v} \middle| \dot{c}_i = 0 \text{ if } i \in Int,\ v_j \geq 0 \text{ for all } j \right\}, \quad \text{(Equation 2)}$$

where N is the stoichiometry matrix and Int the index set of internal metabolites. We have, for simplicity, assumed all reactions to be irreversible, but this is not necessary.

We first consider the case that the space of steady-state conversions is contained in one orthant, i.e., that for each dimension, i, all conversions are either non-negative ($\dot{c}_i \geq 0$) or non-positive ($\dot{c}_i \leq 0$). In that case, the elementary vectors coincide with the spanning rays: a well-defined minimal set of vectors with which we can generate the cone by taking conical combinations (weighted sums with positive weights). Enumerating the extreme rays of a polyhedral cone is a known mathematical problem described, for example, by Fukuda.[51] However, the set $\mathcal{C}$ is generally not contained in one orthant, because some external metabolites can be used as an input ($\dot{c}_i<0$) in some conversions, and as an output ($\dot{c}_i>0$) in others. This adds ECMs that are not spanning rays of $\mathcal{C}$, so that extreme ray enumeration is no longer enough.

We devised a new method to solve this problem: we extend the network slightly to make a new $\mathcal{C}$ that is contained in one orthant. Let A_{ex} be an external metabolite that is both an input and an output. We connect A_{ex} to two virtual metabolites, $A_{ex,in}$ and $A_{ex,out}$, through two irreversible reactions: $A_{ex,in} \rightarrow A_{ex}$ and $A_{ex} \rightarrow A_{ex,out}$. Finally, we mark A_{ex} itself as an internal metabolite, such that it has to be kept in steady state. As a consequence, conversions in which A_{ex} was produced must now produce $A_{ex,out}$ to maintain the steady-state assumption. Likewise, conversions in which A_{ex} was consumed must now consume $A_{ex,in}$. As such, all information about A_{ex} is stored in the production of $A_{ex,out}$ and the consumption of $A_{ex,in}$, while these new external metabolites can only be produced or consumed. Therefore, the new space of steady-state conversions is contained in a single orthant, so that we can proceed with the ECM computation by enumerating the spanning rays of this space. After the calculation we can then undo the splitting of metabolites so that we obtain the full set of ECMs (we prove this in Supplemental Information Section 3.3).

Finding the ECMs Is Finding a Generator Representation of $\mathcal{C}$

The space of steady-state conversions $\mathcal{C}$ is a so-called pointed polyhedral cone. Such a cone can be described in two ways: with an inequality representation or with a generator representation.[52]

The inequality representation is a set of vectors $\{\boldsymbol{a}_1, \ldots, \boldsymbol{a}_M\}$ that give the bounds that constrain the cone. All elements in the cone $\dot{\boldsymbol{c}} \in \mathcal{C}$ must satisfy $\boldsymbol{a}_i \cdot \dot{\boldsymbol{c}} \geq 0$ for all i. Or, as a matrix equation,

$$\mathcal{C} = \left\{ \dot{\boldsymbol{c}} \in \mathbb{R}^n \middle| A\dot{\boldsymbol{c}} \geq 0,\ A = \begin{bmatrix} \boldsymbol{a}_1^T \\ \vdots \\ \boldsymbol{a}_M^T \end{bmatrix} \right\} \quad \text{(inequality representation).}$$

(Equation 3)

In the generator representation one gives a set of vectors, $\{\boldsymbol{r}_1, \ldots \boldsymbol{r}_K\}$, with which all elements in the cone can be generated by taking conical combinations:

$$\mathcal{C} = \{\dot{\boldsymbol{c}} = R\lambda \mid \lambda_i \geq 0,\ R = [\boldsymbol{r}_1 \quad \ldots \quad \boldsymbol{r}_K]\} \quad \text{(generator representation).}$$

(Equation 4)

Since we have split the external metabolites into inputs and outputs before, computing the ECMs now amounts to obtaining a minimal generator representation of $\mathcal{C}$, because the generators, $\boldsymbol{r}_i$, are then precisely the ECMs.

The Main Computation Step: Impose Equality Constraints on a Large Set of Generators

Following Urbanczik and Wagner,[31] we will start the computation with a cone that is too large, but for which we already have a generator representation. To be precise, we will start with the cone generated by the columns of the stoichiometry matrix:

$$\mathcal{C}_0 = \{\dot{\boldsymbol{c}} = N\boldsymbol{\lambda} \mid \lambda_i \geq 0\}. \quad \text{(Equation 5)}$$

This cone is the space of all conversions that can result from combinations of reactions of the metabolic network, regardless of whether these conversions meet the steady-state requirement or not. Therefore, this cone does contain the steady-state conversion cone, $\mathcal{C}$, because it contains all possible conversions in steady state. However, to get a good description of $\mathcal{C}$ we should still impose the steady-state constraint. To compute the ECMs, we should therefore keep track of how our set of generators changes while we impose the steady-state equalities $\dot{c}_i = 0$ for each internal metabolite.

Concluding: we start with a set of generators of the cone $\mathcal{C}_0$, we impose the set of equalities given by $\dot{c}_i = 0$, and are then interested in the generators of the resulting cone. We have implemented two methods for this main part of the computation: an indirect method, which was extended from suggestions in literature,[31,51] and a direct method, which we developed ourselves. These methods are described elaborately in Supplemental Information Sections 7 and 8, but we will also briefly explain both below. We chose to implement both methods because their merits complement each other. The indirect method is fast on small- to medium-scale networks, and might therefore be preferred over the direct method. The method is called indirect because it first computes a large intermediate result, which is then used to compute the ECMs. However, the intermediate result might be much larger than the final result, so that the indirect method can run into memory issues while calculating the intermediate result, even though the final result is not that large. Our newly developed direct method performs better on larger networks, and especially when many metabolites are hidden using the hide method, because it avoids such large intermediate results.

The Indirect Method

As we will explain below, the indirect method twice uses the DD method.[40,53] The DD method computes a minimal set of generators from an inequality representation of a cone. This part of the computation is done using polco.[54]

Although our actual starting point is a generator representation of $\mathcal{C}_0$, it is useful for now to imagine that we already have an inequality representation of $\mathcal{C}_0$. We will later explain how we obtain this representation. This inequality representation would be a set of vectors $\boldsymbol{h}_1, \ldots \boldsymbol{h}_M$, such that

$$\mathcal{C}_0 = \left\{ \dot{\boldsymbol{c}} \in \mathbb{R}^n \middle| H\dot{\boldsymbol{c}} \geq 0,\ H = \begin{bmatrix} \boldsymbol{h}_1^T \\ \vdots \\ \boldsymbol{h}_M^T \end{bmatrix} \right\}. \quad \text{(Equation 6)}$$

Given this representation, it is easy to impose a steady-state constraint $\dot{c}_i = 0$, by adding the elementary unit vector $\hat{\boldsymbol{e}}_i = [0, \cdots, 0, 1, 0, \cdots, 0]^T$ both positively

and negatively to the set of inequalities. This enforces $0 \leq \hat{\boldsymbol{e}}_i \cdot \dot{\boldsymbol{c}} = \dot{c}_i$ and $0 \leq -\hat{\boldsymbol{e}}_i \cdot \dot{\boldsymbol{c}} = -\dot{c}_i$, such that we have actually imposed $\dot{c}_i = 0$. In our implementation, we have sped up the computation by imposing the steady-state constraint through removing the ith column from the inequality constraint matrix H. We prove in Supplemental Information Section 7.3.1 that this is equivalent. Removing these columns can make many of the rows in the constraint matrix redundant. For this, we have developed a redundancy removal algorithm that minimizes the size of the inequality constraint matrix (see Supplemental Information Sections 5.6 and 7.3.3).

Comparing Equations 2 and 6, we see that by imposing these steady-state constraints for all internal metabolites we go from an inequality representation for $\mathcal{C}_0$ to an inequality representation of the cone of steady-state conversions $\mathcal{C}$. From this, we can use the DD method to compute a minimal set of generators for this cone, yielding the ECMs.

It remains to be shown how we obtain an inequality representation of $\mathcal{C}_0$ from the generator representation we start with. For this, we assume that $\mathcal{C}_0$ has a dual cone associated with it: $\mathcal{C}_0^*$, which has two important properties (see Supplemental Information Section 1.3 for more information and explanation):

(1) the dual of the dual cone is again the cone: $(\mathcal{C}_0^*)^* = \mathcal{C}_0$;
(2) the vectors in the generator representation of a cone form an inequality representation of the dual cone, and vice versa: $\text{gen}(\mathcal{C}_0) = \text{ineq}(\mathcal{C}_0^*)$.

Our computation starts from a generator representation of $\mathcal{C}_0$ (Equation 5), but by property 2 this is also an inequality representation of its dual $\mathcal{C}_0^*$. By applying the DD method on this inequality representation, we can find a generator representation of $\mathcal{C}_0^*$. This generator representation is, again by property 2, an inequality representation of the dual of this dual cone. By property 1, we have thus obtained an inequality representation of $\mathcal{C}_0$, which was exactly what we needed. The steady-state constraints can now be imposed and the ECMs computed, as explained above.

Note that this indirect method heavily relies on the DD method. We found that polco[54] functions well and is reasonably fast, but can run into memory issues when the networks for which we try to compute the ECMs get too large. We found that these memory issues were caused by the size of the inequality representation needed to describe $\mathcal{C}_0$, i.e., the issues arise in the first application of the DD method. This therefore causes a computational limitation even though the generator representation of $\mathcal{C}$ (which we are eventually after) can be much smaller. This lack of control of the size of our intermediate results forms an important disadvantage of the indirect method. Therefore, we developed the direct method for the computation of ECMs for larger networks.

The Direct Method

Just as the indirect method, the direct method starts with the cone $\mathcal{C}_0$ introduced in Equation 5, generated by the columns of the stoichiometry matrix N. We collect these generators in a matrix $R^{(0)}$. We then iteratively impose the steady-state constraints, $\dot{c}_i = 0$, for all internal metabolites i. Imposing such a steady-state constraint means that we take the intersection of the cone $\mathcal{C}_0$ with the hyperplane $\dot{c}_i = 0$. The intersection is again a cone, called $\mathcal{C}^{(1)}$, which is generated by a new set of generators that we collect in a matrix $R^{(1)}$. Proceeding with $R^{(1)}$ and imposing more steady-state constraints, we will eventually end up with a set of generators for the steady-state conversion cone $\mathcal{C}$.

One such iteration thus starts with a set of generators of $C^{(i-1)}$, collected in $R^{(i-1)}$. Now, we distribute these generators into three groups—a plus-group, a zero-group, and a minus-group—depending on whether the generators have $\dot{c}_i > 0$, $\dot{c}_i = 0$, or $\dot{c}_i < 0$, respectively. The generators in the plus- and minus-groups do not satisfy the steady-state constraint, and should therefore be dropped. However, each combination of a plus-generator with a minus-generator can provide a candidate generator that does satisfy $\dot{c}_i = 0$. These candidates, combined with the generators that were already in the zero-group, must contain all generators of $\mathcal{C}^{(i)}$.

However, when we combine all generators from the plus-group with the minus-group to create new generators, we will not get a *minimal* set of generators. In other words, the cone $\mathcal{C}^{(i)}$ could also be generated by a smaller number of generators. This might not seem like a large problem, but the number of unnecessary generators (also called redundant generators) grows exponentially with the number of iterations, quickly causing computational infeasibility. Therefore, we have developed an adjacency test. This test determines for each candidate, i.e., an appropriate combination of a plus-generator with a minus-generator, whether it is redundant. It does so by checking if the candidate can be written as a combination of other generators. If so, the candidate is redundant and should be left out of $R^{(i)}$. This test is implemented by performing a linear optimization for each candidate. In Supplemental Information Section 8.1 we have added figures to explain our method, and also elaborate on the linear optimization and explain how we optimized it to be fast enough.

Although performing many linear optimizations is in principle a very slow process, there is an important advantage: the different optimizations can be done completely independently. Therefore, we were able to parallelize this direct method so that it can now be run on large computation clusters.

Network Compression Facilitates ECM Computation on Large Networks

ECM theory focuses on the overall conversions between external metabolites instead of on how these conversions come about internally. This distinction can be exploited to simplify the network even before we start the main computation steps described above. We have implemented several compression steps that together bring large networks back to a workable size. Most of these compression steps were suggested by Urbanczik and Wagner.[31] We have added the removal of cycles, the removal of redundant reactions, and part of the removal of infeasible reactions. In Supplemental Information Section 5 we provide proofs and more extensive explanations.

Infeasible Reactions Can Be Removed. The flux vectors that give rise to the ECMs should satisfy the steady-state and the irreversibility constraints. If we can prove that a reaction can never be active in a solution that meets both of these constraints, this reaction can be safely removed. In principle, the feasibility of a reaction can be tested by running a linear optimization: for reaction i we would maximize v_i such that $v_j \geq 0$ for irreversible reactions j, and such that $N_{int}\boldsymbol{v} = 0$, where N_{int} is the part of the stoichiometry matrix corresponding to internal metabolites. If the optimal solution does not give v_i strictly larger than zero, then reaction i is infeasible and can be removed from the network. In Section 5.1 of Supplemental Information we describe a computationally efficient way of achieving the same.

Redundant Reactions Can Be Deleted. We can delete redundant reactions; a reaction is called redundant if it can be written as a conical combination of other reactions. Because the function of these reactions can always be replaced by the combination of the other reactions, it does not add functionality to the network and can therefore be removed. Redundant reactions in systems with fewer than about 10,000 reactions can be removed using a program called redund from lrslib,[55] so that this suffices during this compression step. As we have mentioned above, we also apply redundancy removal during both the direct and the indirect method, and here the number of reactions can become much larger than 10,000. This is the reason why we also developed our own parallelizable redundancy test (see Supplemental Information Section 5.6 for an explanation).

Reversible Reactions Can Be Used to Cancel a Reaction and a Metabolite. Each reversible reaction can be used to cancel itself and one metabolite it connects to. Say that a reversible reaction, R_1, produces an internal metabolite A, and say that there are several other reactions producing or consuming A. We prove in Section 5.3 of Supplemental Information that we can, without changing the ECMs of the network, add or subtract reaction R_1 to these other reactions such that the production or consumption of A is canceled. After doing this for all reactions connected to A, R_1 is the only reaction left that produces A. This implies that no reaction flux is possible through R_1 in a steady-state solution, because the production of A cannot be compensated by another reaction. Therefore, we can delete both R_1 and A from the network without affecting the ECM results.

Dead-End Metabolites and Connecting Reactions Can Be Deleted. Sometimes an internal metabolite can only be produced and not consumed, or vice versa. In this case, the reaction flux through the reactions connected to this metabolite has to be zero in any steady-state solution. Therefore, we can delete the metabolite and all connecting reactions without affecting the set of ECMs.

Reactions with a Unique Function Can Be Used to Cancel a Reaction and a Metabolite. Say that we have a reaction R_1 which is the sole reaction that produces a metabolite A, but that there are several reactions that consume A. Then, again without affecting the set of ECMs, we can add R_1 to these consuming reactions such that the consumption of A is exactly canceled. The reaction R_1 is now the only reaction left that produces A, and can therefore not be active in a steady-state solution. We can thus cancel both R_1 and A.

CellPress
OPEN ACCESS

Cycles of k *Reactions Can Cancel* $\mathbf{k}-\mathbf{1}$ *Reactions and Metabolites*. A cycle is a set of reactions that can be used in a certain ratio such that nothing is produced or consumed. Say that the reactions $R_1, \ldots, R_k$ form a cycle, so that with appropriate weights λ_i we have $\lambda_1 R_1 + \cdots + \lambda_k R_k = \varnothing \rightarrow \varnothing$. In addition, say that R_1 produces an internal metabolite A. In Section 5.5 of Supplemental Information we show that we can now use a trick similar to what we used with the reversible reactions. We use $\lambda_1 R_1$ as the forward reaction and $\lambda_2 R_2 + \cdots + \lambda_k R_k$ as the backward reaction to cancel the production and consumption of A. After doing this, R_1 will again be the only reaction producing A, so that we can delete both R_1 and A from the network. Since we compensated for the action of R_1 in the rest of the network, we will be left with a cycle using the reactions $R_2, \ldots, R_k$, on which we can use the same trick again. In this way, we can delete $k-1$ reactions and $k-1$ metabolites.

The ECM Computation Was Implemented in Python

We implemented our algorithms in a publicly available Python program called `ecmtool`. It is freely available on GitHub at https://github.com/SystemsBioinformatics/ecmtool, and can additionally be installed through the Python package manager pip. The direct and indirect computation methods are both available within the program. A manual is available as Section 11 of Supplemental Information, and some worked-out examples are given.

SUPPLEMENTAL INFORMATION

Supplemental Information can be found online at https://doi.org/10.1016/j.patter.2020.100177.

ACKNOWLEDGMENTS

We thank Brett Olivier and Leen Stougie for helpful discussions and Carolin Schulte for showing us the scope of the potential applications of ecmtool. B.T. and D.H.d.G. were supported by NWO VICI grant 865.14.005 (https://www.nwo.nl/).

AUTHOR CONTRIBUTIONS

Conceptualization, D.H.d.G. and T.J.C.; Software, T.J.C., E.B.B., and D.H.d.G.; Validation, T.J.C., E.B.B., and D.H.d.G.; Formal Analysis, T.J.C., E.B.B., R.P., and D.H.d.G.; Resources, F.J.B. and B.T.; Writing – Original Draft, D.H.d.G.; Writing – Review & Editing, T.J.C., R.P., F.J.B., B.T., and D.H.d.G.; Visualization, T.J.C., E.B.B., and D.H.d.G; Supervision, R.P., F.J.B., B.T., and D.H.d.G; Funding Acquisition, F.J.B. and B.T.

DECLARATION OF INTERESTS

The authors declare no competing interests.

Received: August 4, 2020
Revised: October 7, 2020
Accepted: December 4, 2020
Published: December 29, 2020

REFERENCES

1. Roels, J.A. (1980). Application of macroscopic principles to microbial metabolism. Biotechnol. Bioeng. *22*, 2457–2514.
2. Happel, J., and Sellers, P.H. (1989). The characterization of complex systems of chemical reactions. Chem. Eng. Commun. *83*, 221–240.
3. de Hollander, J.A. (1991). The use of stoichiometric relations for the description and analysis of microbial cultures. Antonie Van Leeuwenhoek *60*, 257–273.
4. von Stockar, U., Gustafsson, L., Larsson, C., Marison, I., Tissot, P., and Gnaiger, E. (1993). Thermodynamic considerations in constructing energy balances for cellular growth. Biochim. Biophys. Acta *1183*, 221–240.
5. Heijnen, S.J. (1994). Thermodynamics of microbial growth and its implications for process design. Trends Biotechnol. *12*, 483–492.
6. Von Stockar, U., Maskow, T., Liu, J., Marison, I.W., and Patiño, R. (2006). Thermodynamics of microbial growth and metabolism: an analysis of the current situation. J. Biotechnol. *121*, 517–533.
7. Saadat, N.P., Nies, T., Rousset, Y., and Ebenhö, O. (2020). Thermodynamic limits and optimality of microbial growth. Entropy (Basel) *22*, 277.
8. Von Stockar, U., and van der Wielen, L.A. (2013). Biothermodynamics: The Role of Thermodynamics in Biochemical Engineering (EFPL Press).
9. Thiele, I., and Palsson, B.Ø. (2010). A protocol for generating a high-quality genome-scale metabolic reconstruction. Nat. Protoc. *5*, 93–121.
10. Mendoza, S.N., Olivier, B.G., Molenaar, D., and Teusink, B. (2019). A systematic assessment of current genome-scale metabolic reconstruction tools. Genome Biol. *20*, 158.
11. Schuster, S., and Hilgetag, C. (1994). On Elementary Flux Modes in biochemical reaction systems at steady state. J. Biol. Syst. *2*, 165–182.
12. Terzer, M. (2009). Large Scale Methods to Enumerate Extreme Rays and Elementary Modes (ETH Zurich).
13. Gagneur, J., and Klamt, S. (2004). Computation of elementary modes: a unifying framework and the new binary approach. BMC Bioinformatics *5*, 175.
14. Schilling, C.H., Schuster, S., Palsson, B.O., and Heinrich, R. (1999). Metabolic pathway analysis: basic concepts and scientific applications in the post-genomic era. Biotechnol. Prog. *15*, 296–303.
15. Hunt, K.A., Folsom, J.P., Taffs, R.L., and Carlson, R.P. (2014). Complete enumeration of Elementary Flux Modes through scalable demand-based subnetwork definition. Bioinformatics *30*, 1569–1578.
16. van Klinken, J.B., and Willems van Dijk, K. (2016). FluxModeCalculator: an efficient tool for large-scale flux mode computation. Bioinformatics *32*, 1265–1266.
17. Klamt, S., and Stelling, J. (2002). Combinatorial complexity of pathway analysis in metabolic networks. Mol. Biol. Rep. *29*, 233–236.
18. Jol, S.J., Kümmel, A., Terzer, M., Stelling, J., and Heinemann, M. (2012). System-level insights into yeast metabolism by thermodynamic analysis of Elementary Flux Modes. PLoS Comput. Biol. *8*, 1–9.
19. Gerstl, M.P., Jungreuthmayer, C., and Zanghellini, J. (2015). tEFMA: computing thermodynamically feasible Elementary Flux Modes in metabolic networks. Bioinformatics *31*, 2232–2234.
20. Peres, S., Jolicœur, M., Moulin, C., Dague, P., and Schuster, S. (2017). How important is thermodynamics for identifying Elementary Flux Modes? PLoS One *12*, e0171440.
21. David, L., and Bockmayr, A. (2014). Computing Elementary Flux Modes involving a set of target reactions. IEEE/ACM Trans. Comput. Biol. Bioinform. *11*, 1099–1107.
22. Kelk, S.M., Olivier, B.G., Stougie, L., and Bruggeman, F.J. (2012). Optimal flux spaces of genome-scale stoichiometric models are determined by a few subnetworks. Sci. Rep. *2*, 580.
23. Chan, S.H.J., and Ji, P. (2011). Decomposing flux distributions into Elementary Flux Modes in genome-scale metabolic networks. Bioinformatics *27*, 2256–2262.
24. Pey, J., and Planes, F.J. (2014). Direct calculation of Elementary Flux Modes satisfying several biological constraints in genome-scale metabolic networks. Bioinformatics *30*, 2197–2203.
25. de Figueiredo, L.F., Podhorski, A., Rubio, A., Kaleta, C., Beasley, J.E., Schuster, S., and Planes, S.J. (2009). Computing the shortest Elementary Flux Modes in genome-scale metabolic networks. Bioinformatics *25*, 3158–3165.
26. Machado, D., Soons, Z., Patil, K.R., Ferreira, E.C., and Rocha, I. (2012). Random sampling of Elementary Flux Modes in large-scale metabolic networks. Bioinformatics *28*, i515–i521.
27. Marashi, S.A.A., David, L., and Bockmayr, A. (2012). Analysis of metabolic subnetworks by flux cone projection. Algorithms Mol. Biol. *7*, 17.
28. Zanghellini, J., Gerstl, M.P., Hanscho, M., Nair, G., Regensburger, G., Müller, S., Jungreuthmayer, C., et al. (2017). Toward genome-scale

metabolic pathway analysis. In Industrial Biotechnology: Microorganisms, *Vol. 1*, C. Wittmann and J.C. Liao, eds. *(Wiley-VCH Verlag GmbH & Co. KGaA), pp. 111–123.*

29. Röhl, A., and Bockmayr, A. (2019). Finding MEMo: minimum sets of elementary flux modes. J. Math. Biol. *79*, 1749–1777.
30. Jungreuthmayer, C., Ruckerbauer, D.E., Gerstl, M.P., Hanscho, M., and Zanghellini, J. (2015). Avoiding the enumeration of infeasible Elementary Flux Modes by including transcriptional regulatory rules in the enumeration process saves computational costs. PLoS One *10*, e0129840.
31. Urbanczik, R., and Wagner, C. (2005). Functional stoichiometric analysis of metabolic networks. Bioinformatics *21*, 4176–4180.
32. Rockafellar, R.T.. (1969). The elementary vectors of a subspace of. In Combinatorial Mathematics and its Applications (UNC Press), pp. 104–127.
33. Müller, S., and Regensburger, G. (2016). Elementary vectors and conformal sums in polyhedral geometry and their relevance for metabolic pathway analysis. Front. Genet. *7*, 90.
34. Klamt, S., Regensburger, G., Gerstl, M.P., Jungreuthmayer, C., Schuster, S., Mahadevan, R., Zanghellini, J., et al. (2017). From elementary flux modes to elementary flux vectors: metabolic pathway analysis with arbitrary linear flux constraints. PLoS Comput. Biol. *13*, e1005409.
35. Stucki, J.W., and Urbanczik, R. (2005). Pyruvate metabolism in rat liver mitochondria. FEBS J. *272*, 6244–6253.
36. Hucka, M., Finney, A., Sauro, H.M., Bolouri, H., Doyle, J.C., Kitano, H., Arkin, A.P., Bornstein, B.J., Bray, D., Cornish-Bowden, A., et al. (2003). The systems biology markup language (SBML): a medium for representation and exchange of biochemical network models. Bioinformatics *19*, 524–531.
37. Orth, J.D., Fleming, R.M.T., and Palsson, B.O. (2010). Reconstruction and use of microbial metabolic networks: the core *Escherichia coli* metabolic model as an educational guide. EcoSal Plus *4*, https://doi.org/10.1128/ecosalplus.10.2.1.
38. Thiele, I., Vo, T.D., Price, N.D., and Palsson, B. (2005). Expanded metabolic reconstruction of Helicobacter pylori (iIT341 GSM/GPR): an in silico genome-scale characterization of single- and double-deletion mutants. J. Bacteriol. *187*, 5818–5830.
39. Reed, J.L., Vo, T.D., Schilling, C.H., and Palsson, B.O. (2003). An expanded genome-scale model of Escherichia coli K-12 (iJR904 GSM/GPR). Genome Biol. *4*, R54.
40. Fukuda, K., and Prodon, A. (1996). Double description method revisited. In Combinatorics and Computer Science (CCS '95).Lecture Notes in Computing Science, *vol. 1120*, M. Deza, R. Euler, and I. Manoussakis, eds. *(Springer), pp. 91–111.*
41. Carlson, R., and Srienc, F. (2004). Fundamental *Escherichia coli* biochemical pathways for biomass and energy production: identification of reactions. Biotechnol. Bioeng. *85*, 1–19.
42. Poole, P., Ramachandran, V., and Terpolilli, J. (2018). Rhizobia: from saprophytes to endosymbionts. Nat. Rev. Microbiol. *16*, 291–303.
43. Lodwig, E., and Poole, P. (2003). Metabolism of rhizobium bacteroids. Crit. Rev. Plant Sci. *22*, 37–78.
44. Orth, J.D., Thiele, I., and Palsson, B.Ø. (2010). What is flux balance analysis? Nat. Biotechnol. *28*, 245.
45. Trinh, C.T., Wlaschin, A., and Srienc, F. (2009). Elementary mode analysis: a useful metabolic pathway analysis tool for characterizing cellular metabolism. Nat. Biotechnol. *81*, 813–826.
46. Clarke, B.L. (1988). Stoichiometric network analysis. Cell Biophys. *12*, 237–253.
47. Schilling, C.H., Letscher, D., and Palsson, B.Ø. (2000). Theory for the systemic definition of metabolic pathways and their use in interpreting metabolic function from a pathway-oriented perspective. J. Theor. Biol. *203*, 229–248.
48. Teusink, B., Wiersma, A., Jacobs, L., Notebaart, R.A., and Smid, E.J. (2009). Understanding the adaptive growth strategy of *Lactobacillus plantarum* by in silico optimisation. PLoS Comput. Biol. *5*, e1000410.
49. Mahadevan, R., and Schilling, C.H. (2003). The effects of alternate optimal solutions in constraint-based genome-scale metabolic models. Metab. Eng. *5*, 264–276.
50. Urbanczik, R. (2007). Enumerating constrained elementary flux vectors of metabolic networks. IET Syst. Biol. *1*, https://doi.org/10.1049/iet-syb:20060073.
51. Fukuda, K. (2004). Frequently asked questions in polyhedral computation. https://inf.ethz.ch/personal/fukudak/polyfaq/polyfaq.html.
52. Rockafellar, R.T. (1970). Convex Analysis (Princeton University Press).
53. Motzkin, T.S., Raiffa, H., Thompson, G.L., and Thrall, R.M. (1953). The double description method: Contributions to the theory of games. Ann. Math. Stud. *28*, 51–73.
54. Terzer, M., and Stelling, J. (2008). Large-scale computation of Elementary Flux Modes with bit pattern trees. Bioinformatics *24*, 2229–2235.
55. Avis, D. (2015). lrslib, version 6.0 http://cgm.cs.mcgill.ca/~avis/C/lrslib/USERGUIDE.html.

PATTER, Volume *2*

Supplemental Information

Unlocking Elementary Conversion Modes: ecmtool Unveils All Capabilities of Metabolic Networks

Tom J. Clement, Erik B. Baalhuis, Bas Teusink, Frank J. Bruggeman, Robert Planqué, and Daan H. de Groot

Contents

1 Background information on polyhedral computation 3
1.1 Inequality and generator descriptions of polyhedral cones 4
1.2 Pointedness 4
1.3 Dual cones 4
1.4 Adjacent rays 5
1.5 The double description method 5
1.5.1 Redundant rays 7

2 Defining Elementary Conversion Modes 7

3 Pre-processing of the metabolic networks 8
3.1 Deleting exchange reactions and determining directionality of external metabolites 8
3.2 Adding an objective metabolite 8
3.3 Splitting metabolites 8
3.4 Converting the stoichiometic coefficients into fractions 10
3.5 (Optional): Hiding metabolites and tagging reactions 10

4 A custom LP-solver 10
4.1 Problem description 10
4.2 Some background on Linear Programming 11
4.3 The revised simplex method 11
4.4 Pivoting: replacing one of the columns in the feasible basis 12
4.5 Finding a starting basis efficiently 13
4.6 Detecting an early exit possibility 14
4.7 Perturbation to remove degeneracy 14

5 Compression of the metabolic networks 15
5.1 Removal of infeasible reactions 15
5.2 Dead-end metabolites are deleted 15
5.3 Cancelling metabolites with a reversible reaction 15
5.4 Cancelling singly produced or consumed metabolites 16
5.5 Removing cycles by cancelling metabolites 17
5.5.1 Example 18
5.6 Removal of redundant reactions 18

6 The starting point: generator representation of the (non-steady-state) conversion cone 20

7 The indirect method 20
7.1 An inequality representation of the dual 20
7.1.1 An iterative symbolic nullspace calculation 21
7.2 The Double Description method gives a generator representation of the dual 21
7.3 An inequality representation of the conversion cone 21
7.3.1 Dropping columns corresponding to internal metabolites 21
7.3.2 Splitting external metabolites 22
7.3.3 Removing redundant inequalities 23
7.4 The Double Description method gives the ECMs 24

8 The direct method 24
8.1 Imposing one steady-state constraint 24
8.1.1 A geometric adjacency test 25
8.1.2 Performing the adjacency tests 28
8.2 The order of imposing steady-state constraints 28
8.3 Parallelization 29

9 Validation of `ecmtool` **29**

10 ECM-analyses several networks **31**
10.1 Creating subnetworks of `e_coli_core` . . . 32
10.2 Clustering the ECM results for visualisation . . . 32

11 User guide **32**
11.1 Prerequisite ingredients for ECM-computation . . . 32
11.2 Mode 1: standalone command line tool . . . 33
11.2.1 Optional arguments . . . 34
11.3 Mode 2: Python library . . . 35
11.4 Advanced usage . . . 36

Supplementary figures

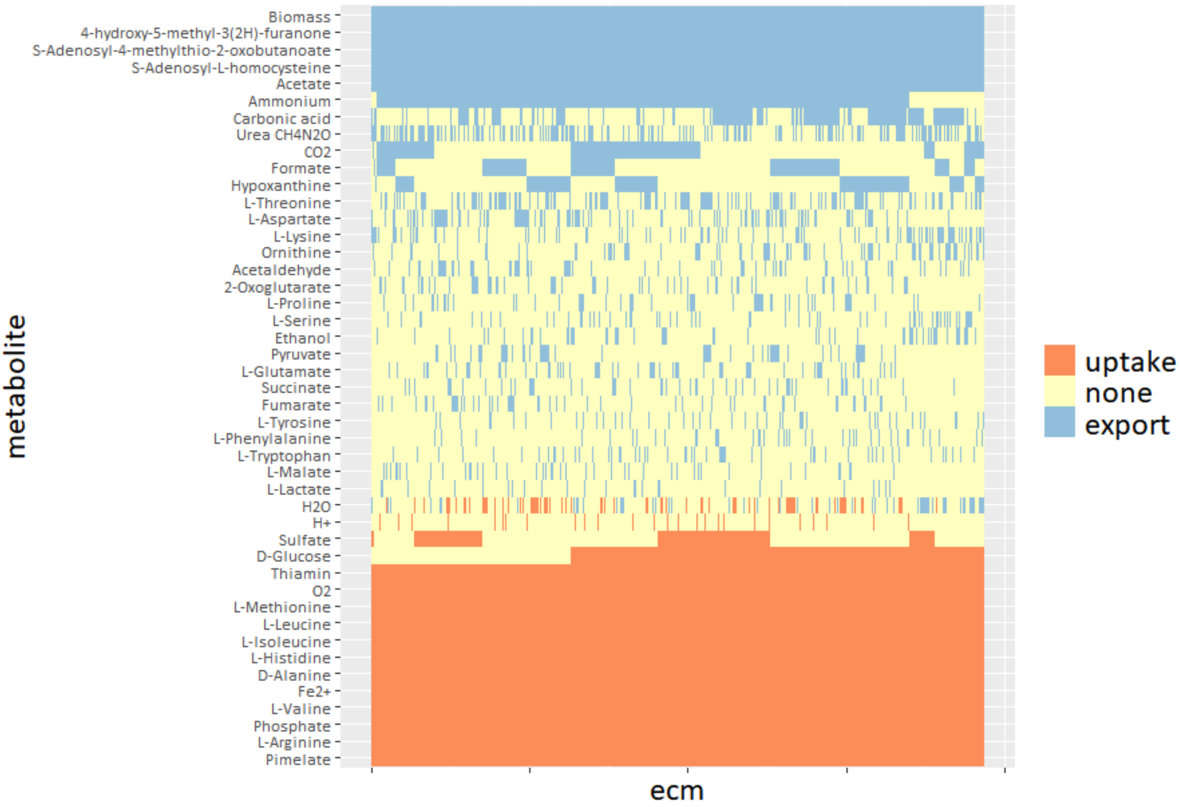

Figure S1: **All Elementary Conversion Modes for the `iIT_341`-model of *Helicobacter pylori* could be computed.** We show the ECMs that support growth on a defined medium proposed by the model developers (see minII in[1]). For visualization purposes, we only show which metabolites are consumed or produced rather than their exact stoichiometric coefficient. In addition, we show only the ECMs that in which L-alanine was not taken up, and in which acetate and biomass was produced, because the full set of 874 236 ECMs was simply too large to show. After these simplifications, there were still 7740 unique ECMs. The full set of ECMs is available as a supplementary file.

Notation and conventions

In the following, we will denote matrices by capital letters (A), and vectors by bold lowercase letters ($\boldsymbol{v}$) (or bold uppercase letters if the vectors are rows or columns from a matrix). The requirement that all components of a vector $\boldsymbol{v} \in \mathbb{R}^n$ are nonnegative, $v_i \geq 0,\ i \in \{1, ..., n\}$, will be written as $\boldsymbol{v} \geq \boldsymbol{0}$. Time derivatives are indicated by a dot, $\dot{c} = \frac{dc}{dt}$. All single vectors in this work denote column vectors, unless otherwise stated. The ith row of a matrix A is indicated as $\boldsymbol{A}_{i\bullet}$. Column vectors of A are sometimes denoted as $\boldsymbol{A}_{\bullet j}$.

In biochemical networks, an underlined metabolite ($\underline{\text{S}}$) is assumed to be ‘external’, which here means that the steady-state constraint does not have to be satisfied for that metabolite. To simplify the following analysis, we will assume all reactions to be irreversible, unless explicitly stated otherwise. We can do this without loss of generality since all reversible reactions can be decomposed into an irreversible forward and an irreversible backward reaction.

1 Background information on polyhedral computation

We will shortly review the concepts from linear algebra that we will need for the definition and enumeration of ECMs (see for example[2]).

1.1 Inequality and generator descriptions of polyhedral cones

A subset S of $\mathbb{R}^d$ is called a *convex cone* if any conical combination of two vectors $\boldsymbol{v}, \boldsymbol{w} \in S$ is still a vector in S, i.e., for all $\alpha, \beta \in \mathbb{R}_{\geq 0}$ we have $\alpha\boldsymbol{v} + \beta\boldsymbol{w} \in S$. S is called a *polyhedral convex cone* if there is some constraint matrix A such that

$$S = \left\{\boldsymbol{x} \in \mathbb{R}^d \mid A\boldsymbol{x} \geq \mathbf{0}\right\}, \qquad \text{(inequality representation)}. \tag{1}$$

This way of describing S is called the inequality-, or H-representation. The Minkowski-Weyl theorem tells us that every polyhedral cone can be generated by taking conical combinations of a finite set of vectors, $\boldsymbol{r_1}, \ldots, \boldsymbol{r_n} \in \mathbb{R}^d$. If we collect these vectors as the columns of a matrix R, this gives us a second representation of S called the generator-, or the V-representation:

$$S = \{\boldsymbol{x} = R\boldsymbol{\lambda} \mid \lambda_i \geq 0\}, \qquad \text{(generator representation)}. \tag{2}$$

Such representations of S are denoted by ineq(S) and gen(S), respectively.

1.2 Pointedness

Vectors $\boldsymbol{v} \in S$ for which $-\boldsymbol{v} \in S$ as well, are called linealities. The space that comprises all such vectors is called the lineality space. Since by the definition of S, we would have $A\boldsymbol{v} \geq \mathbf{0}$ and $A(-\boldsymbol{v}) = -A\boldsymbol{v} \geq \mathbf{0}$, we can describe the lineality space as the null-space of the constraint matrix that was used in the inequality representation of S, i.e.,

$$\mathrm{Lin}(S) = \left\{\boldsymbol{x} \in \mathbb{R}^d \mid A\boldsymbol{x} = \mathbf{0}\right\}. \tag{3}$$

A polyhedral cone is called *pointed* if its lineality space contains only the zero vector. A pointed cone has a unique generator representation.

In one of the steps of ECM-enumeration, one can encounter a non-pointed polyhedral cone. The computation, however, can only proceed with a pointed cone. In such cases we can use that any cone is the direct sum of its lineality space and a pointed cone.[3] To be precise, for any cone S, we have

$$S = \mathrm{Lin}(S) \oplus (S \cap \mathrm{Lin}(S)^{\perp}). \tag{4}$$

The pointed part of the polyhedral cone is thus given by all vectors in the cone that are perpendicular to the lineality space. Using a set of basis vectors $\boldsymbol{n}_1, \ldots, \boldsymbol{n}_k$ of the lineality space Lin(S), we can even get an inequality representations for the two parts

$$S = \left\{\boldsymbol{x} \in \mathbb{R}^d \mid A\boldsymbol{x} = \mathbf{0}\right\} \oplus \left\{\boldsymbol{x} \in \mathbb{R}^d \mid A\boldsymbol{x} \geq \mathbf{0}, \quad N\boldsymbol{x} = \mathbf{0}\right\}, \tag{5}$$

where $N = \begin{bmatrix}\boldsymbol{n}_1 & \cdots & \boldsymbol{n}_k\end{bmatrix}^T$.

1.3 Dual cones

Let S be a polyhedral cone in $\mathbb{R}^d$. Its *dual cone* S^* is defined as

$$S^* = \{\boldsymbol{u} \in \mathbb{R}^d \mid \boldsymbol{u} \cdot \boldsymbol{v} \geq 0, \boldsymbol{v} \in S\}. \tag{6}$$

The dual of the dual of a convex polyhedral cone is equal to itself if S is convex and closed.

In Figure S2 we illustrate an important property of dual cones: a generating set of C, gen(C), forms the inequalities representation of C^*, ineq(C^*), and vice versa:[4,5]

$$\mathrm{gen}(C) = \mathrm{ineq}(C^*), \tag{7}$$
$$\mathrm{ineq}(C) = \mathrm{gen}(C^*). \tag{8}$$

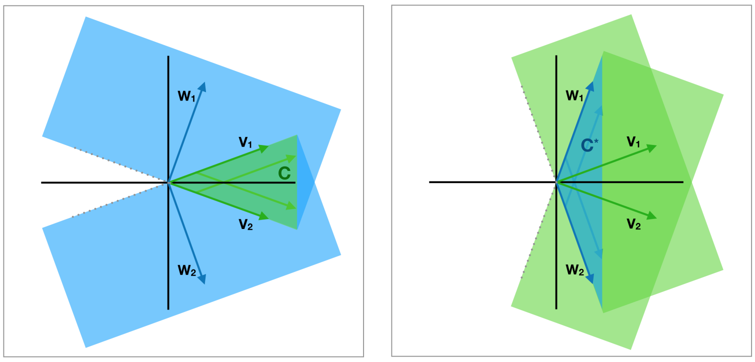

(a) (b)

Figure S2: **Cones are spanned by inequality constraints of their dual.** (a) A 2-dimensional cone $\mathcal{C}$ (green) spanned by generating vectors v_1 and v_2, meaning that any nonnegative linear combination of them (light-green arrows) is also in $\mathcal{C}$. $\mathcal{C}$ can additionally be described by the intersection of the two halfspaces $\boldsymbol{w}_1 \cdot \boldsymbol{x} \geq \mathbf{0}$ and $\boldsymbol{w}_2 \cdot \boldsymbol{x} \geq \mathbf{0}$ (blue). Here, $\boldsymbol{w}_1$ and $\boldsymbol{w}_2$ form the inequality representation of C. (b) The dual of $\mathcal{C}$, $\mathcal{C}^*$ (blue), is spanned by the inequality representation vectors of $\mathcal{C}$. Thus, we can see that the inequality representation of a cone forms generating vectors of its dual, and its generating vectors form the inequality representation of its dual. Both cones, $\mathcal{C}$ and $\mathcal{C}^*$, extend to infinity; only a section is drawn.

1.4 Adjacent rays

Assume that we have an $m \times n$ inequality representation A of the polyhedral cone

$$\mathcal{C} = \{\boldsymbol{x} \in \mathbb{R}^n : A\boldsymbol{x} \geq \mathbf{0}\}$$

and corresponding ray representation R:

$$\mathcal{C} = \{\boldsymbol{x} \in \mathbb{R}^n : \boldsymbol{x} = R\boldsymbol{\lambda}, \boldsymbol{\lambda} \geq \mathbf{0}\}.$$

For each extreme ray $\boldsymbol{r}_j$ of the cone (i.e., the j-th column of R), define the *zero set*

$$Z(\boldsymbol{r}_j) = \{i : \boldsymbol{A_{i\bullet}} \boldsymbol{r}_j = 0\}.$$

Two distinct extreme rays $\boldsymbol{r}_{j_+}, \boldsymbol{r}_{j_-} \in R$ are *adjacent* if for any extreme ray $\boldsymbol{r}_j$, we have

$$Z(\boldsymbol{r}_{j_+}) \cap Z(\boldsymbol{r}_{j_-}) \subseteq Z(\boldsymbol{r}_j) \implies \boldsymbol{r}_j \sim \boldsymbol{r}_{j_+} \text{ or } \boldsymbol{r}_j \sim \boldsymbol{r}_{j_-},$$

where $\sim$ means that the two vectors are scalar multiples of each other. In other words, two vectors $\boldsymbol{r}_{j_+}$ and $\boldsymbol{r}_{j_-}$ are adjacent if there are no other extreme rays that satisfy the constraints with equality that are satisfied with equality by both $\boldsymbol{r}_{j_+}$ and $\boldsymbol{r}_{j_-}$ from A.

Geometrically, two distinct extreme rays are adjacent if the minimal face of C containing both contains no other extreme rays. For more details, see Proposition 7 in.[6]

1.5 The double description method

The Double Description method is an algorithm originally suggested by Motzkin et al.[7] to translate between the two representations of a cone. For a more comprehensive description and proofs of the propositions, see.[6]

A pair (A, R) is called a **double description pair** or DD pair if A is an inequality representation and R is a generator representation of the same cone. That is,

$$\{\boldsymbol{x} \in \mathbb{R}^n : A\boldsymbol{x} \geq \mathbf{0}\} = \{\boldsymbol{x} \in \mathbb{R}^n : \boldsymbol{x} = R\boldsymbol{\lambda}, \boldsymbol{\lambda} \geq \mathbf{0}\}.$$

The following proposition shows that an algorithm that can translate in one direction automatically also solves the inverse direction.

Proposition 1. *(A, R) is a DD pair if and only if (R^T, A^T) is a DD pair.*

The double description method provides an algorithm to find an R based on a given A such that (A, R) is a DD pair. As we described in the previous section, R^T is an inequality representation for the dual cone of the cone represented by the inequalities in A. If we want to find a DD pair starting from a given R, we can take R^T and treat it as the inequality representation of a cone, then apply the double description method to find A^T (hence giving (A, R)).

The core of the double description algorithm is an incremental procedure. We start with an $m \times n$ matrix A, giving the inequality representation of a cone that we will denote by $P(A)$. Let $K \subset \{1, \ldots, m\}$ be a subset of the row indices of A and A_K the submatrix consisting of the corresponding rows. Suppose we already have a ray representation for the cone $P(A_K)$, i.e., we have a DD pair (A_K, R_K). To perform the incremental step, select any index i not in K; we will denote the corresponding row by $\boldsymbol{a}_i$. We will add $\boldsymbol{a}_i$ to A_K to form $A_{K\cup\{i\}}$ and use R_K to construct a DD pair $(A_{K\cup\{i\}}, R_{K\cup\{i\}})$.

First, we partition the column index set J of R_K into three parts:

$$J^+ = \{j \in J : \boldsymbol{a}_i R_{\bullet j} > 0\},$$
$$J^0 = \{j \in J : \boldsymbol{a}_i R_{\bullet j} = 0\},$$
$$J^- = \{j \in J : \boldsymbol{a}_i R_{\bullet j} < 0\}.$$

To make the matrix $R_{K\cup\{i\}}$ we keep all the columns $R_{\bullet j}$ from R_K with $j \in J^+$ or $j \in J^0$. These rays satisfy the inequality given by the row $\boldsymbol{a}_i$ already: $\boldsymbol{a}_i R_{\bullet j} \geq 0$ and so they are in $P(A_{K\cup\{i\}})$ unchanged. The columns corresponding to J^- are not in the new cone, but they give rise to new generating rays in the following way.

Let $\boldsymbol{r}_{jj'} = (\boldsymbol{a}_i R_{\bullet j})R_{\bullet j'} - (\boldsymbol{a}_i R_{\bullet j'})R_{\bullet j}$ for each $(j, j') \in J^+ \times J^-$. Note that $\boldsymbol{a}_i \boldsymbol{r}_{jj'} = 0$ for each choice of (j, j'). These columns are also added to $R_{K\cup\{i\}}$.

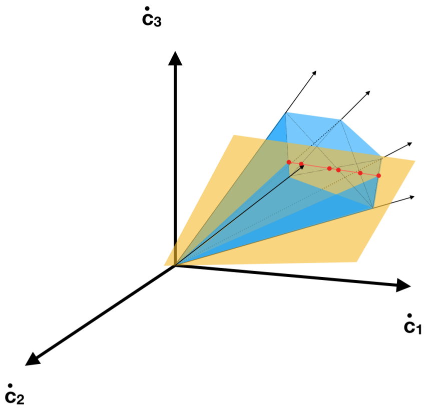

Figure S3: A cone (blue) spanned by generators (black) is being intersected with an inequality constraint (orange). All generators on one side of the constraint can remain, and those on the other side are replaced by the non-redundant the red vectors. The red vectors are conical combinations of each pair of extreme rays on both sides of the halfspace, such that the red vector exactly saturates the constraint. This process is repeated for all inequality constraints, until only the generating set of the final cone remains.

To sum up, $R_{K\cup\{i\}}$ is the $d \times |J'|$ matrix with columns given by the index set $J' = J^+ \cup J^0 \cup (J^+ \times J^-)$, where $\boldsymbol{r}_{jj'} = (\boldsymbol{a}_i R_{\bullet j})R_{\bullet j'} - (\boldsymbol{a}_i R_{\bullet j'})R_{\bullet j}$. In Figure S3, one intersection with an inequality constraint is illustrated.

Proposition 2. *$(A_{K\cup i}, R_{K\cup\{i\}})$ is a DD pair.*

Repeating this step, eventually all the rows of A will be included, giving a DD pair (A, R) as intended. What is left is to find is a starting point. A good option is starting with d linearly independent rows of A to form A_{K_0}. Then we can find an inverse matrix, and $A_{K_0}\boldsymbol{x} = \boldsymbol{\lambda} \geq \boldsymbol{0}$ implies that $\boldsymbol{x} = A_{K_0}^{-1}\boldsymbol{\lambda}$, so that $(A_{K_0}, A_{K_0}^{-1})$ is a DD pair.

1.5.1 Redundant rays

Although the Double Description method as described above gives a ray representation R, it is not necessarily a *minimal* representation: there can be redundant columns in R that are not extreme rays of the cone. In fact, the number of unnecessary rays in practice increases very fast and goes beyond any tractable limit.[6]

One possible solution is to discard all redundant vectors, preferably between each step of the Double Description method. This can be done, for example, with the program `redund` from `lrslib`,[8] which looks for redundant vectors by trying to find matching conic combinations of other rays (see also Section 5.6). However, this quickly becomes computationally intensive for large sets of vectors.

Alternatively, for each $\boldsymbol{r}_{jj'}$ that we created with $(j, j') \in J^+ \times J^-$, we can test the originating rays for adjacency, which we defined above in Section 1.4. It turns out the new ray $\boldsymbol{r}_{jj'}$ is redundant if and only if its parent rays were not adjacent (Lemma 8 in[6]). The Double Description method, including an adjacency test, is efficiently implemented in `Polco`.[9] In Section 8.1.2 we will return to this adjacency test, because we need an adapted version for our direct intersection method.

2 Defining Elementary Conversion Modes

A metabolic network with r reactions and m metabolites can be summarized in an $m \times r$ stoichiometry matrix N. The entries in this matrix denote how much of which metabolite (rows) are used in each reaction (columns). The stoichiometric matrix can be multiplied by a vector of reaction rates, a *flux vector* $\boldsymbol{v}$, to yield the net production and consumption of metabolites by this combination of reaction rates: $\dot{\boldsymbol{c}} = N\boldsymbol{v}$. We will call $\dot{\boldsymbol{c}}$ a *conversion.* We can assume without loss of generality that all reactions in the metabolic network are irreversible, meaning that $\boldsymbol{v} \geq \boldsymbol{0}$, and we will do so unless otherwise stated.

We often impose a steady-state constraint on a part of the metabolites, enforcing that the net production of these metabolites is zero. In the following, we will call the metabolites for which we impose the steady-state *internal metabolites*, and we collect their indices in an index set Int. We can now define two important sets, the *steady-state flux cone*:

$$\mathcal{F} = \{\boldsymbol{v} \in \mathbb{R}^r \mid N_{\text{Int}}\boldsymbol{v} = \boldsymbol{0},\ v_i \geq 0 \text{ if reaction } i \text{ irreversible}\}, \tag{9}$$

and the *steady-state conversion cone*:

$$\mathcal{C} = \{\dot{\boldsymbol{c}} = N\boldsymbol{v} \mid N_{\text{Int}}\boldsymbol{v} = \boldsymbol{0},\ v_i \geq 0 \text{ if reaction } i \text{ irreversible}\}. \tag{10}$$

Both of these sets are convex polyhedral cones and thus have both an inequality representation, and a generator representation. The definitions of Elementary Flux Modes and Elementary Conversion Modes are closely related to the generator representations of these cones. We here give a definition of EFMs that is different from their original definition, but which can be found in later literature.[10–12] We find that this definition clarifies the analogy with ECMs better.

Definition 1. *The set of* Elementary Flux Modes (EFMs) *is the minimal set* $\{efm_1, \dots, efm_K\} \subset \mathcal{F}$ *of flux vectors such that each steady-state flux vector can be written as a positive sum of EFMs, without the flux of any reaction being cancelled in that sum.*

The first part of this definition implies that all vectors in the generator description of the steady-state flux cone $\mathcal{F}$ are EFMs. However, in case that we have reversible reactions, these do not form the complete set of EFMs. Rather, we should add conical combinations of these generators that make an additional reaction rate equal to zero. The complete set of EFMs may be found by taking the union of all generator sets of the intersections of $\mathcal{F}$ with the orthants. In practice, it is easier to just split all reversible reactions in a forward and a backward reaction. As such, no reaction can be cancelled and the EFMs are exactly the generators of the new flux cone $\mathcal{F}$.

Definition 2. *The set of* Elementary Conversion Modes (ECMs) *is the minimal set of conversions* $\{ecm_1, \ldots, ecm_L\} \subset \mathcal{C}$ *such that each steady-state conversion can be written as a positive sum of ECMs, without the production of any external metabolite being cancelled in that sum.*

This definition is further explained and illustrated in Box 1 of the main text. It is important to note that a reasoning can be used here that is similar to what was used for the EFMs above. That is, there are two ways of calculating ECMs as the generators of a convex polyhedral cone. First, we can take the union of all generators of the cones obtained by intersecting $\mathcal{C}$ with the orthants. Second, we can split all metabolites that can be produced and consumed into two virtual metabolites: one that is consumed, and one that is produced. We will take the latter approach in this work.

3 Pre-processing of the metabolic networks

To facilitate the enumeration of ECMs, `ecmtool` goes through some pre-processing steps after reading in a metabolic network. We will here review these steps shortly. Although `ecmtool` works well with most models in the `SBML`-format, we recommend the user to always review the success of these preprocessing steps. We have added arguments called `--print_metabolites` and `--print_reactions` to facilitate this review. With the printed lists of parsed metabolites and reactions, the user can check if the correct metabolites are marked as external, and if their directionality is as intended.

3.1 Deleting exchange reactions and determining directionality of external metabolites

`Ecmtool` first detects external metabolites by using functionalities from the `cbmpy`-package (`http://cbmpy.sourceforge.net/`). Metabolites are marked external when their metabolite-id has a specific suffix (the suffix for the external compartment is set to `e`, but this can be changed with the argument `--external_compartment`), or when the metabolite is attached to a 'dead-end reaction', which is a reaction that involves only one metabolite.

These dead-end reactions are virtual reactions called exchange reactions. Exchange reactions are present in models to allow the steady-state assumption to hold even for the external metabolites, since these external metabolites are often assumed fixed. In the case of ECMs, however, we are interested in the production and consumption of these external metabolites, and will therefore delete all exchange reactions.

Based on the directionality, reversibility and constraints of the exchange reactions in the model, `ecmtool` will detect if the external metabolite can be used as an input, an output, or as both. This conclusion can be overruled easily by using the `--inputs`- and `--outputs`-arguments.

3.2 Adding an objective metabolite

Many models contain an objective reaction, often a virtual reaction that uses all components that are needed for biomass production in the right proportions. Although ECMs in principle do not report any reaction rates, the rate of the objective reaction is most often of interest. Therefore, `ecmtool` by default adds an external 'objective metabolite' to the model that is produced in the objective reaction. In this manner, the rate of the objective reaction is reported in the production of the objective metabolite. The addition of this objective metabolite can be disabled by the `--add_objective_metabolite`-argument.

3.3 Splitting metabolites

As was discussed in Section 2, not all ECMs are generating vectors of the steady-state conversion cone defined in (10), unless this cone is contained in one orthant. However, the conversion cone might not be contained in one orthant since some metabolites can both be consumed $\dot{c}_i < 0$ and produced $\dot{c}_i > 0$. In the Method-section of the main text, we explained how this can be remedied by splitting external metabolites into input- and output-metabolites. We will here make this explanation more precise.

We start with a stoichiometric matrix N, and possibly overlapping index sets *Input* and *Output*, indicating which metabolites can be taken up and which can be produced. We now create a new metabolite for each metabolite in *Input* and for each metabolite in *Output*. For convenience, let us

give index I_i to the virtual metabolite that is added if $i \in \textit{Input}$, and the virtual metabolite added if $i \in \textit{Output}$ index O_i. An extended stoichiometry matrix $\bar{N}$ is created by adding rows and columns for these metabolites. The new columns are of the shape $\hat{\boldsymbol{e}}_i - \hat{\boldsymbol{e}}_{I_i}$ and $-\hat{\boldsymbol{e}}_i + \hat{\boldsymbol{e}}_{O_i}$, where $\hat{\boldsymbol{e}}_i$ indicates the i-th elementary unit vector. These new columns model the irreversible 'reactions' $c_{I_i} \to c_i$ and $c_i \to c_{O_i}$. The metabolite c_i is marked as internal, so that the steady-state assumption should be satisfied. We then enumerate the generator description of the conversion cone associated with this extended metabolic network. The results are mapped back to our original metabolic network by the unsplitting rule: $\dot{c}_i = \dot{c}_{I_i} + \dot{c}_{O_i}$.

Theorem 3. *Given a metabolic network, we follow the outlined recipe above to create an extended metabolic network. All Elementary Conversion Modes of the original metabolic network can be found as the generator representation of the conversion cone of the extended metabolic network.*

Proof. Let us first show that there is a bijection f from the space of conversions of the original metabolic network, $\mathcal{C}$, to its image in the space of conversions of the extended metabolic network $\bar{\mathcal{C}}$. Let $\dot{\boldsymbol{c}}$ be a steady-state conversion of the original metabolic network. We can map the conversion to an extended conversion by defining

$$\begin{aligned} f_{I_i}(\dot{\boldsymbol{c}}) &= \dot{c}_i \text{ if } \dot{c}_i < 0 \text{ and } i \in \textit{Input}, \\ f_{O_i}(\dot{\boldsymbol{c}}) &= \dot{c}_i \text{ if } \dot{c}_i > 0 \text{ and } i \in \textit{Output}, \\ f_j(\dot{\boldsymbol{c}}) &= 0 \text{ otherwise.} \end{aligned}$$

This is a steady-state conversion for the extended metabolic network. Its inverse is then given by the unsplitting rule:

$$g_i(\bar{\boldsymbol{x}}) = \bar{x}_{I_i} + \bar{x}_{O_i}.$$

This map is a bijection between the original steady-state conversion cone and its image in the extended cone. This shows that any conversion in the original steady-state conversion cone is still present in the new conversion cone. Now, we only have to show that any ECM of the original metabolic network becomes a generator of the new conversion cone, and vice versa.

We thus first want to show that if $\dot{\boldsymbol{c}}$ is an ECM in the original metabolic network, then $f(\dot{\boldsymbol{c}})$ is a minimal generator of $\bar{\mathcal{C}}$. We will prove this by its contrapositive: assume that $f(\dot{\boldsymbol{c}}) \in \bar{\mathcal{C}}$ is *not* a minimal generator. Then, we know that there are two steady-state conversions $\bar{\boldsymbol{x}}, \bar{\boldsymbol{y}} \in \bar{\mathcal{C}}$ that are not multiples of each other, such that $f(\dot{\boldsymbol{c}}) = \lambda_1 \bar{\boldsymbol{x}} + \lambda_2 \bar{\boldsymbol{y}}$, with $\lambda_1, \lambda_2 > 0$. We can now apply the inverse mapping g to find a similar decomposition in $\mathcal{C}$. According to the definition of ECMs, this shows that $\dot{\boldsymbol{c}}$ is not an ECM, unless for all i we have $\lambda_1 g_i(\bar{\boldsymbol{x}}) = \lambda_2 g_i(\bar{\boldsymbol{y}})$, or if for some i we have $\lambda_1 g_i(\bar{\boldsymbol{x}}) = -\lambda_2 g_i(\bar{\boldsymbol{y}})$. So, we have either $\lambda_1(\bar{x}_{I_i} + \bar{x}_{O_i}) = \lambda_2(\bar{y}_{I_i} + \bar{y}_{O_i})$, or $\lambda_1(\bar{x}_{I_i} + \bar{x}_{O_i}) = -\lambda_2(\bar{y}_{I_i} + \bar{y}_{O_i})$. In addition, we know for each i by the definition of the mapping that either $f_{I_i}(\dot{\boldsymbol{c}}) = 0$, or $f_{O_i}(\dot{\boldsymbol{c}}) = 0$. Let us assume the former without loss of generality, then we know that $\bar{x}_{I_i} = \bar{y}_{I_i} = 0$. Adding the pieces together, we find that either $\lambda_1 \bar{x}_{O_i} = \lambda_2 \bar{y}_{O_i}$, or $\lambda_1 \bar{x}_{O_i} = -\lambda_2 \bar{y}_{O_i}$. Since $\bar{x}_{O_i}, \bar{y}_{O_i} \geq 0$, the latter is impossible, implying that for all i, $\lambda_1 \bar{x}_{O_i} = \lambda_2 \bar{y}_{O_i}$. But this is in direct contradiction with $\bar{\boldsymbol{x}}$ and $\bar{\boldsymbol{y}}$ not being multiples of each other, and $\dot{\boldsymbol{c}}$ can not be an ECM. We conclude that any ECM of the original metabolic network must map to a minimal generating vector of the extended conversion cone.

It remains to be shown that for all vectors $\bar{\boldsymbol{x}}$ in the minimal generating set of $\bar{\mathcal{C}}$, $g(\bar{\boldsymbol{x}})$ is an ECM. Let's again use contrapositivity, such we assume that $g(\bar{\boldsymbol{x}})$ can be written as $g(\bar{\boldsymbol{x}}) = \lambda_1 \dot{\boldsymbol{c}} + \lambda_2 \dot{\boldsymbol{d}}$ so that no metabolite is cancelled in the sum. Even stricter, if we choose appropriate convex combinations of $\lambda_1 \dot{\boldsymbol{c}}$ and $\lambda_2 \dot{\boldsymbol{d}}$, we can find two vectors that we will call $\dot{\boldsymbol{c}}'$ and $\dot{\boldsymbol{d}}'$, for which we have $g(\bar{\boldsymbol{x}}) = \dot{\boldsymbol{c}}' + \dot{\boldsymbol{d}}'$ and that both lie in the same orthant as $g(\bar{\boldsymbol{x}})$. Since these two vectors are not multiples of each other and lie in the same orthant, the mapping f will map them to two vectors that are also not multiples of each other, and for which we still have $\bar{\boldsymbol{x}} = f(\dot{\boldsymbol{c}}') + f(\dot{\boldsymbol{d}}')$. This shows that if $g(\bar{\boldsymbol{x}})$ is an ECM, then $\bar{\boldsymbol{x}}$ cannot be in the minimal generating set of $\bar{\mathcal{C}}$. So, by contrapositivity this means that all vectors in this minimal generating set map to ECMs of the original metabolic network. This completes the proof. □

As we will mention later, the splitting of metabolites can be postponed to later in the calculation if the 'indirect method' is used. This is sometimes beneficial, decreasing both memory usage and computation time, but not always. We have not found a clear indication of when it is useful to postpone the splitting. Therefore, we have added an argument `--splitting_before_polco` that determines when the splitting is done, so that the user can try out which method works best for the model of interest.

3.4 Converting the stoichiometic coefficients into fractions

The ECM enumeration that we implemented works entirely with fractions. This is necessary to keep round-off errors from accumulating which would lead to reporting too many ECMs. Most metabolic networks have some reactions of which the stoichiometric coefficients are decimal numbers: usually the biomass reaction, and sometimes some 'maintenance' reaction. Each number with a finite number of decimals can be exactly converted to a fraction. This is what we do in this step.

It is important to note that coefficients with many decimal numbers will require the numerator and denominator of the fractions to be large numbers. These large integers can slow down the computations. The user might therefore choose to round off the decimal numbers in the input SBML-file if these do not matter too much.

3.5 (Optional): Hiding metabolites and tagging reactions

We have discussed the `--hide`- and `--tag`-methods in Box 2 of the main text. Comma-separated lists can be given to `ecmtool` indicating of which metabolites the production and consumption should be ignored, and of which reactions the rates should be reported. When metabolites are hidden, virtual sink or source reactions are added at this point, and the metabolite is itself marked as internal. When reactions are tagged, virtual metabolites are added that are produced during these reactions.

4 A custom LP-solver

During the enumeration of ECMs we will often encounter Linear Programs of a specific type. We developed a customized LP-solver for this type of problems based on the conventional Revised Simplex Method, see for example.[13] Our solver outperformed other solvers on our type of Linear Programs by both speed and accuracy. In this section we describe the Revised Simplex Method and how we have optimized it.

4.1 Problem description

In the ECM enumeration, we often encounter linear problems where an initial feasible solution is known, but where we want to know if there are alternative solutions. Specifically, let $\boldsymbol{\lambda}_{Init}$ be the initial solution, and let *Init* be the index set of its support. We want to know if there is a solution that uses more than only these reactions. We can use the following Linear Program

$$\begin{aligned} \underset{\boldsymbol{\lambda}}{\text{maximize}} \quad & \sum_{j \notin Init} \lambda_i \\ \text{subject to} \quad & R\boldsymbol{\lambda} = R\boldsymbol{\lambda}_{Init} \\ & \lambda_i \geq 0. \end{aligned} \tag{11}$$

A normal Linear Program would keep searching until the maximizer is found, but we are not interested in the maximizer. Rather, we are interested in *whether* an alternative solution exists. We thus developed an LP-solver with an 'early exit'. Moreover, we used that in this type of problems an initial solution is always known beforehand. Lastly, many of the problems that we will encounter are highly degenerate, as defined below. So, in summary, our problems have three specific features:

- Solver should stop when first alternative solution is found
- Initial feasible solution is known
- Problem is highly degenerate

Before, we can explain how our LP-solver exploits these features, we give a short introduction to Linear Programming. Then, we will describe the revised simplex method, and after that describe how we used and adapted this method.

4.2 Some background on Linear Programming

Let us consider a Linear Program in the form of (11). Let $m \times n$ be the dimensions of R. It is safe to assume $n \geq m$; if this is not the case, there are more linear constraints than variables, and some of them will be redundant.

A *basis* for the LP is any set B of m indices from $\{1, \ldots, n\}$ such that the corresponding columns of R form a basis of the column space of R. In other words, a basis is a B such that the $m \times m$ submatrix R_B of R is non-singular.

We say that a feasible solution $\boldsymbol{\lambda}$ is a *basic feasible solution (BFS)* with basis B if all the non-zero elements of $\boldsymbol{\lambda}$ are also elements of B (B may be a larger index set, though). Note that any basis B has at most one corresponding BFS: since R_B is nonsingular, $R_B\boldsymbol{\lambda} = \boldsymbol{x}$ has a unique solution $\boldsymbol{\lambda_B}$. This $\boldsymbol{\lambda_B}$ is a BFS if and only if it satisfies $\boldsymbol{\lambda_B} \geq \mathbf{0}$. The converse is not true: one BFS can have multiple bases. This occurs when there is a BFS $\boldsymbol{\lambda}$ that has fewer than m non-zero elements. This is called a *degenerate* solution. We will often encounter degenerate solutions. In fact, the initial feasible solution that we will use is almost always degenerate.

Degenerate points can cause *cycling*, a situation where iterative solution methods such as the (revised) simplex method visit the exact same point more than once. When this happens, the method will again start the same cycle, and will thus never terminate. We will overcome this by perturbing the Linear Program, see Section 4.7.

4.3 The revised simplex method

The revised simplex method uses the idea of a basis for a linear program to find an optimal solution. Essentially the method is based on the Karush-Kuhn-Tucker (KKT) conditions, which are necessary and sufficient conditions for the optimality of a Basic Feasible Solution. More information is available in the book *Numerical Optimization*,[13] specifically chapter 12 and 13.

Suppose we have an LP problem in standard form:

$$\begin{aligned} \underset{\boldsymbol{x}}{\text{minimize}} \quad & \boldsymbol{c}^T\boldsymbol{x} \\ \text{subject to} \quad & A\boldsymbol{x} = \boldsymbol{b} \\ & x_i \geq 0, \end{aligned} \tag{12}$$

where we assume A to have size $m \times n$ and rank m. The KKT optimality conditions are:

$$A\boldsymbol{x} = \boldsymbol{b}, \tag{13}$$

$$A^T\boldsymbol{\pi} + \boldsymbol{s} = \boldsymbol{c}, \tag{14}$$

$$\boldsymbol{x} \geq \mathbf{0}, \tag{15}$$

$$\boldsymbol{s} \geq \mathbf{0}, \tag{16}$$

$$\boldsymbol{s}^T\boldsymbol{x} = 0. \tag{17}$$

These conditions derive from constrained optimization using Lagrange multipliers, where $\boldsymbol{\pi}$ is the Lagrange multiplier associated with the constraint $A\boldsymbol{x} = \boldsymbol{b}$, and $\boldsymbol{s}$ is the Lagrange multiplier associated with $\boldsymbol{x} \geq \mathbf{0}$. Practically it means that if we find a combination of $\boldsymbol{x}, \boldsymbol{\pi}$ and $\boldsymbol{s}$ such that all conditions are met, we have found the optimal solution to the LP. These conditions do not only tell us when we are done, but also induce an optimization strategy, which we describe below.

The revised simplex method starts in a Basic Feasible Solution $\boldsymbol{x}$ with feasible basis B of length m. If the problem is non-degenerate, all entries in $\boldsymbol{x}_B$ will be strictly greater than zero. One can thus construct the feasible basis directly from a non-degenerate solution. However, we will often have an initial feasible point with fewer than m nonzero's. In that case, fewer than m columns of A are needed to satisfy the constraints. To still be able to start with the revised simplex method, we must then supply these columns with additional columns from A such that we get an invertible $m \times m$-matrix A_B. The way to find these additional columns is described in Section 4.5. Let us for now proceed by assuming that we have a basis B of size m corresponding to the feasible solution $\boldsymbol{x}$.

We denote by N the indices not in B, and split $\boldsymbol{x}$, $\boldsymbol{c}$ and $\boldsymbol{s}$ according to B:

$$\boldsymbol{x} = \begin{bmatrix} \boldsymbol{x}_B \\ \boldsymbol{x}_N \end{bmatrix} = \begin{bmatrix} A_B^{-1}\boldsymbol{b} \\ \boldsymbol{0} \end{bmatrix}, \qquad \boldsymbol{c} = \begin{bmatrix} \boldsymbol{c}_B \\ \boldsymbol{c}_N \end{bmatrix}, \qquad \boldsymbol{s} = \begin{bmatrix} \boldsymbol{s}_B \\ \boldsymbol{s}_N \end{bmatrix}.$$

Then, we can also split the second KKT constraint in two parts:

$$A_B^T \boldsymbol{\pi} + \boldsymbol{s}_B = \boldsymbol{c}_B, \tag{18}$$

$$A_N^T \boldsymbol{\pi} + \boldsymbol{s}_N = \boldsymbol{c}_N. \tag{19}$$

In order to satisfy the last KKT condition, let $\boldsymbol{s}_B = \boldsymbol{0}$. Since A_B and $\boldsymbol{c}_B$ are known, we can deduce from (18) that

$$\boldsymbol{\pi} = (A_B^T)^{-1} \boldsymbol{c}_B. \tag{20}$$

Next, from (19) we can compute $\boldsymbol{s}_N$ according to:

$$\boldsymbol{s}_N = \boldsymbol{c}_N - A_N^T \boldsymbol{\pi}. \tag{21}$$

Now $\boldsymbol{x}$ satisfies the KKT conditions if and only if $\boldsymbol{s}_N \geq \boldsymbol{0}$, so we can tell whether or not the vertex $\boldsymbol{x}$ is optimal. If $\boldsymbol{x}$ is indeed optimal, we are done and the LP is solved. Otherwise, we perform a *pivot operation.*

4.4 Pivoting: replacing one of the columns in the feasible basis

One step in the revised simplex method involves replacing one column index in B by one from N.

To select the entering index, consider $\boldsymbol{s}_N$. At least one element is negative, otherwise the previous $\boldsymbol{x}$ would have been optimal. We will see later that the entries in $\boldsymbol{s}_N$ capture how much the objective value would decrease when the corrsponding column is added to the basis. Now choose any q with $s_q < 0$ as the entering index. The procedure for altering B and changing $\boldsymbol{x}$ and $\boldsymbol{s}$ accordingly is as follows:

1. Increase x_q from zero;
2. Keep all other components of $\boldsymbol{x}_N$ at zero;
3. Change the current basic vector $\boldsymbol{x}_B$ in such a way that $A\boldsymbol{x} = \boldsymbol{b}$ remains satisfied;
4. Keep increasing x_q until one of the components of $\boldsymbol{x}_B$ (say x_p) reaches zero, or determine that no such component exists (then the LP is unbounded);
5. Remove p from B and replace it with q.

To perform step 3 we can use the following reasoning. Call the new vertex $\boldsymbol{x}^+$. Since both $A\boldsymbol{x} = \boldsymbol{b}$ and $A\boldsymbol{x}^+ = \boldsymbol{b}$, and since $\boldsymbol{x}_N = \boldsymbol{0}$ and $x_i^+ = 0$ for $i \in N \setminus \{q\}$, we have

$$A\boldsymbol{x}^+ = A_B \boldsymbol{x}_B^+ + \boldsymbol{A}_q x_q^+ = A_B \boldsymbol{x}_B = A\boldsymbol{x}. \tag{22}$$

Using that A_B is non-singular, we can left-multiply with A_B^{-1} to obtain

$$\boldsymbol{x}_B^+ = \boldsymbol{x}_B - A_B^{-1} A_q x_q^+ = \boldsymbol{x}_B - \boldsymbol{d} x_q^+, \tag{23}$$

showing that we should subtract from $\boldsymbol{x}_B$ the vector $\boldsymbol{d} = A_B^{-1}\boldsymbol{A}_q$. Step 4 tells us that we should subtract this vector until one of the entries in $\boldsymbol{x}_B^+$ becomes zero. This means that we must have

$$x_q^+ = \min\left\{ \frac{(\boldsymbol{x}_B)_1}{d_1}, \ldots, \frac{(\boldsymbol{x}_B)_m}{d_m} \right\}, \tag{24}$$

and that the leaving column has index $p = \operatorname{argmin}\left\{ \frac{(\boldsymbol{x}_B)_1}{d_1}, \ldots, \frac{(\boldsymbol{x}_B)_m}{d_m} \right\}$.

We can verify that this pivot operation always leads to a decrease in the objective $\boldsymbol{c}^T\boldsymbol{x}$.

Theorem 4. *The above described pivot operation will always lead to a decrease in the objective function. The change is always strictly less than zero if the original solution $\boldsymbol{x}$ is non-degenerate.*

Proof. We know that

$$\boldsymbol{x}_N^+ = (0, \dots, 0, x_q^+, 0, \dots, 0)^T, \tag{25}$$

so

$$\begin{aligned}\boldsymbol{c}^T\boldsymbol{x}^+ &= \boldsymbol{c}_B^T\boldsymbol{x}_B^+ + \boldsymbol{c}_N^T\boldsymbol{x}_N^+ \\ &= \boldsymbol{c}_B^T\boldsymbol{x}_B^+ + c_q x_q^+ \\ &= \boldsymbol{c}_B^T\boldsymbol{x}_B - \boldsymbol{c}_B^T A_B^{-1}\boldsymbol{A}_q x_q^+ + c_q x_q^+.\end{aligned} \tag{26}$$

From (20) we have $\boldsymbol{c}_B^T A_B^{-1} = \boldsymbol{\pi}^T$, and from (19) we get $\boldsymbol{\pi}^T\boldsymbol{A}_q = c_q - s_q$. Therefore,

$$\boldsymbol{c}_B^T A_B^{-1}\boldsymbol{A}_q x_q^+ = \boldsymbol{\pi}^T\boldsymbol{A}_q x_q^+ = (c_q - s_q)x_q^+, \tag{27}$$

so by substituting in (26) we obtain

$$\boldsymbol{c}^T\boldsymbol{x}^+ = \boldsymbol{c}_B^T\boldsymbol{x}_B - (c_q - s_q)x_q^+ + c_q x_q^+ = \boldsymbol{c}_B^T\boldsymbol{x}_B - s_q x_q^+. \tag{28}$$

Since $\boldsymbol{x_N} = 0$, we have $\boldsymbol{c}^T\boldsymbol{x} = \boldsymbol{c}_B^T\boldsymbol{x}_B$ and therefore

$$\boldsymbol{c}^T\boldsymbol{x}^+ = \boldsymbol{c}^T\boldsymbol{x} - s_q x_q^+. \tag{29}$$

We chose q such that $s_q < 0$, and since $x_q^+ \geq 0$, it follows that the step produces a decrease in the objective function $\boldsymbol{c}^T\boldsymbol{x}$. The change in objective value is strictly less than zero whenever x_q^+ is strictly larger than zero. Since $x_q^+ = \min\left\{\frac{(\boldsymbol{x}_B)_1}{d_1}, \dots, \frac{(\boldsymbol{x}_B)_m}{d_m}\right\}$, we are sure to have a strict decrease whenever $\boldsymbol{x}_B > \boldsymbol{0}$, which is the definition of a non-degenerate solution. □

It is important to emphasize what happens when we have a degenerate solution. In that case, one of the entries in $\boldsymbol{x}_B$ is zero, and the pivot operation might thus lead to a step of size zero: column p was used zero times in the original solution, and is now replaced by column q which is also used zero times. In this case, and in this case only, the objective value does not strictly decrease during the pivot operation. This can be problematic, because the simplex method could later return to the same basis. If the same pivot step is again made at that point, one could end up in an infinite cycle. Such cycling is impossible in the non-degenerate case, since the strict decrease in objective value prevents the same basis from re-occurring. This problem can be prevented by keeping track of the bases that have already been visited, or by perturbing the system to get rid of degeneracy. We will describe in Section 4.7 that we choose the latter.

4.5 Finding a starting basis efficiently

As mentioned in Section 4.1, we always already have an initial feasible solution for the Linear Programs that we must solve in this work. This initial $\boldsymbol{x}$ satisifies the constraints, but it is not yet a Basic Feasible Solution. For that we need an index set B of length m that contains at least all indices that correspond to the nonzero's of $\boldsymbol{x}$, and such that A_B is non-singular. Because our initial feasible solutions are often degenerate, $\boldsymbol{x}$ generally comprises fewer than m nonzero entries, so that we should supply B with columns of A that do not contribute to satisfying the constraints, i.e., the corresponding entry in $\boldsymbol{x}$ is zero.

To do this, we start by taking the indices in which $\boldsymbol{x}$ is non-zero. The columns corresponding to these indices should be linearly independent, otherwise we must find an alternative solution where one of the columns is no longer necessary. However, in the problems that we will encounter, the linear independence is guaranteed by the preceding steps. Then, we start iterating over the columns of A: we try to add a column and check to see if the resulting matrix is still of maximal rank. When this is not the case, the most recently added column is dropped again. After trying this for all columns of A, we are guaranteed to find m total columns that are linearly independent, since A has rank m.

Finding a basis in this manner is a time-consuming computation, but we do not have to go through this procedure each time that we do an LP. Indeed, as will become clear in Sections 7.3.3 and 8.1.2, we will need to do many LPs for the same constraint matrix A, but with a different initial feasible solution $\boldsymbol{x}$. This means that we can use the basis B found in the previous LP as a starting point. We only have to make sure that the indices corresponding to the nonzero's in $\boldsymbol{x}$ are in B. Therefore, we use the following method.

Let A_B be an invertible matrix, and $\boldsymbol{A}_q$ a column. We want to construct a new invertible matrix $\bar{A}_B$ by replacing one of the columns from A_B by $\boldsymbol{A}_q$. We solve

$$A_B \boldsymbol{x} = \boldsymbol{A}_q, \tag{30}$$

which according to Cramer's rule gives

$$x_i = \frac{\det(A_B^{(i)})}{\det(A_B)}, \tag{31}$$

where $A_B^{(i)}$ is the matrix formed by replacing the i-th column of A_B by $\boldsymbol{A}_q$. Since $\boldsymbol{A}_q$ is nonzero, there must be an i for which $x_i \neq 0$, which implies that $\det(A_B^{(i)}) \neq 0$. We can thus find a new invertible matrix by replacing the i-th column of A_B by $\boldsymbol{A}_q$.

4.6 Detecting an early exit possibility

As mentioned in Section 4.1, the LP-solver may stop when it is clear that the initial solution is not the only possible solution. This means that we can stop the program at two events: 1) when the KKT conditions are met, meaning that the initial feasible solution is the only (and thus the optimal) solution, or 2) when the first non-zero step is made during a pivot operation, showing that an alternative solution exists.

From (24), we know that the step size is equal to $\min\left\{\frac{(\boldsymbol{x}_B)_1}{d_1}, \ldots, \frac{(\boldsymbol{x}_B)_m}{d_m}\right\}$, and that the index that leaves the basis B is then equal to $\operatorname{argmin}\left\{\frac{(\boldsymbol{x}_B)_1}{d_1}, \ldots, \frac{(\boldsymbol{x}_B)_m}{d_m}\right\}$. Therefore, it is clear that a nonzero step is only made when the leaving index corresponds to a nonzero entry of $\boldsymbol{x}$. This means that we can stop the Linear Program when the leaving index corresponds to a nonzero entry of the initial solution $\boldsymbol{x}$.

4.7 Perturbation to remove degeneracy

Degenerate vertices are problematic for the (revised) simplex method. When $\boldsymbol{x}$ is degenerate, the pivot operation described above might not cause any change in $\boldsymbol{x}$ at all. It is possible to make a number of degenerate pivots resulting in the same basis we had before. In this case the algorithm would start cycling and never terminate.

We use a perturbation strategy to circumvent this problem. We will call the original Linear Program LP and the perturbed one LP'. The perturbation is applied after we have established a Basic Feasible Solution, with solution $\boldsymbol{x}$, and corresponding basis B. This solution of course satisfies the original constraint

$$A\boldsymbol{x} = \boldsymbol{b}. \tag{32}$$

However, we now perturb the right hand side of this equation to get

$$A\boldsymbol{x'} = \boldsymbol{b'} = \boldsymbol{b} + A_B \boldsymbol{\varepsilon}, \tag{33}$$

where $\boldsymbol{\varepsilon}$ is a vector of length m with elements chosen uniformly at random from $[\delta/2, \delta]$, where $\delta > 0$ is a small constant. Note that $\boldsymbol{\varepsilon} > \boldsymbol{0}$. The solution corresponding to this new constraint is

$$\boldsymbol{x'} = A_B^{-1}\boldsymbol{b'} = A_B^{-1}(\boldsymbol{b} + A_B\boldsymbol{\varepsilon}) = \boldsymbol{x} + \boldsymbol{\varepsilon}. \tag{34}$$

Since we chose $\boldsymbol{\varepsilon} > \boldsymbol{0}$, this new solution still satisfies $\boldsymbol{x'} \geq \boldsymbol{0}$, and B is still a feasible basis for the perturbed problem LP'.

Theorem 5 (properties of perturbed LP)**.** *The following hold for LP':*
(a) LP' is non-degenerate.
(b) If B is a feasible basis of LP', then B is also a feasible basis of LP.
(c) If B is an optimal basis of LP', then B is also an optimal basis of LP.
(d) If x_q can leave and x_p can enter in a pivot corresponding to B in LP', then the same holds in LP.

See for example[14] for a proof. This theorem shows that we can use the perturbed version LP' to do all the pivots, and once we find an optimal basis it is guaranteed that this basis is also optimal for the original problem. This shows that although the perturbation might affect the exact optimal value of the LP, it will never affect whether there is an alternative solution to the initial solution, or not. For our purposes, the perturbation of the LP thus has no disadvantages, while it does prevent the revised simplex method from entering an infinite loop.

5 Compression of the metabolic networks

To reduce the size of the main step in ECM-enumeration, the metabolic network can be compressed by various methods. The compression steps that we have used all leave the eventual set of ECMs unchanged. The amount by which the network can be compressed is one of the advantages of ECM-enumeration compared to EFM-enumeration: because the individual reaction rates are not reported, many reactions can be merged or even deleted. The first four compression steps are adapted from,[10] the compression of cycles and the removal of redundant rays has been added by us.

These compression steps are not completely independent. If compression step A is executed, then another execution of A will not remove any more metabolites or reactions. However, after executing compression step B, A might again be able to compress the network further. Therefore, to maximise the compression of the network, the following sequence of compression steps is repeated until a complete sequence did not remove another metabolite or reaction.

5.1 Removal of infeasible reactions

Some reactions can never be active in a steady state solution that satisfies the irreversibility constraints. Before we start the ECM enumeration, these reactions can be safely removed, since we are only looking for steady state conversions.

A first test if a reaction is feasible can be done by calculating the nullspace of the matrix N_{int}, denoting the part of the stoichiometric matrix corresponding to internal metabolites. Let K be a matrix with as columns a basis for the nullspace, so that we have for each column $NK_{\bullet i} = 0$. Note that the nullspace contains the full steady state flux cone. An entry k_{ji} of the matrix K denotes the rate of reaction j in the i-th vector of the nullspace. If K contains a row of only zeros, this means that this reaction can never be active in a steady state solution. The reactions corresponding to zero rows of K can thus be removed from the network.

The nullspace contains all solutions that satisfy the steady state constraint $N_{int}\boldsymbol{v} = 0$, but might also include solutions that do not satisfy the irreversibility constraints: $v_i \geq 0$ for all irreversible reactions i. Therefore, we can do another test, suggested by Urbanczik et al.[10] to check if reactions become infeasible if we take these irreversibility constraints into account.

All solutions that satisfy the steady state constraint can be written as $v = K\lambda$, because the columns of K form a nullspace of N. Here, the rate of the i-th reaction in the solution is given by $v_i = K_{i\bullet}\lambda$. We can get a sum of reaction rates by left-multiplying $v = K\lambda$ by a vector: $\mu K\lambda = \sum_i \mu_i v_i$. To test for reaction feasibility, we select the submatrix K_{irr} of K given by all rows that correspond to irreversible reactions. Then, we check, with a Linear Program, if there exists a non-zero vector μ such that $\mu_i \geq 0$ for all i, and $\mu K_{irr} = 0$. If such a vector exists, this means that for any λ we have $0 = \mu K_{irr}\lambda = \sum_{\text{irreversible}} \mu_i v_i$. In other words, for all steady state solutions, the sum of reaction rates with weights μ_i is zero. However, since all μ_i are positive, this implies that some of the v_i must be negative, but this would violate the irreversibility constraints. Therefore, the only option is that all v_i are zero, and thus infeasible. Concluding, all reactions i such that μ_i is strictly greater than zero must be zero, are thus infeasible, and can be removed.

5.2 Dead-end metabolites are deleted

Internal metabolites that can either only be produced or only be consumed, are sometimes called *dead-end metabolites*. The ECMs are calculated under the constraint that all internal metabolites are in steady-state. A dead-end metabolite can therefore not be produced/consumed at all, because there are no consuming/producing reactions to maintain the steady-state. Before ECM-computation we can therefore delete these metabolites and all (solely producing or consuming) reactions that connect to them.

5.3 Cancelling metabolites with a reversible reaction

A reversible reaction can be used to delete an internal metabolite without changing the space of steady-state conversions, see Figure S4b. Let's say that we have a metabolic network with r reactions and that the last one of those is reversible. We also assume that there is at least one internal metabolite

involved in this reaction, say metabolite i. Now, we are going to cancel the production or consumption of metabolite i from each reaction by adding or subtracting reversible reaction r. We denote by ϵ_j^r the number of times that we need to add reaction r to reaction j to cancel metabolite i. The resulting stoichiometric matrix, $\bar{N}$ thus has the same number of columns, but only column r still has a non-zero i-th entry. To see that this change of stoichiometry does not change the steady-state conversion space, let's recall the definition:

$$\mathcal{C} = \{\dot{\boldsymbol{c}} = N\boldsymbol{v} \mid N_{\text{Int}}\boldsymbol{v} = \boldsymbol{0},\ v_i \geq 0 \text{ if } i \text{ irreversible}\}. \tag{35}$$

Let $\dot{\boldsymbol{c}} \in \mathcal{C}$ be a conversion in the original metabolic network, and let $\boldsymbol{v}$ be a flux vector that leads to this conversion. Then we know that $\dot{\boldsymbol{c}} = N\boldsymbol{v} = \sum_j \boldsymbol{N}_{\bullet j} v_j$. We now prove that this conversion can still be generated with the new stoichiometric matrix $\bar{N}$. We have

$$\dot{\boldsymbol{c}} = \sum_j \boldsymbol{N}_{\bullet j} v_j = \sum_j (\bar{\boldsymbol{N}}_{\bullet j} - \epsilon_j^r \bar{\boldsymbol{N}}_{\bullet r}) v_j = \bar{N}\bar{\boldsymbol{v}}, \tag{36}$$

where $\bar{\boldsymbol{v}} = \boldsymbol{v} + \sum_j \epsilon_j^r \hat{\boldsymbol{e}}_r$. Since reaction r was reversible, v_r is allowed to be negative. Therefore, $\bar{\boldsymbol{v}}$ is certainly a feasible steady-state flux vector, and the original conversion $\dot{\boldsymbol{c}}$ is therefore still possible. This compression is not possible for irreversible reactions, because $\sum_j \epsilon_j^r$ might be negative.

The advantage of changing the stoichiometric matrix from N to $\bar{N}$ is that we are now in the situation of Section 5.2: metabolite i has become a dead-end metabolite. We can therefore now cancel both metabolite i and reaction r. In Figure S4b we illustrate how reversible reaction v_3 can be used to cancel metabolite x_2.

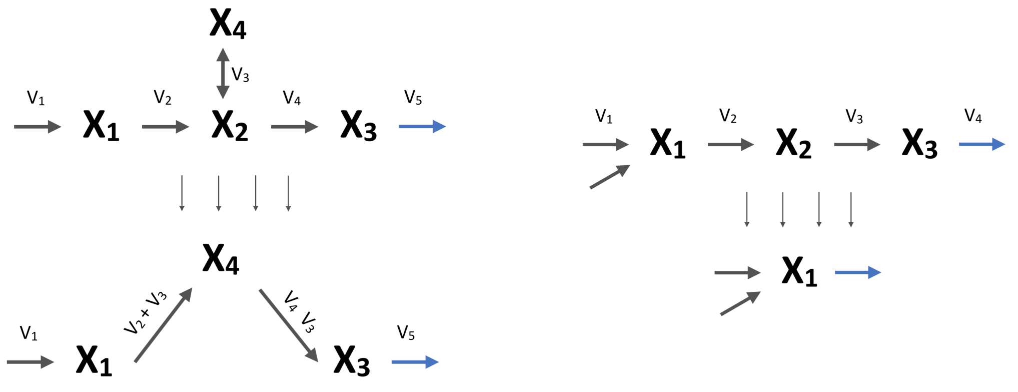

(a) (b)

Figure S4: **Conversion cones are invariant to network compression.** (a) Compression through addition of a reversible reaction to other reactions. V_3 is added to V_2 and subtracted from V_4. This sets the stoichiometry of X_2 to 0 in V_2 and V_4. Since X_2 is now only used in V_3, they can both be removed. (b) Compression through the removal of singly produced or consumed internal metabolites. As X_2 is only produced by V_2, V_2 can be added directly to V_3. Since X_2 is now only used in V_2, both X_2 and V_2 can be removed. The same can then be done for X_3.

5.4 Cancelling singly produced or consumed metabolites

A metabolite that is either produced by only one reaction, or consumed by only one reaction, can be cancelled without changing the space of steady-state conversions, see Figure S4a. Let's say that reaction r is the only reaction in the metabolic network that produces metabolite i. (The case in which metabolite i is consumed by only one reaction is similar and will thus not be treated here.) We can now add reaction r to all reactions j that consume metabolite i, such that the consumption of i is cancelled exactly. We again denote by ϵ_j^r the number of times that we need to add reaction r to reaction j to cancel the

consumption of metabolite i. The modified stoichiometric matrix is given by $\bar{N}$. Note that in this case, all ϵ_j^r are nonnegative. For a general conversion $\dot{\boldsymbol{c}}$ we again get

$$\dot{\boldsymbol{c}} = \sum_j \boldsymbol{N}_{\bullet j} v_j = \sum_j (\bar{\boldsymbol{N}}_{\bullet j} - \epsilon_j^r \bar{\boldsymbol{N}}_{\bullet r}) v_j = \bar{N}\bar{\boldsymbol{v}}, \tag{37}$$

where $\bar{\boldsymbol{v}} = \boldsymbol{v} + \sum_j \epsilon_j^r \hat{\boldsymbol{e}}_r$. Because all $\epsilon_j^r \geq 0$, this change will not violate the irreversibility constraints, and therefore all conversions remain feasible in the modified metabolic network.

Again we end up in the situation of Section 5.2: metabolite i and reaction r can be removed. In Figure S4a we illustrate how, for example, complete linear pathways can be reduced to just one reaction by this method.

5.5 Removing cycles by cancelling metabolites

It is possible that the metabolic network contains *cycles*: combinations of reactions that have a combined production and consumption of zero. These combinations of reactions can also be viewed as vectors $\boldsymbol{v}$ that satisfy the irreversibility constraints and are in the nullspace of the stoichiometric matrix: $N\boldsymbol{v} = \boldsymbol{0}$. We will show that these cycles can in some sense be viewed as reversible reactions, and can therefore be used to cancel reactions and metabolites. Moreover, for the direct intersection method that we will describe below, it is necessary that the cone spanned by the columns of N is pointed (see Section 1.2), which means that cycles may not exist. For the use of this intersection method, this compression step therefore must be applied.

We will detect cycles in N by solving a Linear Program with our custom LP-solver described in Section 4. We will try to find a $\boldsymbol{\lambda} \geq \boldsymbol{0}$ that satisfies $N\boldsymbol{\lambda} = \boldsymbol{0}$ and $\boldsymbol{\lambda} \neq \boldsymbol{0}$; if such a $\boldsymbol{\lambda}$ exists, there are still cycles. The cycle finding LP is as follows:

$$\begin{aligned} \underset{\boldsymbol{\lambda}}{\text{maximize}} \quad & \sum_i \lambda_i \\ \text{subject to} \quad & N\boldsymbol{\lambda} = \boldsymbol{0} \\ & \lambda_i \geq 0 \\ & \lambda_i \leq 1. \end{aligned} \tag{38}$$

The LP is always feasible, since $\boldsymbol{\lambda} = \boldsymbol{0}$ is a solution with objective value 0. If this is the only feasible solution, then we are sure that cycles no longer exist in the metabolic network, so that this compression step is done. If another feasible solution exists, it will always be found by the Linear Program because it must result in a larger objective value.

Let's say that we have a cycle, then the optimal solution $\boldsymbol{\lambda}^*$ may be assumed to have at least one position equal to 1, say $\lambda_r^* = 1$. This is because any $\boldsymbol{\lambda}$ that induces a cycle satisfies $N\boldsymbol{\lambda} = \boldsymbol{0}$, so multiples of $\boldsymbol{\lambda}$ will satisfy that as well. Hence the constraint $\lambda_i \leq 1$ (for all i) is the only thing keeping λ (and with that the optimal value) bounded. The corresponding column $\boldsymbol{N}_{\bullet r}$ gives the stoichiometry of a reaction that is involved in the cycle. This will be used to remove (part of) the cycle.

Let's say that metabolite i is produced by reaction r. Because we have split each external metabolite into being only an input or only an output in Section 3.3, it is impossible for external metabolites to have non-zero coefficients in any ray that is part of a cycle, as there can be no circular flow through such external metabolites. We can thus be assured that metabolite i is an internal metabolite.

Because reaction r is part of a cycle, we have

$$\boldsymbol{0} = \sum_j \boldsymbol{N}_{\bullet j} v_j = \sum_{j \neq r} \boldsymbol{N}_{\bullet j} v_j + \boldsymbol{N}_{\bullet r} v_r = N\boldsymbol{v}^- + \boldsymbol{N}_{\bullet r} v_r, \tag{39}$$

where $\boldsymbol{v}^-$ are all reaction rates in the cycle except for reaction r: $\boldsymbol{v}^- = \boldsymbol{v} - v_r \hat{\boldsymbol{e}_r}$. Comparing this with the case in which we had a reversible reaction, reaction r can now be viewed as the forward reaction, and the combination of the other reactions: $N\boldsymbol{v}^-$ as the backward reaction. Let us in fact add this combination of reactions as reaction $r+1$ to our network. We can now use these two 'reactions' to cancel the production and consumption of metabolite i from all reactions in the network. If a reaction consumes metabolite i, then we will cancel this consumption by adding reaction r; let ϵ_j^r denote the number of

times that reaction r is added to reaction j. If reaction j produces metabolite i, then we can cancel the production by adding reaction $r+1$; ϵ_j^{r+1} denote how many times this is necessary. We then get for any conversion that was feasible in the original metabolic network that

$$\dot{\boldsymbol{c}} = \sum_j \boldsymbol{N}_{\bullet j} v_j = \sum_j (\bar{\boldsymbol{N}}_{\bullet j} - \epsilon_j^r \bar{\boldsymbol{N}}_{\bullet r} - \epsilon_j^{r+1} \bar{\boldsymbol{N}}_{\bullet r+1}) v_j = \bar{N} \bar{\boldsymbol{v}}, \tag{40}$$

where $\bar{\boldsymbol{v}} = \boldsymbol{v} + \sum_j \epsilon_j^r \hat{\boldsymbol{e}}_r + \sum_j \epsilon_j^{r+1} \hat{\boldsymbol{e}}_{r+1}$. Since all ϵ-values are now nonnegative, we now that this flux vector meets the irreversibility constraints, and thus that the conversion is still possible in the modified metabolic network.

Finally, we are left with a metabolic network in which metabolite i is produced by reaction r and consumed by reaction $r+1$, and these reactions are their exact reverse. It is clear that the steady-state constraint imposes $v_r = v_{r+1}$ which means that the net contribution of these reactions is always zero. Therefore, we can delete both reaction r, reaction $r+1$, and metabolite i from the network.

After this procedure we have deleted (at least) one metabolite and one reaction from the orginal metabolic network. It is often the case that cycles still remain, so that the cycle removal procedure has to be repeated until no cycles are left.

5.5.1 Example

In the metabolic network of Figure S5, there is one cycle: (B $\to$ C $\to$ F $\to$ E $\to$ B). The cycle finding LP described above would find the solution $\boldsymbol{\lambda}^* = (0,0,1,1,1,1,0,0,0,0)$. So we learn that reactions 3, 4, 5 and 6 are involved in a cycle. We select reaction 3 and metabolite B for the removal of the cycle.

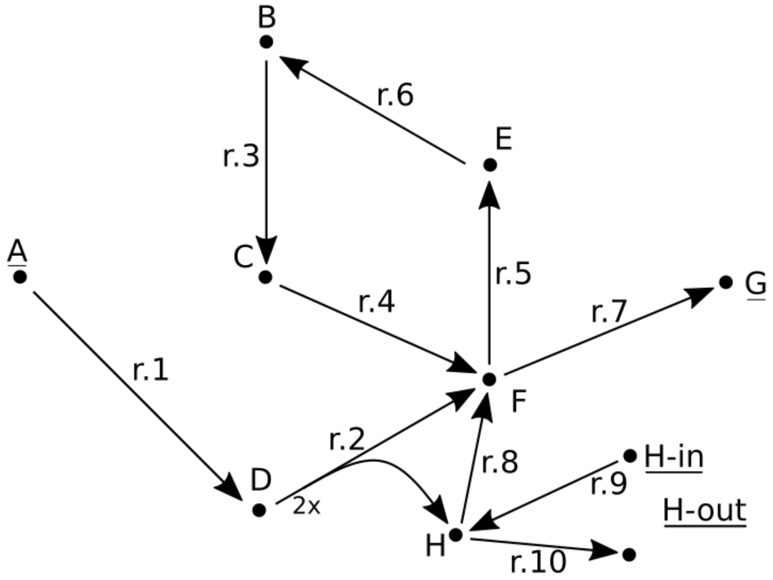

Figure S5: Example network for cycle removal.

The only other reaction that uses B is reaction 6. Therefore we add reaction 3 to reaction 6 to create a new ray that produces C out of E. Then, reaction 3 can be removed. The resulting network can be seen in Figure S6. After three more steps, the network will be cycle-free (Figure S7).

5.6 Removal of redundant reactions

We call reaction r *redundant* if there is a feasible combination $\boldsymbol{w}$ of different reactions such that

$$\boldsymbol{N}_{\bullet r} = \sum_{j \neq r} \boldsymbol{N}_{\bullet j} w_j. \tag{41}$$

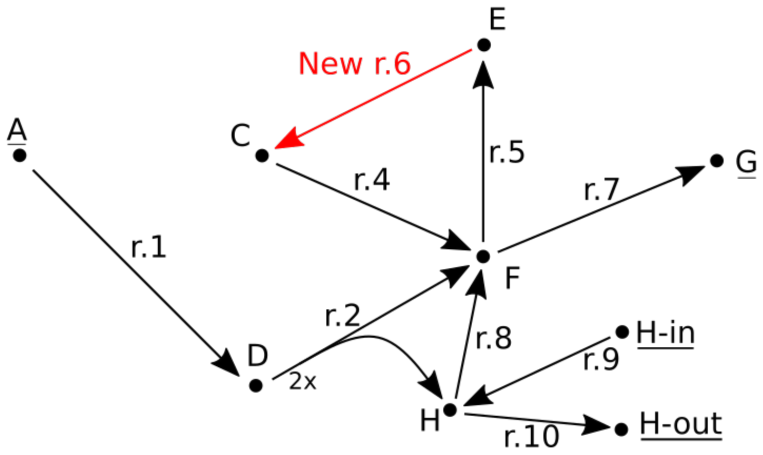

Figure S6: Network after one cycle removing step.

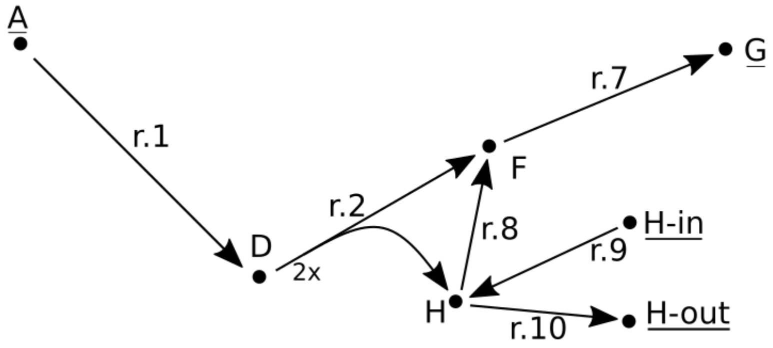

Figure S7: Network after all cycles have been removed.

These redundant reactions can be removed without changing the steady-state conversion space. To see this, let $\dot{\boldsymbol{c}} \in \mathcal{C}$ be any conversion, then we have

$$\dot{\boldsymbol{c}} = N\boldsymbol{v} = \sum_j \boldsymbol{N}_{\bullet j} v_j = \sum_{j \neq r} \boldsymbol{N}_{\bullet j} v_j + \boldsymbol{N}_{\bullet r} v_r = \sum_{j \neq r} \boldsymbol{N}_{\bullet j} v_j + \sum_{j \neq r} \boldsymbol{N}_{\bullet j} w_j v_r = \sum_{j \neq r} \boldsymbol{N}_{\bullet j} (v_j + w_j v_r). \quad (42)$$

So, all steady state conversions remain feasible if we remove the redundant reaction from the network. The redundant reactions can be removed using `redund` from `lrslib`.[8] Although this program works well on relatively small sets of vectors (up to hundreds), it is very slow on larger sets. We have therefore developed our own redundancy-removal method that uses the following Linear Program:

$$\begin{aligned} \underset{\boldsymbol{v}}{\text{maximize}} \quad & \sum_{j \neq r} v_i \\ \text{subject to} \quad & N\boldsymbol{v} = \boldsymbol{N}_{\bullet r} \\ & v_i \geq 0. \end{aligned} \quad (43)$$

In this LP, we are not necessarily interested in the maximum, but rather in the question whether there is a solution besides $\boldsymbol{v} = \hat{\boldsymbol{e}}_{\boldsymbol{r}}$. This means that we can quit the program once it is clear that such an alternative solution exists. This is exactly the type of problem for which we designed the LP-solver described in Section 4. Note that this LP becomes unbounded whenever the cone generated by the columns of N is non-pointed. In this case, our strategy would not work. Therefore, it is important to remove all cycles before removing redundant reactions (Section 5.5).

6 The starting point: generator representation of the (non-steady-state) conversion cone

After the metabolic network has been preprocessed (Section 3) and compressed (Section 5), we are now ready for the main computational task. Let us therefore recall the goal of ECM enumeration. We want to describe the *steady-state conversion cone*

$$\mathcal{C} = \{\dot{\boldsymbol{c}} = N\boldsymbol{v} \ | \ N_{\text{Int}}\boldsymbol{v} = \boldsymbol{0}, \ v_i \geq 0 \text{ if reaction } i \text{ irreversible}\}\,. \tag{44}$$

Assuming that this cone is fully contained in one orthant, which can be assured by splitting metabolites (Section 3.3), the Elementary Conversion Modes can be found as a minimal set of generators of this cone.

Both the indirect method (Section 7) and the direct method (Section 8) start from a generator representation of a larger cone: the *(non-steady-state) conversion cone*,

$$\mathcal{C}_0 = \{\dot{\boldsymbol{c}} = N\boldsymbol{v} \ | \ v_i \geq 0 \text{ if reaction } i \text{ irreversible}\}\,, \tag{45}$$

which comprises all conversions, including those that do not satisfy the steady-state constraint. We can assume without loss of generality that all reactions in the metabolic network are irreversible, since they were either cancelled during the compression step, or they were split into a forward and backward reaction. In that case, (45) already gives a generator representation: the columns of the stoichiometry matrix, N, generate all possible conversions. The remaining task is to impose the steady-state constraints while keeping track of a generator representation. We will describe the two methods that we implemented to accomplish this.

The cone $\mathcal{C}_0$ is generally not contained in one orthant, nor is it necessarily pointed. In Section 3.3 we describe why it is beneficial to split external metabolites such that $\mathcal{C}$ is contained in one orthant, but this is not yet necessary for this initial cone $\mathcal{C}_0$. We will describe below that in the indirect method, external metabolites do not even have to be split already because it can be done later. The same holds for the pointedness: we cannot use the direct method on a non-pointed $\mathcal{C}_0$, while it will give no problems in the indirect method. Therefore, `ecmtool` always removes cycles before using the direct method, thereby making $\mathcal{C}_0$ pointed (Section 5.5).

7 The indirect method

The indirect method of ECM enumeration is based on the method introduced by Urbanczik et al.[10] It is based on the fact that it is relatively easy to calculate a generator representation of a polyhedral cone when an inequality representation is known. This task can be accomplished by the Double Description (DD) method,[7] and we use `Polco` for this.[9] Crucially, the indirect method depends on the connection between the generator representation of a cone and the inequality representation of its dual, see Section 1.3 for background information about this connection. More precisely, we will follow the following steps to go from the generator representation (45) to the set of ECMs:

$$\text{gen}(\mathcal{C}_0) = \text{ineq}(\mathcal{C}_0^*) \overset{\text{DD}}{\to} \text{gen}(\mathcal{C}_0^*) = \text{ineq}(\mathcal{C}_0) \overset{\text{SS constraints}}{\longrightarrow} \text{ineq}(\mathcal{C}) \overset{\text{DD}}{\to} \text{gen}(\mathcal{C}) = \text{ECMs} \tag{46}$$

One might at this point ask why the steady-state constraints cannot be added at the start, following the path $\text{gen}(\mathcal{C}_0) \to \text{gen}(\mathcal{C})$ at once. The answer is that this is indeed possible, and this is the strategy implemented by the direct method (Section 8). However, it is generally harder to impose equality constraints to a generator representation than to an inequality representation, where they can just be added as two inequality constraints to the existing representation. This is why this indirect route is in many cases, but not always, preferable, and this intersection method is therefore the default in `ecmtool`, which can be changed by setting `--direct True`.

In the following subsections we will give the details of the method, and where we have added optimization steps.

7.1 $\text{gen}(\mathcal{C}_0) = \text{ineq}(\mathcal{C}_0^*)$

The columns of the stoichiometry matrix form a generator representation of the non-steady-state conversion cone (see (45)). In Section 1.3 we then explained that this gives an inequality representation to the

dual cone: the constraint matrix being the transpose of the stoichiometry matrix. However, this dual cone does not need to be pointed, see Section 1.2. The problem with non-pointed cones is that generally they do not have a unique generator representation. The next step in the method, $\text{ineq}(\mathcal{C}_0^*) \overset{\text{DD}}{\to} \text{gen}(\mathcal{C}_0^*)$, would therefore not be well-defined. To still be able to apply the Double Description method, we decompose our cone in its lineality space and its pointed part, according to

$$\begin{aligned} \mathcal{C}_0^* &= \text{Lin}(\mathcal{C}_0^*) \oplus (\mathcal{C}_0^* \cap \text{Lin}(\mathcal{C}_0^*)^{\perp}), \\ &= \left\{ \boldsymbol{x} \in \mathbb{R}^d \;\middle|\; N^T\boldsymbol{x} = \boldsymbol{0} \right\} \oplus \left\{ \boldsymbol{x} \in \mathbb{R}^d \;\middle|\; N^T\boldsymbol{x} \geq \boldsymbol{0}, \quad \text{Null}(N^T)^T\boldsymbol{x} = \boldsymbol{0} \right\}, \end{aligned} \tag{47}$$

where $\text{Null}(N^T) = \begin{bmatrix} \boldsymbol{n}_1 & \cdots & \boldsymbol{n}_k \end{bmatrix}$ is a matrix constructed from a basis of the nullspace of N^T. This shows that it is necessary to find a basis for the nullspace of N^T. First of all, because $\{\boldsymbol{n}_1, \dots, \boldsymbol{n}_k, -\boldsymbol{n}_1, \dots, -\boldsymbol{n}_k\}$ gives a generator representation of the lineality space (first part in (47)). Second, because we need this basis to complete the inequality representation of the pointed part of $\mathcal{C}_0^*$ (second part in (47)).

7.1.1 An iterative symbolic nullspace calculation

It turns out that obtaining an exact basis for a nullspace for a large matrix with potentially large fractions is not a trivial computational task. We cannot resort to faster methods using floats, because this will induce round-off errors that might further accumulate during the ECM-enumeration. We have therefore implemented an iterative nullspace calculation which avoids memory issues. For this, we separate rows of N^T into K parts, denoting the first set of rows by $N_{(1)}$. We calculate a basis for its nullspace using a symbolic solver, and gather the basis vectors as the columns of a matrix M. We then know that any vector $\boldsymbol{x}$ in the nullspace of N^T should be a linear combination of the columns in M, in other words: $\boldsymbol{x} = M\boldsymbol{\lambda}$, for some real vector $\boldsymbol{\lambda}$. We now take the second part of our matrix, $N_{(2)}$. We should have $N_{(2)}\boldsymbol{x} = \boldsymbol{0}$, so that $\boldsymbol{\lambda}$ should satisfy $N_{(2)}M\boldsymbol{\lambda} = \boldsymbol{0}$. Therefore, we define a matrix $N_{(1:2)} := N_{(2)}M$, and calculate its nullspace. Again we gather a basis for this nullspace as the columns of M. Proceeding in this fashion, we will eventually obtain a matrix M containing a basis for the nullspace of $N_{(1:K)} = N^T$.

7.2 $\textbf{ineq}(\mathcal{C}_0^*) \overset{\textbf{DD}}{\to} \textbf{gen}(\mathcal{C}_0^*)$

The dual of the non-steady-state conversion cone, $\mathcal{C}_0^*$ consists of the two parts given in (47). Fortunately, a set of generators of the entire space is just given by the union of the generators of both parts:

$$\begin{aligned} \text{gen}\left(\mathcal{C}_0^*\right) &= \text{gen}\left(\text{Lin}(\mathcal{C}_0^*)\right) \cup \text{gen}\left((\mathcal{C}_0^* \cap \text{Lin}(\mathcal{C}_0^*)^{\perp})\right), \\ &= \{\boldsymbol{n}_1, \dots \boldsymbol{n}_k, -\boldsymbol{n}_1, \dots \boldsymbol{n}_k\} \cup \text{DD}\left(\left\{ \boldsymbol{x} \in \mathbb{R}^d \;\middle|\; N^T\boldsymbol{x} \geq \boldsymbol{0}, \quad \text{Null}(N^T)^T\boldsymbol{x} = \boldsymbol{0} \right\}\right) \end{aligned}$$

Note that the first part of this generator representation is not necessarily unique, because there is no unique basis for the nullspace. However, since this generator representation is only an intermediate result, this does not matter. What matters is that the cone described by this generator representation is unique.

The Double Description method that we use to get the second part of the generator representation is described in Section 1.5 and implemented in `Polco`.[9] This step, like all others, is done using fractions, so that our ECM-computations remain exact.

7.3 $\textbf{gen}(\mathcal{C}_0^*) = \textbf{ineq}(\mathcal{C}_0) \overset{\textbf{SS constraints}}{\longrightarrow} \textbf{ineq}(\mathcal{C})$

In the previous sections we have described how we obtain a generator representation of the dual of the (non-steady-state) conversion cone $\mathcal{C}_0^*$. This generator representation also forms an inequality representation of $\mathcal{C}_0$. Let us gather all these inequalities in a matrix H, then we have

$$\mathcal{C}_0 = \{\dot{\boldsymbol{c}} \mid H\dot{\boldsymbol{c}} \geq \boldsymbol{0}\}. \tag{48}$$

7.3.1 Dropping columns corresponding to internal metabolites

We see that the columns of H thus correspond to the metabolites in the metabolic network. We specify that we have $|\text{Ext}|$ external metabolites, $|\text{Int}|$ internal metabolites, and let us assume that H is ordered

such that all columns corresponding to external metabolites come first, i.e., $H = \begin{bmatrix} H_{\text{Ext}} & H_{\text{Int}} \end{bmatrix}$. To obtain an inequality description of the steady-state conversion cone, we should impose the steady-state constraints $\dot{c}_i = 0$ for $i \in Int$. This can be done easily by adding two inequality constraints per internal metabolites:

$$\mathcal{C} = \left\{ \dot{\boldsymbol{c}} \in \mathbb{R}^{|\text{Ext}|+|\text{Int}|} \mid H^{+}\dot{\boldsymbol{c}} \geq \boldsymbol{0} \right\}, \quad \text{where } H^{+} = \begin{bmatrix} H_{\text{Ext}} & H_{\text{Int}} \\ 0 & I_{|\text{Int}|} \\ 0 & -I_{|\text{Int}|} \end{bmatrix}, \tag{49}$$

with $I_{|\text{Int}|}$ being the ($|\text{Int}| \times |\text{Int}|$)-identity matrix. Although this is a valid description of $\mathcal{C}$, it is not a very efficient one. We prove in the following Theorem that we can just drop all columns corresponding to the internal metabolites.

Theorem 6. *Let $\mathcal{C} = \{\boldsymbol{x} \in \mathbb{R}^{|Ext|+|Int|} \mid H^{+}\boldsymbol{x} \geq \boldsymbol{0}\}$, and $\mathcal{C}' = \{\tilde{\boldsymbol{x}} \in \mathbb{R}^{|Ext|} \mid H_{Ext}\tilde{\boldsymbol{x}} \geq \boldsymbol{0}\}$. There is a bijection between cones $\mathcal{C}$ and $\mathcal{C}'$.*

Proof. Define a mapping between the two cones by keeping only the first $|\text{Ext}|$ coordinates:

$$\begin{aligned} f &: \mathcal{C} \to \mathcal{C}', \\ &: \boldsymbol{x} \mapsto \begin{bmatrix} x_1 & \cdots & x_{|\text{Ext}|} \end{bmatrix}^T. \end{aligned}$$

We first show that f indeed maps into $\mathcal{C}'$. Take an arbitrary element $\boldsymbol{x} \in \mathcal{C}$. We know that $H^{+}\boldsymbol{x} \geq \boldsymbol{0}$. The last rows imply that $0\boldsymbol{x}_{\text{Ext}} + I_{|\text{Int}|}\boldsymbol{x}_{\text{Int}} \geq \boldsymbol{0}$, and $0\boldsymbol{x}_{\text{Ext}} - I_{|\text{Int}|}\boldsymbol{x}_{\text{Int}} \geq 0$, so that we must have $\boldsymbol{x}_{\text{Int}} = \boldsymbol{0}$. The first rows of the inequalities then imply $\boldsymbol{0} \leq H_{\text{Ext}}\boldsymbol{x}_{\text{Ext}} - H_{\text{Int}}\boldsymbol{0} = H_{\text{Ext}}\boldsymbol{x}_{\text{Ext}}$, which means that $f(\boldsymbol{x}) = \boldsymbol{x}_{\text{Ext}} \in \mathcal{C}'$.

With the knowledge that for any $\boldsymbol{x} \in \mathcal{C}$, the components corresponding to internal metabolites have to be zero, we can define an inverse mapping

$$\begin{aligned} g &: \mathcal{C}' \to \mathcal{C}, \\ &: \tilde{\boldsymbol{x}} \mapsto \begin{bmatrix} \tilde{\boldsymbol{x}}^T & \boldsymbol{0}^T \end{bmatrix}^T. \end{aligned}$$

This mapping is clearly a left- and right-inverse of f, and therefore f must be a bijection. □

We can thus proceed with H_{Ext} as the inequality description of the steady-state conversion cone.

7.3.2 Splitting external metabolites

In Section 3.3, we explained why we need to split external metabolites into input- and output-metabolites in order to calculate all ECMs instead of only the extreme conversions. We also indicated that this splitting can be done during the preprocessing step. However, one can also choose (by using the argument `--splitting_before_polco False`) to calculate H_{Ext} without splitting the metabolites, and split the metabolites afterwards. This choice does affect the size of H_{Ext} and therefore the computational complexity of ECM-enumeration, but we could get no clear idea of when which method is favourable. We recommend the user to try both methods when working with large models, because we have observed that this option sometimes makes a large difference.

Let us now assume that the external metabolites are not yet split into input- and output-metabolites, and say, for simplicity, that all metabolites can both be consumed and produced, so that all need to be split. Then we can use H_{Ext} to define

$$H_{\text{split}} = \begin{bmatrix} H_{\text{Ext}} & H_{\text{Ext}} \\ I & 0 \\ 0 & -I \end{bmatrix}. \tag{50}$$

Now let us compare the cones that are described by these inequality representations:

$$\mathcal{C}_{\text{Ext}} = \{\boldsymbol{x} \in \mathbb{R}^{|\text{Ext}|} \mid H_{\text{Ext}}\boldsymbol{x} \geq \boldsymbol{0}\}, \tag{51}$$

$$\mathcal{C}_{\text{split}} = \{\tilde{\boldsymbol{x}} \in \mathbb{R}^{2|\text{Ext}|} \mid H_{\text{split}}\tilde{\boldsymbol{x}} \geq \boldsymbol{0}\}. \tag{52}$$

We can define a mapping f from $\mathcal{C}_{\text{Ext}}$ to $\mathcal{C}_{\text{split}}$ by

$$\begin{aligned} f &: \mathcal{C}_{\text{Ext}} \to \mathcal{C}_{\text{split}}, \\ &: \boldsymbol{x} \mapsto \tilde{\boldsymbol{x}} = \begin{bmatrix} \max(x_1, 0), \cdots, \max(x_{|\text{Ext}|}, 0), \min(x_1, 0), \cdots, \min(x_{|\text{Ext}|}, 0) \end{bmatrix}^T, \end{aligned}$$

We can show that this mapping is well-defined. Let $\boldsymbol{x} \in \mathcal{C}_{\text{Ext}}$, we have to check if $\tilde{\boldsymbol{x}}$ satisfies $H_{\text{split}}\tilde{\boldsymbol{x}} \geq 0$. First of all, it is clear from the definition of f that $\tilde{\boldsymbol{x}}$ satisfies the sign constraints that form the two lower rows of H_{split}. Further, the first rows of H_{split} give

$$\begin{aligned} H_{\text{split}}^{\text{top}}\tilde{\boldsymbol{x}} &= H_{\text{Ext}}\left[\max(x_1,0),\cdots,\max(x_{|\text{Ext}|},0)\right)^T + H_{\text{Ext}}\left(\min(x_1,0),\cdots,\min(x_{|\text{Ext}|},0)\right]^T \\ &= H_{\text{Ext}}\left[\max(x_1,0)+\min(x_1,0),\cdots,\max(x_{|\text{Ext}|},0)+\min(x_{|\text{Ext}|},0)\right]^T, \\ &= H_{\text{Ext}}\boldsymbol{x} \geq \boldsymbol{0}. \end{aligned}$$

This shows that any steady-state conversion in $\mathcal{C}_{\text{Ext}}$ is still a steady-state conversion in $\mathcal{C}_{\text{split}}$. The following Theorem shows that the ECMs of $\mathcal{C}_{\text{Ext}}$ can be calculated by computing the minimal generator set of $\mathcal{C}_{\text{split}}$.

Theorem 7. *There is a bijection between the set of Elementary Conversion Modes of $\mathcal{C}_{Ext}$ and the minimal generator set of $\mathcal{C}_{split}$.*

The proof of this theorem is very similar to the proof of Theorem 3 in Section 3.3. Therefore, we will not repeat it here.

If we compute a minimal generator in $\mathcal{C}_{\text{split}}$, we can thus map this back to an ECM in $\mathcal{C}_{\text{Ext}}$. For this, we use the left-inverse of f:

$$\begin{aligned} g &: C_{\text{split}} \to C_{\text{Ext}}, \\ &: \tilde{\boldsymbol{x}} \mapsto \boldsymbol{x} = \left[\tilde{x}_1 + \tilde{x}_{|\text{Ext}|+1},\ldots,\tilde{x}_{|\text{Ext}|} + \tilde{x}_{2|\text{Ext}|}\right]^T. \end{aligned}$$

7.3.3 Removing redundant inequalities

The inequality description of $\mathcal{C}_0$, gathered in the matrix H, does not contain redundant inequalities, because the `polco`-software returns a minimal set. However, we have used H to define H_{Ext} and then H_{split}. Often, and mostly when there are relatively many internal metabolite columns that are dropped, these operations cause inequalities to become redundant. In theory, this causes no problem for the next steps in the ECM-enumeration. However, when H_{split} has many rows, the next Double Description step is much slower. We can therefore speed up the ECM-computation by removing redundant rows. For this, we can use either the `redund`-program from `lrslib`[8] on small sets of rows, or our own algorithm for redundancy removal. Our algorithm has the advantages of being parallelizable if `ecmtool` is used with `mpiexec` (see the user guide in Section 11), and of reporting a counter which indicates the progress of the redundancy-removal. By default, we thus use our algorithm. We will shortly discuss how this works.

We call a row redundant if it can be written as a conical combination of other rows, i.e.,

$$\boldsymbol{A}_{i\bullet} = \sum_j \lambda_j \boldsymbol{A}_{j\bullet}, \quad \text{where } \lambda_j \geq 0. \tag{53}$$

This row is called redundant for the following reason. If for all j such that $\lambda_j > 0$ we have $\boldsymbol{A}_{j\bullet}\boldsymbol{x} \geq \boldsymbol{0}$, then automatically $\boldsymbol{A}_{i\bullet}\boldsymbol{x} = \sum_j \lambda_j \boldsymbol{A}_{j\bullet}\boldsymbol{x} \geq 0$. So, the inequality implied by $\boldsymbol{A}_{i\bullet}$ does not further constrain the cone. We can thus remove all rows for which we can find a conical combination as in Equation (53)[1]. We detect these redundant rows by solving a Linear Program for each row:

$$\begin{aligned} \underset{\boldsymbol{\lambda}}{\text{maximize}} \quad & \sum_{i\neq j} \lambda_i \\ \text{subject to} \quad & A^T\boldsymbol{\lambda} = \boldsymbol{A}_{i\bullet}^T \\ & \lambda_i \geq 0. \end{aligned} \tag{54}$$

This problem is exactly of the form described in Section 4, so we can use our custom LP-solver to solve it. In Section 4.5 we described that for this LP-solver to work, we need to select a maximal set of linearly independent columns of A^T as a starting basis. This basis should contain the column A_i^T that we want to test for redundancy. Finding such a starting basis is a relatively complex computational task. However,

[1]It is important that we first make sure that any duplicate rows are removed, since otherwise the procedure could remove both of them.

we use the same matrix A^T for each redundancy test, and we can therefore use almost the same starting basis. We should only make sure that we replace one of the columns in this basis by $\boldsymbol{A}_{i\bullet}^T$. In Section 4.5 we describe how we accomplish this.

It is important to note that this redundancy removal only works when the cone generated by the columns of A^T is pointed. If not, we use the strategy that we developed for the removal of cycles to make the cone pointed. We will very shortly discuss the strategy here too, but it is so similar to the removal of cycles that we refer to Section 5.5 for details.

If there is a $\mathbf{0} \neq \boldsymbol{\lambda} \geq \mathbf{0}$ such that $A^T\boldsymbol{\lambda} = \mathbf{0}$, then the Linear Program in (54) is unbounded. We solve this by selecting one $\lambda_j > 0$ and take the corresponding row $\boldsymbol{A}_{j\bullet}$. Since the rows of A correspond to inequalities, we can see that $\boldsymbol{A}_{j\bullet}\boldsymbol{x} \geq \mathbf{0}$ and $0 \leq \sum_{k\neq j} \lambda_k \boldsymbol{A}_{k\bullet}\boldsymbol{x} = -\lambda_j \boldsymbol{A}_{j\bullet}\boldsymbol{x}$, so that $\boldsymbol{A}_{j\bullet}$ in fact gives an equality constraint: $\boldsymbol{A}_{j\bullet}\boldsymbol{x} = 0$. This means that we can add or subtract $\boldsymbol{A}_{j\bullet}$ to other rows without affecting the inequality representation. We choose to pick a nonzero entry of $\boldsymbol{A}_{j\bullet}$, say A_{jl} and cancel the l-th entry from each row in A. The row $\boldsymbol{A}_{j\bullet}$ itself will for now be stored. We can repeat this procedure until the resulting cone is pointed. Then, we can use the redundancy-removal outlined above, and after that we add the found equality constraints again.

7.4 $\mathbf{ineq}(\mathcal{C}) \overset{\mathbf{DD}}{\to} \mathbf{gen}(\mathcal{C}) = \mathbf{ECMs}$

In this last step, we start with the found inequality representation of the steady-state conversion cone. Because we have split all metabolites, this cone is contained in one orthant. This automatically implies that the cone must be pointed. We can thus simply apply the Double Description method (again using fractions) to find a minimal generating set of the steady-state conversion cone. After undoing the splitting of external metabolites (as mentioned in Section 7.3.2) this yields the Elementary Conversion Modes, finally.

8 The direct method

The starting point of the direct method is the generator description of the (non-steady-state) conversion cone, $\mathcal{C}_0$, defined in (45) in Section 6. We assume that cycles have been removed using the method described in Section 5.5 such that this cone is pointed. The remaining task is thus to impose the steady-state constraints $\dot{c}_i = 0$ for all internal metabolites i. Whereas the indirect method computes an inequality representation of $\mathcal{C}_0$ before imposing the steady-state constraints, the direct method will impose these constraints 'directly' on the set of generators. As far as we know, this direct intersection method is unconventional. It has probably not been used before because it is usually slower than the indirect intersection method. It however avoids the (too) high memory usage that the indirect method sometimes suffers from.

The direct method proceeds by an iterative procedure. We start by gathering the generators from the generator description of $\mathcal{C}_0$ in a matrix $R^{(0)}$, and then impose an additional steady-state constraint in each iteration. We assume for now that we have ordered the metabolites such that there are K internal metabolites with indices $\{1, \ldots, K\}$. In the first step, we impose the constraint $\dot{c}_1 = 0$. The result is a new cone, called $\mathcal{C}^{(1)}$ which again has a set of generators, which we gather in the matrix $R^{(1)}$. We continue to add the constraints until we end up with the generators gathered in $R^{(K)}$ of the steady-state conversion cone: $\mathcal{C} = \mathcal{C}^{(K)}$.

In the following we will denote by $\boldsymbol{r}_j \in R$ the j-th column of the matrix R.

8.1 Imposing one steady-state constraint

The i-th iteration of the algorithm starts with the matrix $R^{(i-1)}$ of non-redundant columns. These columns can be interpreted as conversions that satisfy the steady-state constraints for all metabolites with index smaller than i. The entry $R_{ij}^{(i-1)}$ thus indicates the net production of metabolite i in the j-th conversion. During this step, we will impose the constraint $\dot{c}_i = 0$, which thus means that we should make sure that $R_{ij}^{(i)} = 0$ for all j. For this, similar to the Double Description method (Section 1.5), we

compute the index sets

$$\begin{aligned} J^+ &= \{j \in \{1,\dots,n\} : R_{ij}^{(i-1)} > 0\}, \\ J^- &= \{j \in \{1,\dots,n\} : R_{ij}^{(i-1)} < 0\}, \\ J^0 &= \{j \in \{1,\dots,n\} : R_{ij}^{(i-1)} = 0\}. \end{aligned} \tag{55}$$

We are certain that the vectors corresponding to J^0 will be minimal generators of $\mathcal{C}^{(i)}$: they satisfy the constraint $\dot{c}_i = 0$ and cannot be written as a conical combination of the other vectors, since otherwise they would not have been in $R^{(i-1)}$. Furthermore, each pair of indices $j_+ \in J^+$, $j_- \in J^-$ with their corresponding vectors $\boldsymbol{r}_{j_+}, \boldsymbol{r}_{j_-}$ generate a possible candidate: $\hat{\boldsymbol{r}} = R_{ij_+}^{(i-1)} \boldsymbol{r}_{j_-}^{(i-1)} - R_{ij_-}^{(i-1)} \boldsymbol{r}_{j_+}$. We do not have to consider combinations of more vectors, since these can always be written as combinations of the pairs.

We could get a generating set of $\mathcal{C}^{(i)}$ by taking the union of all $\hat{\boldsymbol{r}}$ with the vectors from J^0, but this set would not be minimal. Therefore, we need a test to determine which candidates to keep, analogous to the adjacency test that is used in the Double Description method, Section 1.4. The adjacency test of the Double Description method however uses information about which inequalities are satisfied with equality by the generators to determine if a pair is adjacent or not. Since we intersect with equalities instead of inequalities, we cannot directly use this method. We would like to find an alternative way to determine the adjacency of two rays, using only the ray representation R.

The next section provides this alternative adjacency test. In short: we test whether two rays are adjacent by considering a point in between them. If this point can also be formed by a conic combination that includes other rays than the original two, then they are not adjacent.

8.1.1 A geometric adjacency test

Let

$$\boldsymbol{x} = \frac{1}{4}\boldsymbol{r}_{j_+} + \frac{3}{4}\boldsymbol{r}_{j_-}$$

and consider the linear program, $\text{LP}(\boldsymbol{r}_{j_+}, \boldsymbol{r}_{j_-})$:

$$\begin{aligned} \underset{\boldsymbol{\lambda}}{\text{maximize}} \quad & \sum_{j \notin \{j_+, j_-\}} \lambda_i \\ \text{subject to} \quad & R\boldsymbol{\lambda} = \boldsymbol{x} \\ & \lambda_i \geq 0. \end{aligned} \tag{56}$$

Note that the LP is always feasible, and has optimal value at least 0, since we have the initial solution $\bar{\boldsymbol{\lambda}}$ with $\bar{\lambda}_{j_+} = 1/4$, $\bar{\lambda}_{j_-} = 3/4$ and zero in the other coordinates. We will prove in the following theorem that this LP can be used as an adjacency test. The reason for choosing 1/4 and 3/4 is that this ascertains that the 'target' $\boldsymbol{x}$ is not close to the zero vector. In fact, in `ecmtool` we normalize the rays before starting the LP such that the L^1-norm of all rays is equal to 1. We can then show with the reverse triangle inequality that

$$||\boldsymbol{x}|| = \left|\left|\frac{1}{4}\boldsymbol{r}_{j_+} - (-\frac{3}{4}\boldsymbol{r}_{j_-})\right|\right| \geq \left|\frac{1}{4}||\boldsymbol{r}_{j_+}|| - \frac{3}{4}||\boldsymbol{r}_{j_-}||\right| = \left|-\frac{1}{2}\right| = \frac{1}{2}. \tag{57}$$

In the following we will denote by R the matrix of generators (columns $\boldsymbol{r}_j$), and by A the inequality matrix of the same polyhedral cone $P(A)$. Recall from Section 1.4 that the zero set of a generator was defined as

$$Z(\boldsymbol{r}_j) = \{i : \boldsymbol{A}_{i\bullet}\boldsymbol{r}_j = 0\}.$$

Theorem 8 (geometric adjacency test)**.** *The following are equivalent for extreme rays $\boldsymbol{r}_{j_+}, \boldsymbol{r}_{j_-}$:*
(1) $\boldsymbol{r}_{j_+}$ and $\boldsymbol{r}_{j_-}$ are adjacent.
(2) $Z(\boldsymbol{r}_{j_+}) \cap Z(\boldsymbol{r}_{j_-}) \subseteq Z(\boldsymbol{r}_k) \implies \boldsymbol{r}_k \sim \boldsymbol{r}_{j_+}$ or $\boldsymbol{r}_k \sim \boldsymbol{r}_{j_-}$.
(3) $LP(\boldsymbol{r}_{j_+}, \boldsymbol{r}_{j_-})$ has optimal value 0.

Proof. The equivalence of (1) and (2) is just the definition of adjacency described in Section 1.4.

(2) $\implies$ **(3)**. Suppose (2) holds, so $Z(\boldsymbol{r}_{j_+}) \cap Z(\boldsymbol{r}_{j_-}) \subseteq Z(\boldsymbol{r}_k) \implies \boldsymbol{r}_k \sim \boldsymbol{r}_{j_+}$ or $\boldsymbol{r}_k \sim \boldsymbol{r}_{j_-}$. Consider any extreme ray $\boldsymbol{r}_k$ that is not equivalent to $\boldsymbol{r}_{j_+}$ or $\boldsymbol{r}_{j_-}$. We know that $Z(\boldsymbol{r}_k)$ does not contain $Z(\boldsymbol{r}_{j_+}) \cap Z(\boldsymbol{r}_{j_-})$, so there is some index $i \in Z(\boldsymbol{r}_{j_+}) \cap Z(\boldsymbol{r}_{j_-})$ that is not in $Z(\boldsymbol{r}_k)$. This means $\boldsymbol{A}_{i\bullet}\boldsymbol{r}_k > 0$.

Suppose $\boldsymbol{\lambda}$ is a feasible solution for $\mathrm{LP}(\boldsymbol{r}_{j_+}, \boldsymbol{r}_{j_-})$, so $R\boldsymbol{\lambda} = \boldsymbol{x}$. Now consider

$$\boldsymbol{A}_{i\bullet}R\boldsymbol{\lambda} = \sum_j \boldsymbol{A}_{i\bullet}\boldsymbol{r}_j\lambda_j \geq \boldsymbol{A}_{i\bullet}\boldsymbol{r}_k\lambda_k. \tag{58}$$

The last inequality holds because each ray $\boldsymbol{r}_j$ is in the cone; hence $\boldsymbol{A}_{i\bullet}\boldsymbol{r}_j \geq 0$, and also $\lambda_j \geq 0$ (one of the LP constraints). At the same time

$$\boldsymbol{A}_{i\bullet}R\boldsymbol{\lambda} = \boldsymbol{A}_{i\bullet}\boldsymbol{x} = \boldsymbol{A}_{i\bullet}(\frac{1}{4}\boldsymbol{r}_{j_+} + \frac{3}{4}\boldsymbol{r}_{j_-}) = 0, \tag{59}$$

since $i \in Z(\boldsymbol{r}_{j_+})$ and $i \in Z(\boldsymbol{r}_{j_-})$. Combining (58) and (59) with $\boldsymbol{A}_{i\bullet}\boldsymbol{r}_k > 0$ gives $\lambda_k = 0$. Because $\boldsymbol{r}_k$ was any extreme ray not equivalent to $\boldsymbol{r}_{j_+}$ or $\boldsymbol{r}_{j_-}$, and $\boldsymbol{\lambda}$ was any feasible solution, this means that we will always have $\sum_i \lambda_i - \lambda_a - \lambda_b = 0$, hence (3) holds.

(3) $\implies$ **(2)**. For a contrapositive proof, assume (2) does not hold. Then there is some ray $\boldsymbol{r}_k$ such that $Z(\boldsymbol{r}_{j_+}) \cap Z(\boldsymbol{r}_{j_-}) \subseteq Z(\boldsymbol{r}_k)$. By definition $\boldsymbol{x} = 1/4\boldsymbol{r}_{j_+} + 3/4\boldsymbol{r}_{j_-}$. Therefore, for any row $\boldsymbol{A}_{i\bullet}$,

$$\boldsymbol{A}_{i\bullet}\boldsymbol{x} = \boldsymbol{A}_{i\bullet}(\frac{1}{4}\boldsymbol{r}_{j_+} + \frac{3}{4}\boldsymbol{r}_{j_-}) = \frac{1}{4}\boldsymbol{A}_{i\bullet}\boldsymbol{r}_{j_+} + \frac{3}{4}\boldsymbol{A}_{i\bullet}\boldsymbol{r}_{j_-}. \tag{60}$$

Because $\boldsymbol{r}_{j_+}$ and $\boldsymbol{r}_{j_-}$ are in the cone, $\boldsymbol{A}_{i\bullet}\boldsymbol{r}_{j_+} \geq 0$ and $\boldsymbol{A}_{i\bullet}\boldsymbol{r}_{j_-} \geq 0$. Thus $\boldsymbol{A}_{i\bullet}x = 0$ if and only if $\boldsymbol{A}_{i\bullet}\boldsymbol{r}_{j_+} = \boldsymbol{A}_{i\bullet}\boldsymbol{r}_{j_-} = 0$, hence

$$Z(\boldsymbol{x}) = Z(\boldsymbol{r}_{j_+}) \cap Z(\boldsymbol{r}_{j_-}). \tag{61}$$

Consider the line segment

$$L(t) = t\boldsymbol{x} + (1-t)\boldsymbol{r}_k \tag{62}$$

for $t \in [0, 1]$. This is the line segment from $\boldsymbol{r}_k$ to $\boldsymbol{x}$. In Figure S8 we have schematically drawn the situation. Take any index l not in $Z(\boldsymbol{x})$. Since $\boldsymbol{A}_{l\bullet}\boldsymbol{x} > 0$, we have, for any $t \in (0, 1]$,

$$\boldsymbol{A}_{l\bullet}L(t) = \boldsymbol{A}_{l\bullet}t\boldsymbol{x} + \boldsymbol{A}_{l\bullet}(1-t)\boldsymbol{r}_k > 0. \tag{63}$$

Because $\boldsymbol{A}_{l\bullet}L(t)$ is continuous with respect to t, there is an $\varepsilon_l > 0$ such that $\boldsymbol{A}_{l\bullet}L(1+\varepsilon_l) > 0$. Define ε as the minimum of all the ε_l obtained in this way, then

$$\boldsymbol{A}_{l\bullet}L(1+\varepsilon) > 0 \text{ for all } l \text{ not in } Z(\boldsymbol{x}) \tag{64}$$

On the other hand, any index $m \in Z(\boldsymbol{x})$ is also in $Z(\boldsymbol{r}_k)$, since $Z(\boldsymbol{x}) = Z(\boldsymbol{r}_{j_+}) \cap Z(\boldsymbol{r}_{j_-}) \subseteq Z(\boldsymbol{r}_k)$. This yields

$$\boldsymbol{A}_{m\bullet}L(1+\varepsilon) = \boldsymbol{A}_{m\bullet}(1+\varepsilon)\boldsymbol{x} - \boldsymbol{A}_{m\bullet}\varepsilon\boldsymbol{r}_k = 0 \text{ for all } m \in Z(\boldsymbol{x}). \tag{65}$$

Let $\boldsymbol{\alpha} = L(1+\varepsilon)$, it follows from (64) and (65) that $\boldsymbol{\alpha} \in P(A)$ and $Z(\boldsymbol{\alpha}) = Z(\boldsymbol{x})$. We can write

$$\boldsymbol{\alpha} = L(1+\varepsilon) = (1+\varepsilon)\boldsymbol{x} - \varepsilon\boldsymbol{r}_k, \tag{66}$$

so that

$$\boldsymbol{\alpha} + \varepsilon\boldsymbol{r}_k = (1+\varepsilon)\boldsymbol{x}, \tag{67}$$

and

$$\frac{\boldsymbol{\alpha}}{1+\varepsilon} + \frac{\varepsilon\boldsymbol{r}_k}{1+\varepsilon} = \boldsymbol{x}. \tag{68}$$

Because $\boldsymbol{\alpha}$ is a point in $P(A)$, it can be written as a conic combination of rays, say $\boldsymbol{\alpha} = \sum_i \lambda_i\boldsymbol{r}_i$. But then $\frac{1}{1+\varepsilon}\boldsymbol{\lambda} + \frac{\varepsilon}{1+\varepsilon}\hat{\boldsymbol{e}}_k$ is a feasible solution to the LP in (56), with objective value

$$\sum_{i \notin j_+, j_-} \frac{1}{1+\varepsilon}\lambda_i + \frac{\varepsilon}{1+\varepsilon} \geq \frac{\varepsilon}{1+\varepsilon} > 0. \tag{69}$$

So, $\mathrm{LP}(\boldsymbol{r}_{j_+}, \boldsymbol{r}_{j_-})$ does not have optimal value 0. By contrapositivity, this implies that whenever the optimal value is 0, $\boldsymbol{r}_{j_+}$ and $\boldsymbol{r}_{j_-}$ must be adjacent. □

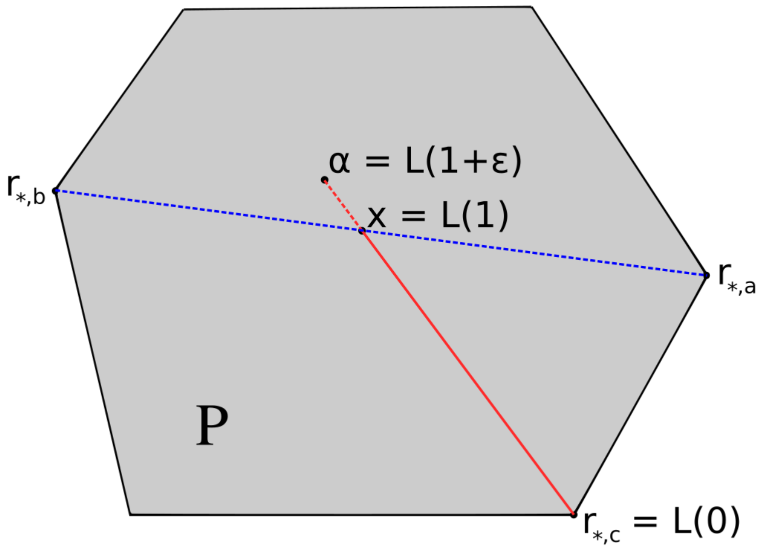

Figure S8: Illustration for the proof of **(3)** $\implies$ **(2)**. The point $\boldsymbol{\alpha}$ will always be inside P, indicating a feasible solution to the LP with objective strictly greater than 0. The grey area might not look like a cone at first, but consider it as a cross section of one.

This theorem showed that solving the right LP can serve as an adjacency test for extreme rays. The following theorem in addition shows that if the rays are not adjacent, the original rays cannot be part of the optimal solution to the LP. To be precise: if the rays $\boldsymbol{r}_{j_+}, \boldsymbol{r}_{j_-}$ are not adjacent, then at some point the contribution λ_{j_+} or λ_{J_-} must become zero. This is important to make the adjacency test numerically robust, because the change in $\boldsymbol{\lambda}$ has to be of size at least 1/4 which means that it is easy to distinguish from round-off errors. Moreover, it provides an early exit strategy if we use our LP-solver described in Section 4: at the point that λ_{j_+} or λ_{j_-} becomes zero, we know that the rays are non-adjacent, and we can stop the LP.

Theorem 9. *Let $\boldsymbol{\mu}$ be the optimal solution to LP($\boldsymbol{r}_{j_+}, \boldsymbol{r}_{j_-}$). Then at least one of μ_{j_+} and μ_{j_-} is equal to zero.*

Proof. Suppose that both $\mu_{j_+} > 0$ and $\mu_{j_-} > 0$. We will show that this contradicts the optimality of $\boldsymbol{\mu}$. Besides $\boldsymbol{\mu}$ we know of one more feasible solution: the vector $\boldsymbol{\nu}$ with all zeroes except for $\nu_{j_+} = 1/4$ and $\nu_{j_-} = 3/4$. Since both $\boldsymbol{\mu}$ and $\boldsymbol{\nu}$ are feasible solutions, we have $R(\boldsymbol{\mu} - \boldsymbol{\nu}) = 0$. Now consider $\bar{\boldsymbol{\mu}} = \boldsymbol{\mu} + \delta(\boldsymbol{\mu} - \boldsymbol{\nu})$ for some small $\delta > 0$. This $\bar{\boldsymbol{\mu}}$ satisfies

$$R\bar{\boldsymbol{\mu}} = R(\boldsymbol{\mu} + \delta(\boldsymbol{\mu} - \boldsymbol{\nu})) = R\boldsymbol{\mu} + \delta R(\boldsymbol{\mu} - \boldsymbol{\nu}) = R\boldsymbol{\mu} + \mathbf{0} = \boldsymbol{x}. \tag{70}$$

The only positions in $(\boldsymbol{\mu} - \boldsymbol{\nu})$ that could be negative are j_+ and j_-, since $\boldsymbol{\nu}$ is zero everywhere else. But we know that $\mu_{j_+}, \mu_{j_-} > 0$, so if we pick δ small enough then $\bar{\mu}_{j_+}, \bar{\mu}_{j_-} \geq 0$, so that

$$\bar{\boldsymbol{\mu}} \geq \mathbf{0}. \tag{71}$$

Together (70) and (71) show that $\bar{\boldsymbol{\mu}}$ is a feasible solution. Now let us denote by Obj($\boldsymbol{\mu}$) the objective value corresponding to the solution $\boldsymbol{\mu}$. We can see that

$$\text{Obj}(\bar{\boldsymbol{\mu}}) = \sum_{i \notin \{j_+, j_-\}} \bar{\mu}_i = \sum_{i \notin \{j_+, j_-\}} \mu_i + \delta(\mu_i - \nu_i) = (1+\delta) \sum_{i \notin \{j_+, j_-\}} \mu_i > \sum_{i \notin \{j_+, j_-\}} \mu_i = \text{Obj}(\boldsymbol{\mu}). \tag{72}$$

This would contradict the optimality of $\boldsymbol{\mu}$, so it must be that in the optimal solution $\mu_{j_+} = 0$ or $\mu_{j_-} = 0$. □

8.1.2 Performing the adjacency tests

Recall that one iteration of the direct method involves finding a minimal generating set of the cone $\mathcal{C}^{(i)}$ from the generating set $R^{(i-1)}$ of $\mathcal{C}^{(i-1)}$. For that, we determined for all generating vectors in $R^{(i-1)}$ the sign of $\dot{c}_i$, and used that to construct the sets J^+, J^-, J^0 (see (55)). All pairs with one ray from J^+ and one ray from J^- give a potential candidate that we should test for redundancy. The candidate will only be non-redundant if the original rays $\boldsymbol{r}_{j_+}, \boldsymbol{r}_{j_-}$ are adjacent, according to the adjacency test defined above. To test all these candidates, we thus have to perform $|J^+||J^-|$ Linear Programs.

Fortunately, this LP is exactly of the form described in Section 4. We thus already have an efficient way to solve the LP. For this LP-solver to work, we do need to select a maximal set of linearly independent columns of R as a starting basis. This basis should contain the rays $\boldsymbol{r}_{j_+}, \boldsymbol{r}_{j_-}$ that constitute an initial solution. Finding this starting basis is computationally relatively complex. Note however, that we use the same matrix R for each redundancy test, and we can therefore use almost the same starting basis, B. We should only make sure that we replace two of the columns in A_B by $\boldsymbol{r}_{j_+}, \boldsymbol{r}_{j_-}$. In Section 4.5 we describe how we can add one such column. For this we need to solve $A_B\boldsymbol{x} = \boldsymbol{r}_{j_+}$. Call the basis where this column is added A_B^+. Now, we should still add the second column, and for that solve $A_B^+\boldsymbol{x} = \boldsymbol{r}_{j_-}$.

In `ecmtool` we optimize this even further. We start by selecting a basis matrix A_B once, and immediately calculate its LU-decomposition. This LU-decomposition can be used to solve $A_B\boldsymbol{x} = \boldsymbol{r}_{j_+}$. We solve this system for all possible $\boldsymbol{r}_{j_+}$, giving a set of bases A_B^+. For each basis in this set we also calculate the LU-decomposition once. These can then be used to solve the system $A_B^+\boldsymbol{x} = \boldsymbol{r}_{j_-}$ for all possible $\boldsymbol{r}_{j_-}$, yielding all the bases that we will need. This strategy requires us to calculate $1 + |J^+|$ LU-decompositions, instead of $|J^+||J^-|$, which induces a relevant computational speedup.

Another important optimization that decreases the computational time needed by the direct method, is that we perform the above described LPs using floats instead of fractions. We can do this because each LP will only give a boolean output, indicating if a pair of rays is adjacent, or not. We use fractions again when the adjacent rays are combined to form a generator that satisfies the steady-state constraints, so that the eventual ECM-computation remains exact.

8.2 The order of imposing steady-state constraints

As mentioned above, in each iteration we impose one of the steady-state constraints $\dot{c}_i = 0$. The order in which these constraints are imposed has a large effect on the total computation time. This is similar to the sensitivity of the Double Description method to changing the order in which inequalities are added.[6] It is however unclear which order of equality intersection minimizes the computation time. Therefore, we offer several heuristics as options using the argument `--sort_order`.

The sorting order that usually performs well is `--sort_order min_adj`. Here, we first sum how many metabolites are adjacent in the metabolic network, i.e., connected by one reaction. Then, for each i, we sum how many metabolites would become adjacent if we would connect all reactions that produce metabolite i to the reactions that consume metabolite i. As such, we try to estimate the number of adjacencies that are added, i.e., the difference between the number of adjacent metabolites after and before the removal of metabolite i. Note that the number of added adjacencies can be negative, because metabolite i is deleted and can thus no longer be adjacent to any metabolite. If this sorting order is chosen, `ecmtool` picks the metabolite that minimizes the number of added adjacencies. The intuition behind this sort order is that it would lead to summarizing possible modules in the metabolic network before connecting these to the rest of the network. Indeed, if we use this sorting order, the last steps are often not the largest.

An alternative heuristic that `ecmtool` offers is `--sort_order min_lp`. This selects the metabolite i that requires the minimal number of LPs. Recall that to impose the i-th steady-state constraint, we need to solve $|J^+||J^-|$ Linear Programs, where the size of the index sets J^+, J^- is given by how many of the current generators produce/consume metabolite i (see 55). This heuristic thus selects metabolites that are produced and consumed by few reactions. This has the disadvantage of removing the 'easy' metabolites first from the network, which could lead to a very difficult step later on.

8.3 Parallelization

Since the adjacency tests are independent of each other, and we commonly need to perform millions of them to eliminate a single metabolite (for genome-scale networks) this algorithm is highly suitable for parallel processing. We have implemented this using `mpi4py`.[15]

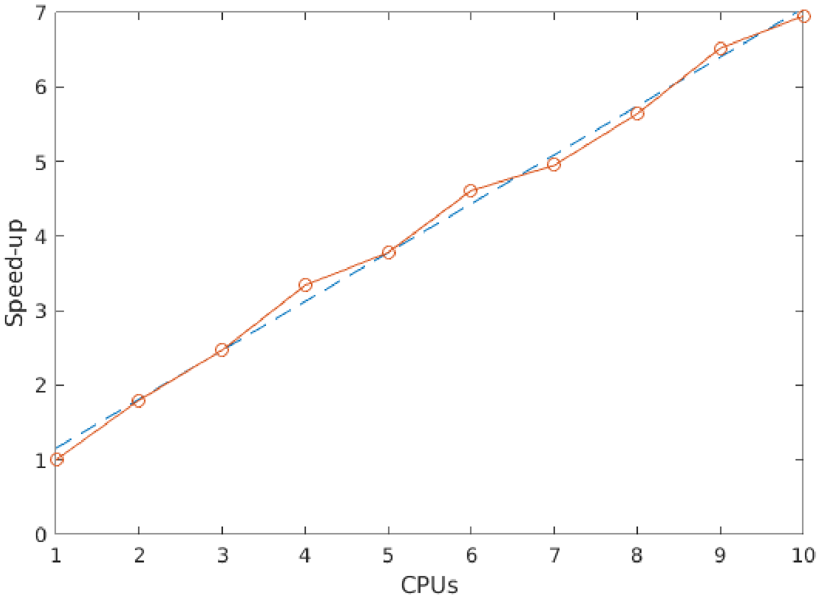

Figure S9: CPU count vs speed-up (red) and linear least-squares fit (blue, dashed). The fit is $y = 0.655x + 0.503$.

In Table 1 and Figure S9 we show the relative speed-up compared to using a single CPU for the `e_coli_core`-model. It shows close to linear gains. This is a smaller network, where the most LPs done in a single step is around $10e5$, so for genome scale networks we can expect the scaling to continue up to hundreds or more CPUs.

Table 1: Speed-up with different processor counts for *E. coli* core

**CPUs**	2	3	4	5	6	7	8	9	10
**Speed-up rel. to 1 CPU**	1.80	2.47	3.34	3.78	4.61	4.95	5.64	6.52	6.95

9 Validation of `ecmtool`

In this section, we describe how we used a `Matlab`-script to validate that the ECMs calculated by `ecmtool` for the `e_coli_core`-network satisfy

1. each ECM is an elementary vector
2. each steady-state conversion must be a conical combination of ECMs

1. According to the definition of ECMs given in Section 2, we can prove that each ECM is elementary by showing that it cannot be written as a positive sum of the other ECMs without the production of any external metabolite being cancelled. We tested this with the `Matlab`-script `lp_ecms_efm.m`, which is available as a Supplementary File.

For each ECM, $\boldsymbol{x}$, we first gather all other ECMs that are in the same orthant as columns in a matrix R. Then, we solve the following LP:

$$\begin{aligned} \underset{\boldsymbol{\lambda}}{\text{minimize}} \quad & \sum_i \lambda_i \\ \text{subject to} \quad & \begin{bmatrix} R \\ -R \end{bmatrix} \boldsymbol{\lambda} = \begin{bmatrix} \boldsymbol{x} + tol \\ -\boldsymbol{x} + tol \end{bmatrix} \\ & 0 \leq \lambda_i \leq 10^3. \end{aligned} \tag{73}$$

If the ECM is indeed an elementary vector, this LP should be infeasible. However, since the LP in `Matlab` is solved using floats, we should pay attention if round-off errors do not lead to incorrect results. Therefore, we allow for the decomposition to be off by a certain tolerance tol. If we allowed for an error of 10^{-7} none of the ECMs could be decomposed, indicating that they are all elementary. However, when we allowed for a larger error, more ECMs could be decomposed. This gives an indication of how close a conical combination of different ECMs can come to replacing an ECM. In Figure S10 we show the distribution of error margins that are needed to decompose an ECM into different ECMs. We see that most of the ECMs cannot be written as a combination of others unless one allows for an error of 10^{-5} or larger. This indicates that the ECMs are indeed all elementary vectors.

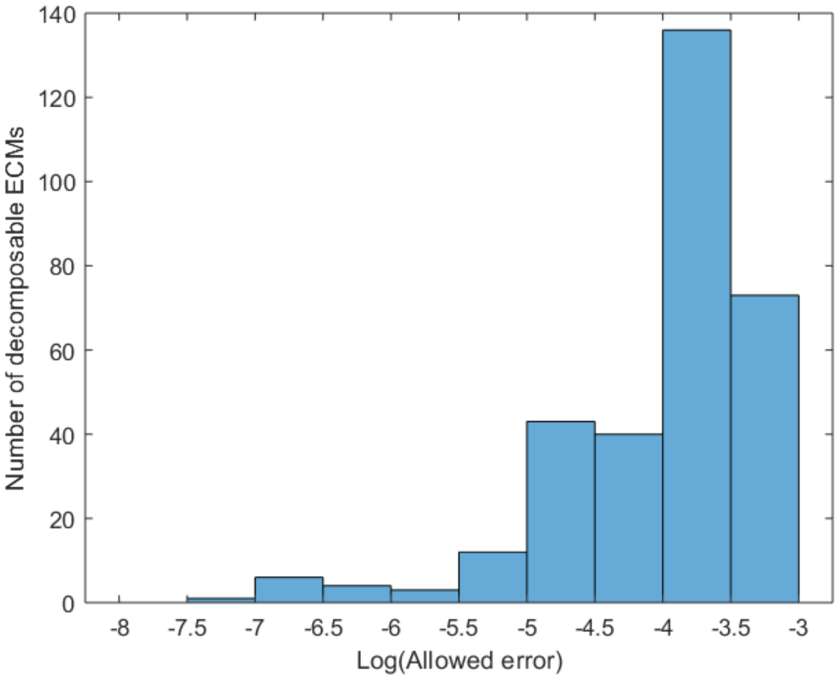

Figure S10: The distribution of error margins, tol that are needed to decompose an ECM into different ECMs.

2. It is of course hard to validate that any steady-sate conversion is a conical combination of ECMs. We therefore chose to use the set of Elementary Flux Modes calculated by `efmtool`. This set spans all possible steady-state flux combinations that the model contains. For each EFM, we then calculated its overall conversion, and tried to write this conversion as a combination of ECMs, using a similar LP as in (73). If we allowed for an error of 10^{-7}, then each EFM-based-conversion could be decomposed into ECMs. This error margin was necessary because the results from `efmtool` were affected by round-off errors, while the ECMs are calculated using fractions and are therefore exact.

When we multiply the EFMs with the stoichiometry matrix, we map them into the conversion cone.

Many of the EFMs will end up in the interior of the cone, thus leading to conversions that are combinations of ECMs. However, for each ECM there must be at least one EFM that leads to exactly that conversion. As a last sanity check, we tried to validate this. We took the decompositions of EFMs into ECMs obtained in the LPs above, and checked if each ECM occurs at least once as the sole decomposing vector of an EFM. We say that an ECM is a decomposing vector of an EFM if it is used more than some 'support tolerance'. In Figure S11, we vary this tolerance. We see that at a support tolerance of 10^{-2}, all ECMs have at least one EFM of which they are the sole decomposing ECM. Since both ECMs and EFMs were normalized before this validation, this implies that for all ECMs there is an EFM-based-conversion that is 99% equal to the ECM. This indicates that this EFM actually corresponds to the same conversion as the ECM; the 1% is caused by round-off errors and the allowed tolerance *tol* in the LP.

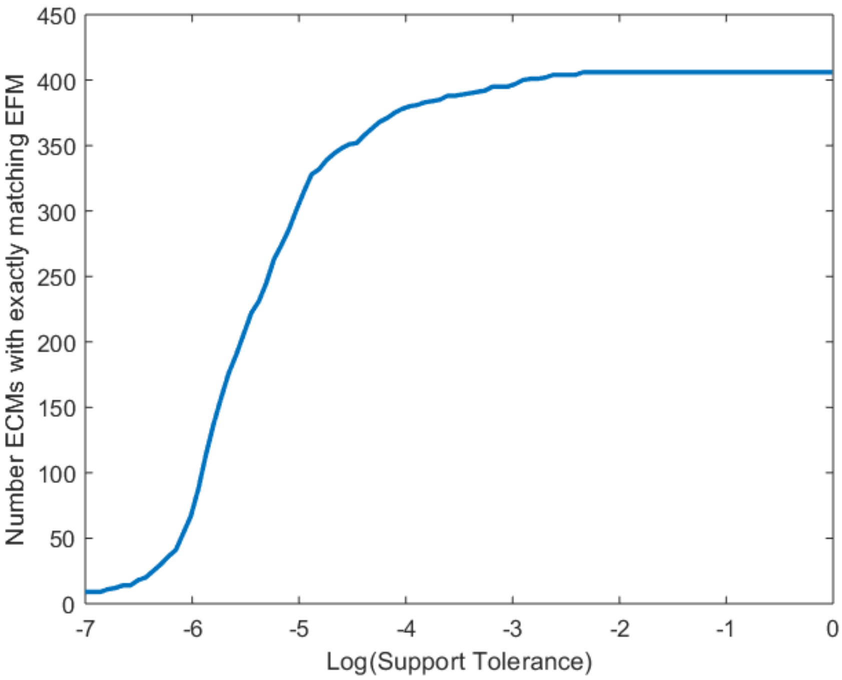

Figure S11: The cumulative number of ECMs that are identified as the sole decomposing ECM of at least one EFM, when we vary the cut-off at which we mark an ECM as 'decomposing'.

1. Description of validation on the `e_coli_core`-network
2. Figures of error distribution in decomposing EFMs

10 ECM-analyses several networks

In the main text we report on ECM computations for the `e_coli_core`-network,[16] the `iIT341`-network,[1] and the `iJR904`-network.[17] The full results and the run scripts that were used to obtain these results have been uploaded as supplementary files, and can also be found in the Github-folder: `https://github.com/SystemsBioinformatics/ecmtool`, specifically in the subfolder `results_and_corresponding_runscripts/`.

The Elementary Conversion Modes for the rhizobial bacteroids were calculated on the model `iCS320` by Schulte et al. The results and run scripts are reported in their work.[18]

10.1 Creating subnetworks of `e_coli_core`

In one of the figures of the main text we describe how the number of ECMs and EFMs behaves for various subnetworks of the `e_coli_core`-model. These subnetworks were created with a Python-script that we called `subnetwork_creator.py`, which is attached as a supplementary file. We created the subnetworks via an iterative procedure. The smallest subnetwork is constructed by taking only the active reactions in the FBA-solution. After that, we made a series of knockout-models. We deleted one of the active reactions, and again ran a Flux Balance Analysis. This knockout-model necessarily had a new set of active reactions. We took the union of these active reactions with the original active reactions to create our second model. Then, we again deleted a reaction, did another FBA, and took the active reactions. As such, we created subnetworks of increasing size for which we could compute the ECMs.

10.2 Clustering the ECM results for visualisation

We have clustered some of the ECM-enumeration results for visualization purposes. All `R`-scripts are made available as supplementary files. We first made sure that the set of ECMs was no larger than a few thousand. For some models, comprising hundreds of thousands ECMs, we had to take a subset of ECMs with a certain property, for example growth-supporting ECMs. Given this set, we created a distance matrix that contains the L^1-distance between all pairs of ECMs. For some models, we chose to weigh the L^1-distance so that some metabolites are considered more important than others. On this distance matrix, we performed hierarchical clustering. The metabolites were ordered from top to bottom by the number of ECMs that used the metabolite as an output minus the number of ECMs that used the metabolite as an input.

To visualize the clustered ECMs we used two options. For some models we converted all coefficients to a ternary scale, showing only whether the metabolite in the ECM was taken up, left untouched, or secreted. For other models we used a shifted logarithmic scale. To be precise, we converted the stoichiometric coefficients according to:

$$x = \begin{cases} \log\left(\frac{x}{shift_{pos} \cdot minpos}\right) & \text{if } x > 0, \\ 0 & \text{if } x = 0, \\ -\log\left(\frac{x}{shift_{neg} \cdot maxneg}\right) & \text{if } x < 0, \end{cases} \tag{74}$$

where $shift_{neg}, shift_{pos}$ are parameters smaller than 1, and $minpos, maxneg$ are respectively the smallest positive and the largest negative coefficient occurring in the ECMs. This transformation was necessary to visualize all differences in the coefficients occuring in the ECMs, because these coefficients span many orders of magnitudes and are both positive and negative. However, we should emphasize that this transformation can be used for visualization purposes only.

11 User guide

11.1 Prerequisite ingredients for ECM-computation

To compute the ECMs, one needs to provide at least an SBML-model. From the SBML-model, the following will be extracted by `ecmtool`

1. a stoichiometry matrix,
2. reversibility information of all reactions,
3. information on which metabolites are external or internal,
4. information on whether external metabolites can be produced, consumed or both

Important notes for the correct parsing of SBML-files by ecmtool

It should be checked carefully that `ecmtool` has parsed the model corresponding to the user's intentions. By far the most issues that users may have with ecmtool are **due to incorrect parsing of the SBML-file**. Because several conventions exist for storing several features of the model, `ecmtool` cannot comply

with all of them. When `ecmtool` is used as a standalone command line tool the parsing result can be checked by running `ecmtool` with the arguments `--print_reactions True` and `--print_metabolites True`, as described in subsection 11.2.1 below. When `ecmtool` is used as a Python library, the parsing result is available in the variable of the `network` class. Important to check are at least:

1. **reversibility information of all reactions**. We use the convention that reactions that are marked as irreversible can *only run in the forward direction*. Reactions can thus *not be backward irreversible*. In this case, the direction of the reaction should be swapped.

2. **internal/external-information of metabolites**. We use the convention that the metabolite-IDs of external metabolites are marked by `_e`. The user can change this with the argument `--external_compartment`. In addition, exchange reactions and external metabolites are recognized using functionality from the `cbmpy`-library, but this might not catch all.

3. **directionality information of external metabolites**. Based on the direction and reversibility of exchange reactions we determine whether a metabolite can be used as an input, an output or as both. This is what is most often parsed erroneously, due to conflicting conventions about when to set a reaction as reversible/irreversible in relation to its flux bounds.

`Ecmtool` can be used in two different modes: either as a standalone command line tool, or as a Python library for your own scripts. This section describes how to install and use both modes.

11.2 Mode 1: standalone command line tool

In this mode, you can call ecmtool like a normal program from your command line. It reads metabolic networks in the SBML format, and writes resulting ECMs into a CSV file for later analysis. Most researchers will use this method. For running `ecmtool` on computing clusters efficiently, see the Advanced Usage section in this readme.

Installation

- Download and install Python. `Ecmtool` is compatible with python 3.x. Ensure both python and its package manager *pip* are added to your PATH environment variable. If this last step is omitted, an error like the following will be thrown when you try to run python: *'python' is not recognized as an internal or external command [..].*

- Download the latest `ecmtool` source through *git clone*, or as a zip file from `https://github.com/tjclement/ecmtool`.

- Open a command prompt, and navigate to the ecmtool directory (e.g. *cd C:\Users\You\Git\ecmtool*, where the path should be replaced with the path `ecmtool` was downloaded to).

- Install the dependencies in requirements.txt inside the ecmtool directory (e.g. by running *pip install -r requirements.txt*).

- Linux only: install *redund* of package `lrslib` (e.g. by running *apt install lrslib*).

Running

`Ecmtool` can be ran by executing

```
python3 main.py {model_path <path/to/model.xml> [arguments]
```

from the command line, after navigating to the ecmtool directory as described above. The possible arguments and their default values are printed when you run `python main.py --help`. After execution is done, the found conversions have been written to file (default: *conversions.csv*). The first row of this CSV file contain the metabolite IDs as read from the SBML model.

11.2.1 Optional arguments

- `--model_path, type=str, default='models/active_subnetwork_KO_5.xml`. Relative or absolute path to an SBML model (.xml-file)
- `--direct, type=str2bool, default=False`. Enable to intersect with equalities directly. Direct intersection works better than indirect when many metabolites are hidden, and on large networks (default: False)
- `--compress, type=str2bool, default=True`. Perform compression to which the conversions are invariant, and reduce the network size considerably (default: True)
- `--out_path, default='conversion_cone.csv'`. Relative or absolute path to the .csv file you want to save the calculated conversions to (default: `conversion_cone.csv`)
- `--add_objective_metabolite, type=str2bool, default=True`. Add a virtual metabolite containing the stoichiometry of the objective function of the model (default: true).
- `--print_metabolites, type=str2bool, default=True`. Print the names and IDs of metabolites in the (compressed) metabolic network (default: true)
- `--print_reactions, type=str2bool, default=False`. Print the names and IDs of reactions in the (compressed) metabolic network (default: true)
- `--print_conversions, type=str2bool, default=True`. Print the calculated conversion modes (default: true)
- `--use_external_compartment, type=str, default=None`. If a string is given, this string indicates how the external compartment in metabolite-ids of SBML-file is marked. By default, dead-end reaction-detection is used to find external metabolites, and not compartment-information. Please check if external compartment detection works by checking metabolite information before compression and with `--print_metabolites true`
- `--auto_direction, type=str2bool, default=True`. Automatically determine external metabolites that can only be consumed or produced (default: true)
- `--inputs, type=str, default=''`. Comma-separated list of external metabolite indices, as given by `--print_metabolites true` (before compression), that can only be consumed
- `--outputs, type=str, default=''`. Comma-separated list of external metabolite indices, as given by `--print_metabolites true` (before compression), that can only be produced. If inputs are given, but no outputs, then everything not marked as input is marked as output. If inputs and outputs are given, the possible remainder of external metabolites is marked as both
- `--hide, type=str, default=''`. Comma-separated list of external metabolite indices, as given by `--print_metabolites true` (before compression), that are transformed into internal metabolites by adding bidirectional exchange reactions
- `--prohibit, type=str, default=''`. Comma-separated list of external metabolite indices, as given by `--print_metabolites true` (before compression), that are transformed into internal metabolites without adding bidirectional exchange reactions. This metabolite can therefore be used as neither input nor output.
- `--tag, type=str, default=''`. Comma-separated list of reaction indices, as given by `--print_reactions true` (before compression), that will be tagged with new virtual metabolites, such that the reaction flux appears in ECMs.
- `--hide_all_in_or_outputs, type=str, default=''`. String that is either empty, input, or output. If it is input or output, after splitting metabolites, all inputs or outputs are hidden (objective is always excluded.

- `--iterative, type=str2bool, default=False`. Enable iterative conversion mode enumeration (might help on large, dense networks) (default: false)
- `--only_rays, type=str2bool, default=False`. Enable to only return extreme rays, and not elementary modes. This describes the full conversion space, but not all biologically relevant minimal conversions. See: Clement, 2020 and Urbanczik, 2005.
- `--verbose, type=str2bool, default=True`. Enable to show detailed console output (default: true)
- `--splitting_before_polco, type=str2bool, default=True`. Enables splitting external metabolites by making virtual input and output metabolites before starting the computation. Setting to false would do the splitting after first computation step. Which method is faster is complicated and model-dependent. (default: true)
- `--redund_after_polco, type=str2bool, default=True`. (Indirect intersection only) Enables redundant row removal from inequality description of dual cone. Works well with models with relatively many internal metabolites, and when running parrallelised computation using MPI (default: true)
- `--scei, type=str2bool, default=True`. Enable to use SCEI compression (default: true)
- `--sort_order, type=str, default='min_adj'`. Order in which internal metabolites should be set to zero during direct intersection. Default is to minimize the added adjacencies, other options are: `min_lp`, `max_lp_per_adj`, `min_connections`
- `--intermediate_cone_path, type=str, default=''`. Filename where intermediate cone result can be found. If an empty string is given (default), then no intermediate result is picked up and the calculation is done in full.
- `--manual_override, type=str, default=''`. (Advanced option). Index indicating which metabolite should be intersected in first step. Can be used in combination with `--intermediate_cone_path` to pick a specific intersection at a specific step.

Example

```
python3 main.py --model_path models/e_coli_core.xml --auto_direction true --
    out_path core_conversions.csv
```

11.3 Mode 2: Python library

`Ecmtool` can also be used as a separate programming interface from within your own Python code. To do so, install `ecmtool` using *pip* (e.g. *pip install ecmtool*). The most crucial method is ecmtool.conversion_cone:get_conversion_cone(), which returns the ECMs of a given stoichiometric matrix. For information on how to use advanced features like SBML parsing, network compression, and metabolite direction estimation, please see ecmtool/main.py.

Example

```
from ecmtool.network import extract_sbml_stoichiometry
from ecmtool.conversion_cone import get_conversion_cone

network = extract_sbml_stoichiometry('models/sxp_toy.xml', add_objective=True)
stoichiometry = network.N

ecms = get_conversion_cone(stoichiometry, network.external_metabolite_indices(),
 network.reversible_reaction_indices(), network.input_metabolite_indices(),
 network.output_metabolite_indices())

```

11.4 Advanced usage

After testing how the tool works, most users will want to run their workloads on computing clusters instead of on single machines. This section describes some of the steps that are useful for running on clusers

Doubling direct enumeration method speed

The direct enumeration method can be sped up by compiling our LU decomposition code with Cython. The following describes the steps needed on Linux, but the same concept also applies to Mac OS and Windows. First make sure all dependencies are satisfied. Then execute:

```
python3 setup.py build_ext --inplace

mv _bglu* ecmtool/
```

Running on a computing cluster with mpiexec

For example:

```
mpiexec -n 4 python3 main.py --model_path models/e_coli_core.xml
```

Examples of run commands and necessary computing power

In this part, we provide some descriptions on how the presented results were obtained. All ECMs that were computed are supplied as supplementary files.

Escherichia coli-model: e_coli_core

This model, downloadable from `bigg.ucsd.edu`,[19] is excellent for getting to know the workings of `ecmtool`, because the runtime is quite short. An example runscript is given by:

```
python3 main.py --model_path models/e_coli_core.xml --auto_direction true --
    direct false --splitting_before_polco true
```

It is good to get a feel for the different enumeration options, such as `--direct`, `--splitting_before_polco` and `--redund_after_polco`. The enumeration should always give the ECMs that can also be found in the file `conversions_ecolicore.csv`.

In the main text, we also show the ECMs obtained for this model when all outputs were hidden. This can be achieved by running

```
python3 main.py --model_path models/e_coli_core.xml --direct False --
    hide_all_in_or_outputs output
```

In fact, this command provides a shortcut to focus on only inputs, but one could also obtain this result with giving all indices of output metabolites to the `--hide`-argument.

If run with the argument `--print_reactions true`, `ecmtool` prints an indexed list of reactions before starting the computation. This can be used if a specific reaction is of interest. For example, in the main text we showed results in which the activity of the pyruvate dehydrogenase-reaction was reported. In the printed list one can see that this is reaction 50. We can therefore run

```
python3 main.py --model_path models/e_coli_core.xml --direct False --
    hide_all_in_or_outputs output --tag 50
```

Helicobacter pylori-model: iIT341

The `iIT341`-model is also available at `bigg.ucsd.edu`. The enumeration of all ECMs of this model is quite computationally intense. The results shown in Supplementary Figure 1, and made available in `iIT_minII_fullconversioncone.zip`, were calculated on a Linux-based virtual computing cluster with the command:

```
mpiexec -n 4 python3 main.py
--model_path models/iIT341.xml --direct false
--inputs 139,262,28,294,300,306,314,231,35,350,259,261,22,356,334,93,293,271
--out_path iIT.csv
--outputs 16,26,29,33,39,40,59,65,75,81,90,100,110,145,171,174,212,223,224,232,
234,235,239,252,253,255,263,265,269,276,277,279,280,283,284,286,291,296,302,308,
312,319,320,323,325,329,331,336,341,342,344,345,352,358,361,366,368,370,372,293
--splitting_before_polco false
```

Running with `mpiexec -n 4`, implies that some of the tasks are spread over 4 computation cores. For this model, it turned out to be beneficial to use the indirect method, `--direct false`. When we use indirect intersection, only the redundancy removal can be parallelised. The indices for the `inputs`- and `outputs`-arguments were determined by first running `ecmtool` with the default argument `--print_metabolites true`. In the printed list with indices and metabolites, we could find all metabolites that were mentioned by the model developers[1] in a minimal medium. We used `--splitting_before_polco false`, simply because this seemed to enable faster progress. For now, we cannot really determine when it is wise to use this option or not, so that trial and error is the best we can do. This computation took several hours to run.

If one is mostly interested in the conversion of inputs to biomass, it might be sufficient to calculate the ECMs in the network while all outputs are hidden. In Figure 5 of the main text, we show the results obtained by this command:

```
--model_path models/iIT341.xml --direct false
--inputs 139,262,28,294,300,306,314,231,35,350,259,261,22,356,334,93,293,271
--out_path iIT.csv
--outputs 16,26,29,33,39,40,59,65,75,81,90,100,110,145,171,174,212,223,224,232,
234,235,239,252,253,255,263,265,269,276,277,279,280,283,284,286,291,296,302,308,
312,319,320,323,325,329,331,336,341,342,344,345,352,358,361,366,368,370,372,293
--hide_all_in_or_outputs output
```

So, we added the argument –hide_all_in_or_outputs output, and removed `--splitting_before_polco false`. The first ensures that all output-metabolites are hidden. Hiding these output metabolites is only possible if metabolites that are both in- and output are already split before the enumeration step. Therefore, the argument `--splitting_before_polco false` is overridden by `ecmtool` anyhow.

Escherichia coli-model: iJR904

This large *E. coli*-model can also be downloaded from `bigg.ucsd.edu`. We used it to calculate all relations between glucose and oxygen consumption, and biomass production. These relations are shown in Figure 6 of the main text. The computation must be done using the direct method, and on many computation cores. We have used 20 nodes each of which had 16 computing cores, and the computation took 14.5 hours. The full jobscript that we used was:

```
#!/bin/bash
#SBATCH -N 20
#SBATCH -t 48:00:00

module load 2019
module load Python/3.6.6-intel-2019b
export PATH=$PATH:~/lrslib/lrslib-070

mpiexec python3 ~/ecmtool/main.py --model_path ~/ecmtool/models/iJR904.xml
--inputs 374,542,551,589,660
--hide 542,589,660,3,6,20,27,65,68,77,120,122,124,130,133,136,144,146,
164,167,169,173,180,189,192,195,197,206,211,219,223,229,233,236,242,244,247,
249,260,265,268,280,286,288,305,315,324,328,333,336,338,340,349,352,354,356,
358,360,363,366,376,378,380,382,388,393,395,397,399,402,404,413,419,424,427,
431,436,447,452,455,459,462,466,474,476,479,481,490,493,497,500,502,504,506,
509,510,513,515,517,525,532,534,536,542,545,547,549,555,562,583,589,592,603,
611,616,623,633,638,643,649,651,660,662,668,674,680,682,688,692,695,697,699,
704,707,709,711,714,742,745,747,750,752,755,759
```

```
--out_path ~/output/iJR.csv
--direct true
```

One might notice that the lists of metabolites to hide can become quite long, and therefore it might be tedious to manually compile these. We therefore first used `ecmtool` as a Python library, and created a small script that returned lists of the metabolite indices that should be hidden.

Saccharomyces cerevisiae-model: iND750

This model, also downloaded from `bigg.ucsd.edu`, was analysed in the paper that originally introduced ECMs.[10] We found that the import and export of many external metabolites was prohibited by the authors. This is also possible with the `ecmtool`-argument `--prohibit`. It makes sure that only the ECMs are returned that do not involve one of the prohibited metabolites. Although this functionality enables the calculation of some ECMs for very large models, it might not be very biologically reasonable.

Our runscript and the computed ECMs can be found in the supplementary files `iND750_runscript.txt` and `iND750_indirect.csv`.

References

[1] Thiele I, Vo TD, Price ND, Palsson B. Expanded metabolic reconstruction of Helicobacter pylori (iIT341 GSM/GPR): An in silico genome-scale characterization of single- and double-deletion mutants. Journal of Bacteriology. 2005;187(16):5818–5830.

[2] Matoušek J, Gärtner B. Understanding and Using Linear Programming. Springer Verlag, New York; 2007.

[3] Terzer M. Large scale methods to enumerate extreme rays and elementary modes. ETH Zurich; 2009.

[4] Rockafellar RT. Convex Analysis. vol. 28. Princeton University Press; 1970.

[5] Fukuda K. Frequently asked questions in polyhedral computation; 2004. https://inf.ethz.ch/personal/fukudak/polyfaq/polyfaq.html.

[6] Fukuda K, Prodon A. Double description method revisited. In: Combinatorics and computer science. vol. 1120. Springer; 1996. p. 91–111.

[7] Motzkin TS, Raiffa H, Thompson GL, Thrall RM. The double description method: Contributions to the theory of games. Annals of Mathematics Studies. 1953;28:51–73.

[8] Avis D. lrslib, version 6.0; 2015.

[9] Terzer M. Polco: A Java tool to compute extreme rays of polyhedral cones; 2009.

[10] Urbanczik R, Wagner C. Functional stoichiometric analysis of metabolic networks. Bioinformatics. 2005;21(22):4176–4180.

[11] Müller S, Regensburger G. Elementary vectors and conformal sums in polyhedral geometry and their relevance for metabolic pathway analysis. Frontiers in Genetics. 2016;7:90.

[12] Klamt S, Regensburger G, Gerstl MP, Jungreuthmayer C, Schuster S, Mahadevan R, et al. From Elementary Flux Modes to elementary flux vectors: Metabolic pathway analysis with arbitrary linear flux constraints. PLoS computational biology. 2017;13(4):e1005409.

[13] Nocedal J, Wright SJ. Numerical Optimization. 2nd ed. New York, NY, USA: Springer; 2006.

[14] Bertsimas D, Tsitsiklis JN. Introduction to Linear Optimization. Athene Scientific, Belmont, Massachusetts; 1997.

[15] Dalcin LD, Paz RR, Kler PA, Cosimo A. Parallel distributed computing using Python. Advances in Water Resources. 2011;34(9):1124–1139.

[16] Orth J Fleming R PB. Reconstruction and use of microbial metabolic networks: the core *Escherichia coli* metabolic model as an educational guide. EcoSal Plus. 2010;.

[17] Reed JL, Vo TD, Schilling CH, Palsson BO. An expanded genome-scale model of *Escherichia coli* K-12 (iJR904 GSM/GPR). Genome biology. 2003;4(9):R54–R54.

[18] Schulte CCM, Wheatley RM, Terpolilli JJ, Saalbach G, De Groot DH, Papachristodoulou A, et al. How legumes control nitrogen fixation by root nodule bacteria. Under revision. 2020;.

[19] King ZA, Lu J, Dräger A, Miller P, Federowicz S, Lerman JA, et al. BiGG Models: A platform for integrating, standardizing and sharing genome-scale models. Nucleic Acids Research. 2016;44(D1):D515–D522.
